# Functionally distinct promoter classes initiate transcription via different mechanisms reflected in focused versus dispersed initiation patterns

**DOI:** 10.15252/embj.2023113519

**Published:** 2023-04-04

**Authors:** Leonid Serebreni, Lisa‐Marie Pleyer, Vanja Haberle, Oliver Hendy, Anna Vlasova, Vincent Loubiere, Filip Nemčko, Katharina Bergauer, Elisabeth Roitinger, Karl Mechtler, Alexander Stark

**Affiliations:** ^1^ Research Institute of Molecular Pathology (IMP), Vienna BioCenter (VBC), Campus‐Vienna‐Biocenter 1 Vienna Austria; ^2^ Institute of Molecular Biotechnology (IMBA), Vienna BioCenter (VBC) Vienna Austria; ^3^ Medical University of Vienna Vienna BioCenter (VBC) Vienna Austria

**Keywords:** promoters, RNA polymerase II preinitiation complex, transcription initiation, Chromatin, Transcription & Genomics, Methods & Resources

## Abstract

Recruitment of RNA polymerase II (Pol II) to promoters is essential for transcription. Despite conflicting evidence, the Pol II preinitiation complex (PIC) is often thought to have a uniform composition and to assemble at all promoters via an identical mechanism. Here, using *Drosophila melanogaster* S2 cells as a model, we demonstrate that different promoter classes function via distinct PICs. Promoter DNA of developmentally regulated genes readily associates with the canonical Pol II PIC, whereas housekeeping promoters do not, and instead recruit other factors such as DREF. Consistently, TBP and DREF are differentially required by distinct promoter types. TBP and its paralog TRF2 also function at different promoter types in a partially redundant manner. In contrast, TFIIA is required at all promoters, and we identify factors that can recruit and/or stabilize TFIIA at housekeeping promoters and activate transcription. Promoter activation by tethering these factors is sufficient to induce the dispersed transcription initiation patterns characteristic of housekeeping promoters. Thus, different promoter classes utilize distinct mechanisms of transcription initiation, which translate into different focused versus dispersed initiation patterns.

## Graphical abstract

Analyses in *Drosophila* S2 cells reveal differential transcription factor requirements at housekeeping versus developmental gene promoters, translating into focused or dispersed transcription patterns.

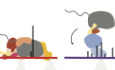

## Introduction

Transcription of protein‐coding genes by RNA polymerase II (Pol II) is a highly regulated process orchestrated by noncoding regulatory elements, namely enhancers and promoters. Pol II recruitment at promoters leads to transcription initiation from the core promoter region, a roughly 100 base‐pair region around the transcription start site (TSS) at the 5′ end of protein‐coding genes (Butler & Kadonaga, 2002). Although core promoter DNA fragments on their own are typically not sufficient for activity *in vivo* and support only low levels of transcription *in vitro* (Juven‐Gershon & Kadonaga, 2010), the TATA‐box core promoter is sufficient to bind the TATA‐binding protein (TBP) and assemble the Pol II preinitiation complex (PIC; Buratowski *et al*, 1989; Geiger *et al*, 1996; Petrenko *et al*, 2019; see also below). This finding suggests that the core promoter DNA sequence has a crucially important function for PIC assembly and transcription and made the TATA‐box core promoter subtype a prominent model for studies of PIC assembly and transcription initiation (Smale & Kadonaga, 2003).

**Figure 1:**
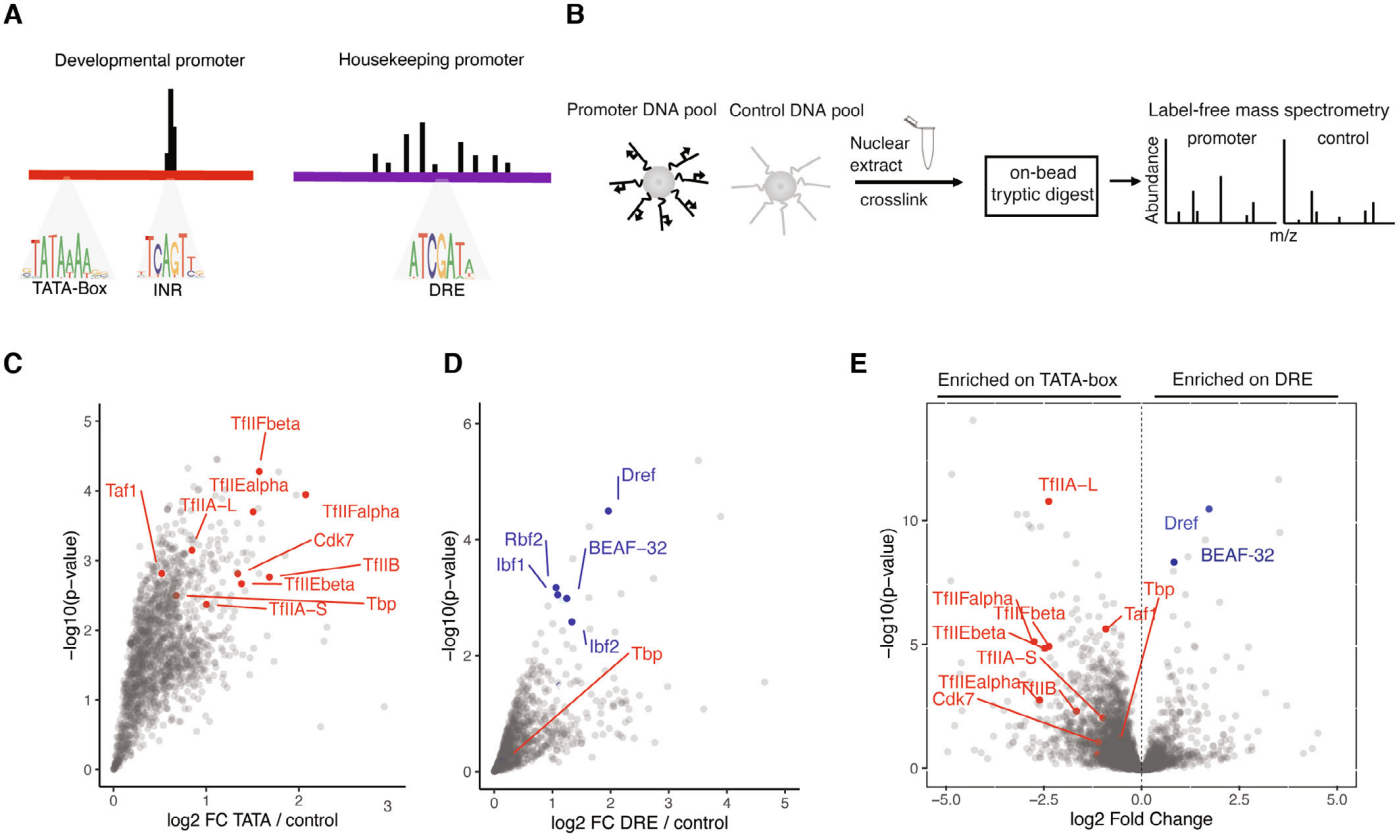
DNA affinity purifications uncover differentially bound proteins at functionally distinct promoters A
Examples of stereotypical 121‐bp‐long core promoters used for DNA affinity purification of a developmental TATA‐box type, and a DRE motif‐containing housekeeping type. TATA‐box promoters exhibit focused transcription initiation from the INR motifs focused around 1–3 bp, while DRE‐containing promoters exhibit dispersed transcription initiation across 50–100 bp.B
Scheme of DNA affinity purification coupled to label‐free mass spectrometry. Promoters are analyzed in pools and enrichment is measured against a pool of negative control regions from the *Drosophila* genome.C, D
(C) Enrichment of proteins detected by mass spectrometry on a pool of TATA‐box promoters over control DNA sequences, and in (D) over a pool of DRE promoters. Three biological replicates were performed for each promoter and control pool and significance measured with a Limma *P*‐value < 0.05.E
Enrichment of proteins bound to DRE promoters over TATA‐box promoters. Limma *P*‐value < 0.05. This comparison is generated by performing a ratio of abundance of proteins binding to DRE promoters directly over the abundance on TATA‐box promoters.

Based on multiple lines of evidence, promoters in *Drosophila melanogaster* can be categorized into two broad classes (i) developmental promoters of developmentally regulated or cell‐type‐restricted genes that contain TATA‐boxes, downstream promoter elements (DPEs), and/or Initiator (INR) motifs (Ohler *et al*, 2002; Carninci *et al*, 2006; Lenhard et al, 2012; Vo Ngoc *et al*, 2017, 2020) and (ii) housekeeping promoters of broadly or ubiquitously expressed genes that contain TCT, DRE, and Ohler1/6 motifs (Fig 1A). These two classes of promoters exhibit distinctive regulatory properties, respond differently toward activating cues (Zabidi *et al*, 2015; Arnold *et al*, 2016), and are activated by distinct sets of coactivators (Haberle *et al*, 2019). In addition, developmental promoters typically display focused initiation at a single, dominant TSS, whereas housekeeping promoters typically display dispersed initiation at multiple TSSs (Rach *et al*, 2011).

The general transcription factors (GTFs: TFIIA, TFIIB, TFIID, TFIIE, TFIIF, and TFIIH) assemble the PIC hierarchically at TATA‐box core promoters: the TATA‐binding protein (TBP) within TFIID binds to the TATA‐box motif in promoter DNA and recruits TFIIA, followed by the remaining GTFs (Orphanides *et al*, 1996; Cosma, 2002; He *et al*, 2013; Mühlbacher *et al*, 2014) and Pol II. TFIIA cooperates with TFIID to commit PIC assembly into an active state on promoters *in vitro* (Buratowski *et al*, 1989; Papai *et al*, 2010; Warfield *et al*, 2017). However, the nature of the PIC and PIC assembly at different core promoter subtypes and whether they relate to these promoters' distinct functions, remain unknown; moreover, the distinct properties of core promoter subtypes seem incompatible with a single mechanism of PIC assembly and transcription initiation.

Some evidence indeed suggests that different promoters utilize different PIC components. For example, some cells do not seem to require TBP (Wieczorek *et al*, 1998; Martianov *et al*, 2002; Gazdag *et al*, 2016; Kwan *et al*, 2021), and some promoters require only a subset of GTFs for transcription *in vitro* (Parvin *et al*, 1992, 1994) or in cells (Santana *et al*, 2022), which is in line with the existence of different stable intermediates or alternative arrangements of the PIC on promoter DNA (Buratowski *et al*, 1989; Wieczorek *et al*, 1998; Yudkovsky *et al*, 2000; Murakami *et al*, 2013; Yu *et al*, 2020). Further, promoter‐bound multi‐subunit protein complexes that are part of the PIC, such as TFIID, can exhibit different arrangements. For instance, the Taf9 subunit of TFIID regulates cell‐type‐specific genes in neural stem cells (Neves & Eisenman, 2019), whereas the Taf3 subunit of TFIID activates cell‐type‐specific genes in myoblasts (Stijf‐Bultsma *et al*, 2015).

In addition, some GTFs might not be required in all cells (Tyree *et al*, 1993; Ranish *et al*, 1999; Martianov *et al*, 2002; Cabart *et al*, 2011; Gazdag *et al*, 2016; Kwan *et al*, 2021) and/or GTF paralogs may regulate transcription in distinct cell types or at specific promoters (Akhtar & Veenstra, 2011; Duttke *et al*, 2014; Zehavi *et al*, 2015). The TBP‐related factors TBP2 (also known as TRF3) and TBPL1 (TRF2 in *Drosophila*) have, for example, been implicated in transcription in early steps of mouse oocyte differentiation and during spermatogenesis, respectively (Zhang *et al*, 2001; Gazdag *et al*, 2016; Martianov *et al*, 2016; Yu *et al*, 2020), In *Drosophila*, Trf2 has been suggested to regulate the transcription of ribosomal protein genes, histone H1, and DPE motif‐containing promoters (Isogai *et al*, 2007; Wang *et al*, 2014; Baumann & Gilmour, 2017; Kedmi *et al*, 2020). This cumulative evidence suggests that different promoter‐bound GTF assemblies may exist on different promoter types and/or in different cell types, which potentially relates to these promoters' distinct properties.

Here, we used DNA affinity purification to identify proteins that closely interact with core promoters, combined with protein depletion and PRO‐seq to identify proteins that are required for the transcriptional function of core promoters. We found differential use of TBP and Trf2 at different promoter subtypes and discovered distinct recruitment mechanisms of TFIIA: TFIIA was enriched at developmental promoters *in vitro* and required for their activity *in vivo*, suggesting a direct recruitment mechanism and compact PIC architecture at this promoter class. In contrast, TFIIA was not enriched at housekeeping promoters *in vitro* but still required for their activity *in vivo*, suggesting an indirect recruitment mechanism and/or dispersed PIC architecture at these promoters. Our work suggests that direct recruitment of TFIIA at developmental promoters leads to their focused initiation pattern, whereas indirect recruitment of TFIIA at housekeeping promoters leads to their dispersed initiation pattern.

## Results

### *In vitro* DNA affinity purification detects core promoter DNA–protein interactions

**Figure EV1:**
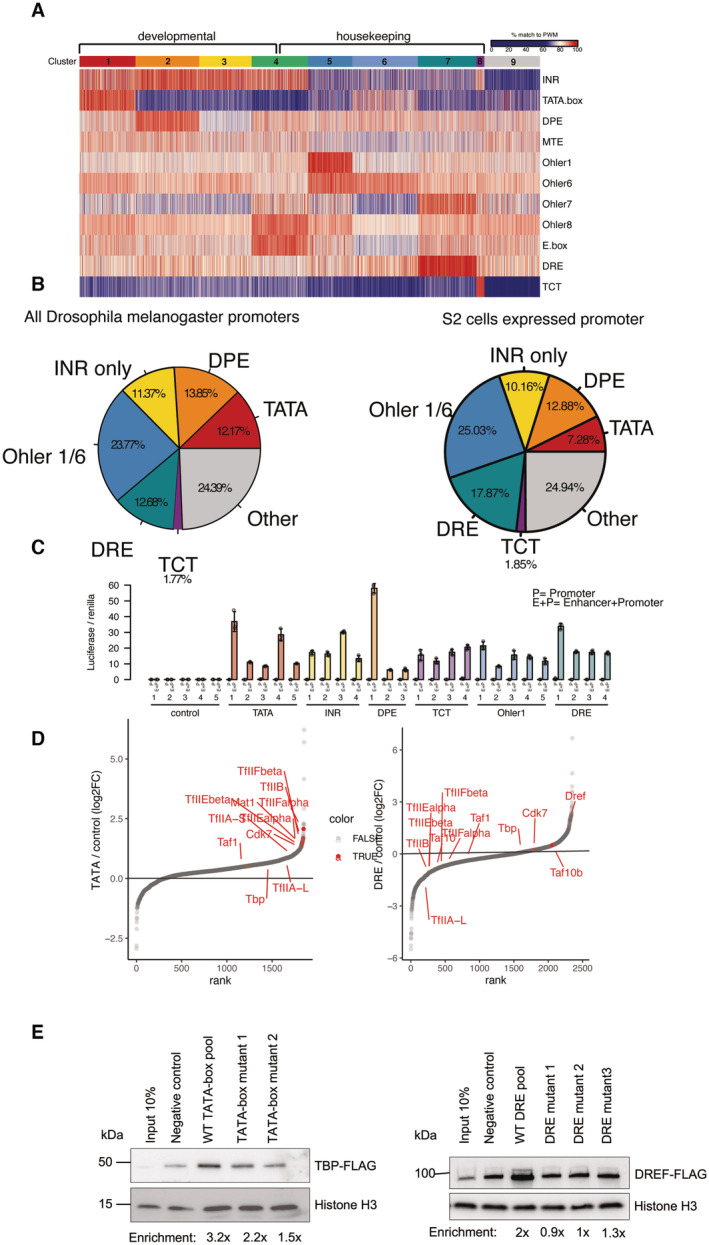
DNA affinity purifications uncover differentially bound proteins at functionally distinct promoters Heat map of ~17,000 *Drosophila melanogaster* protein‐coding gene promoters displaying % match to position weight matrix (PWM) score. Promoters were clustered with k‐means clustering. Nine clusters emerged which display developmental (clusters 1–3) and housekeeping (clusters 5–7) motifs. Promoters in cluster 4 are enriched in Ohler8 and E‐box motifs and can respond to both developmental and housekeeping coactivators as defined by Haberle, V. *et al*, 2019. Cluster 9 had no strong matches to any motif PWM.Pie chart of all expressed *Drosophila melanogaster* protein‐coding gene promoters (~170,000) grouped based on motif content (left), and all expressed protein‐coding genes from *Drosophila* S2 cells (~10,000). Only the main motif groups studied in this paper that are classified as housekeeping or developmental are shown. Group labeled as “other” contains promoters with motifs such as Ohler 8 and E‐box or not motifs which could not be assigned as developmental or housekeeping.Luciferase activity assay measuring the basal or activated state of tested core promoter fragments. To measure basal activity, 121‐bp‐long promoter fragments cloned upstream of a luciferase gene (P). To measure the activated state of the core promoters we cloned the *Drosophila* Zdfh1 enhancer upstream of the promoter fragments (E + P). Plasmids were transfected into *Drosophila* S2 cells and activity was measured after 48 h. Firefly luciferase values were normalized to co‐transfected Renilla luciferase values to control for transfection efficiency. Error bars represent standard deviation across four biological replicates.Rank plot of protein binding enrichment on TATA and DRE promoters over the control DNA pool from the DNA‐purification mass spectrometry assay. Highlighted proteins are the Pol II PIC components and the DRE binding factor DREF.DNA‐purification assay with a pool of 25 TATA‐box promoters, and two individual TATA‐box promoters in which the TATA‐box was mutated (left panel). The assay was performed with a nuclear extract expressing TBP‐FLAG that was tracked with a western blot. DNA‐purification of a pool of 20 DRE promoters and three individual DRE promoters in which the DRE motif was mutated. The assay was performed with a nuclear extract expressing DREF‐FLAG and followed with a western blot (right panel). Note that DREF binding is reduced to background levels while TBP is still slightly enriched compared with negative controls, consistent with TBP binding to non‐TATA‐box developmental core promoters (Fig 2B and E).

Roughly 37% of core promoters in the *Drosophila* genome can be classified as developmental (TATA + INR, DPE + INR, INR only), and 38% as housekeeping (Ohler1/6, DRE, TCT), based on previous work by others and us (Fig EV1A and B; Ohler *et al*, 2002; Lenhard *et al*, 2012; Haberle & Stark, 2018; Vo Ngoc *et al*, 2019). Given the distinct sequences and regulatory functions of these two types of core promoters, we hypothesized that the core promoter DNA directly binds to different transcription‐related proteins. Using TATA‐box core promoters (which also contain the INR motif at the TSS) as positive control and reference point, we reasoned that short (121 bp) core promoter DNA fragments of the different core promoter types might differ in their ability to recruit transcription‐related proteins and that these could be identified *in vitro*, using conditions that assemble the canonical PIC on TATA‐box promoters *in vitro* (Kadonaga & Tjian, 1986; Kamakaka *et al*, 1991; Nikolov *et al*, 1995; Geiger *et al*, 1996; Tan *et al*, 1996; Johnson *et al*, 2004; Baek *et al*, 2006; Lin & Carey, 2012; Plaschka *et al*, 2015). We therefore selected core promoter fragments that are not themselves transcriptionally active, yet are readily inducible by activators (such as strong enhancer elements) to drive high levels of transcription in luciferase assays (Fig EV1C).

**Figure 2:**
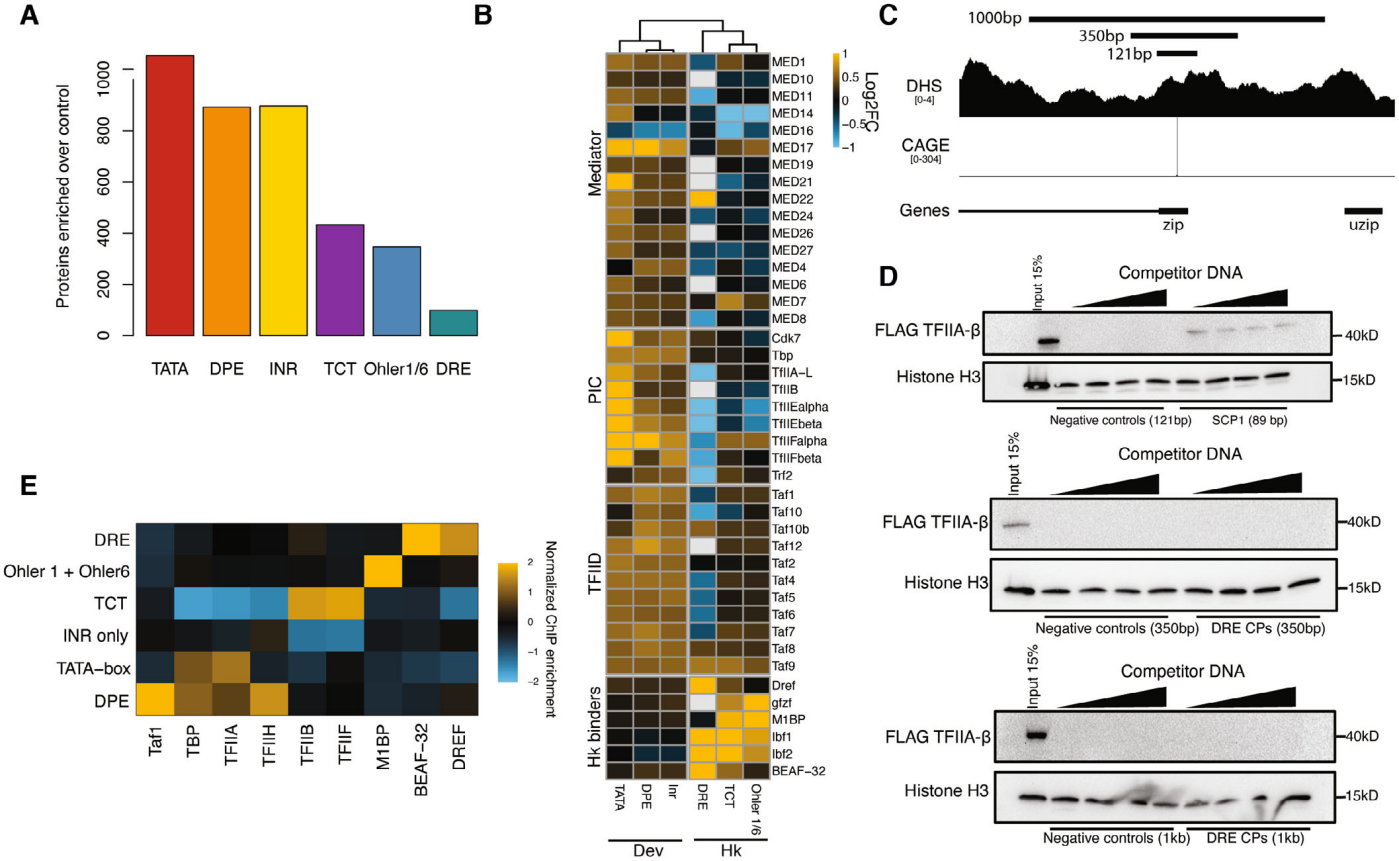
Developmental and housekeeping promoters bind different sets of proteins and GTFs Total number of enriched proteins on the different tested pooled promoter types from the DNA affinity purification mass spectrometry (Limma *P*‐value < 0.05 enrichment > 0).Enrichments from DNA affinity purification mass spectrometry of selected proteins and protein complexes on the tested different pooled promoter types compared with negative control DNA. White represents protein not detected in the given sample. The promoters were clustered by hierarchical clustering of the mass spectrometry enrichments based on Euclidian distances, which supported the split between developmental and housekeeping core promoters (dendrogram). Three biological replicates per condition with a Limma *P*‐value < 0.05.Tested regions around the zip promoter that were used in DNA affinity purification and luciferase assay around the zip gene promoter. CAGE and DHS indicate that this promoter is accessible and transcribed in S2 cells.Western blots of the DNA‐bound fraction eluted off the beads in a DNA affinity purification assay. Super core promoter 1 (SCP1) was used as a positive control to bind TFIIA‐β‐FLAG (top panel). DRE promoter pools of varying lengths were assayed for their ability to bind TFIIA. Sonicated salmon sperm DNA was used as competitor DNA and titrated from 100 ng to 1.6 μg per reaction. Histone H3 was used as an abundant nonspecific DNA interacting protein for loading control. From top to bottom we have used promoter fragments ranging at 121 bp, 350 bp and 1 kb in length.ChIP‐seq signal of GTFs and select housekeeping promoter binders from *Drosophila* S2 cells and embryos normalized to nascent transcription level as measured by PRO‐seq at the respective promoter types and converted to z‐scores.

First, we examined TATA‐box‐containing developmental core promoters and DRE‐containing housekeeping core promoter subtypes. To detect proteins that directly bind different promoter sequences of the same subtype, we pooled 16–32 representative core promoters per subtype and used a pool of 18 nonpromoter control DNA fragments as a negative control (Fig 1A and B). We coupled the fragments of each pool to streptavidin‐coated beads, incubated the beads with S2 cell nuclear extract and free competitor DNA, washed and cross‐linked associated proteins, and quantified the enriched proteins by label‐free mass spectrometry (Fig 1B). We performed three replicate experiments per pool and detected between 30 and 35 thousand peptides each, which allowed the label‐free quantification of 3,465 proteins in total across all samples. Using the three replicates, we detected 1,094 proteins significantly enriched at the TATA‐box core promoters over the control pool; and 98 proteins significantly enriched at the DRE core promoters (enrichment *P*‐value < 0.05; limma; Ritchie *et al*, 2015).

As expected from previous biochemical and structural work (Nikolov *et al*, 1995; Geiger *et al*, 1996; Tan *et al*, 1996; Plaschka *et al*, 2015), the TATA‐box‐containing core promoters were enriched for the canonical Pol II PIC, including TBP, GTFs and TFIID, and most Mediator subunits (Figs 1C and EV1D), confirming that TATA‐box promoter DNA is sufficient to directly bind these proteins *in vitro* and that our setup captures these protein‐DNA complexes.

Unexpectedly, the DRE‐containing core promoters did not enrich for any of the Pol II PIC subunits; indeed, some Tafs and GTFs were even depleted compared with control DNA. In contrast, the DRE core promoters were enriched for the core promoter‐element binding factor DREF, BEAF‐32, and Ibf1/2 among other proteins (Fig 1D). Directly plotting the enrichments at DRE versus TATA promoters confirmed the strong differential recruitment of GTFs and PIC components specifically to TATA promoters but not to DRE promoters (Fig 1E). Mutating either the TATA‐box or DRE motifs reduced TBP and DREF binding, respectively (Fig EV1E), suggesting that the differential binding of these proteins is directed by the different promoter DNA sequences as expected (Kwon *et al*, 2003; Tora & Timmers, 2010).

### Different promoter subtypes show distinct binding of the Pol II PIC

The *in vitro* DNA affinity purification detected an association between known PIC components and TATA‐box‐containing developmental core promoters, but not with housekeeping DRE core promoters. To determine whether the results above generalize to other promoter subtypes, we extended our analysis to additional developmental promoters containing DPE or INR motifs, and to housekeeping promoters containing TCT or Ohler 1/6 motifs.

We found that developmental promoter subtypes enriched for 892 to 1,093 proteins, whereas housekeeping promoter subtypes enriched only between 98 and 432 proteins (enrichment *P*‐value < 0.05; Fig 2A; Dataset EV1). Moreover, developmental and housekeeping promoters enriched for different sets of proteins: GTFs and PIC components were preferentially enriched at all developmental promoters but were not or only weakly enriched at housekeeping promoters (Fig 2B). Similarly, multiple components of the Mediator and TFIID complexes were preferentially enriched at developmental promoters, with TATA‐box‐containing promoters showing the highest levels of binding (Fig 2B). In contrast, none of the housekeeping promoter subtypes were enriched for GTFs, TFIID, or Mediator subunits; instead, they were enriched for various TFs that bind core promoter elements and chromatin regulators. For example, DRE‐containing promoters exhibited the highest enrichment of DREF and BEAF‐32, whereas Ohler 1/6 promoters exhibited the highest enrichment of the Motif 1‐binding protein (M1BP) and the cofactor GFZF (Fig 2B). The DNA affinity purification data suggest that short DNA fragments corresponding to functionally distinct core promoters directly associate with distinct transcription‐related proteins under identical conditions *in vitro*.

**Figure EV2:**
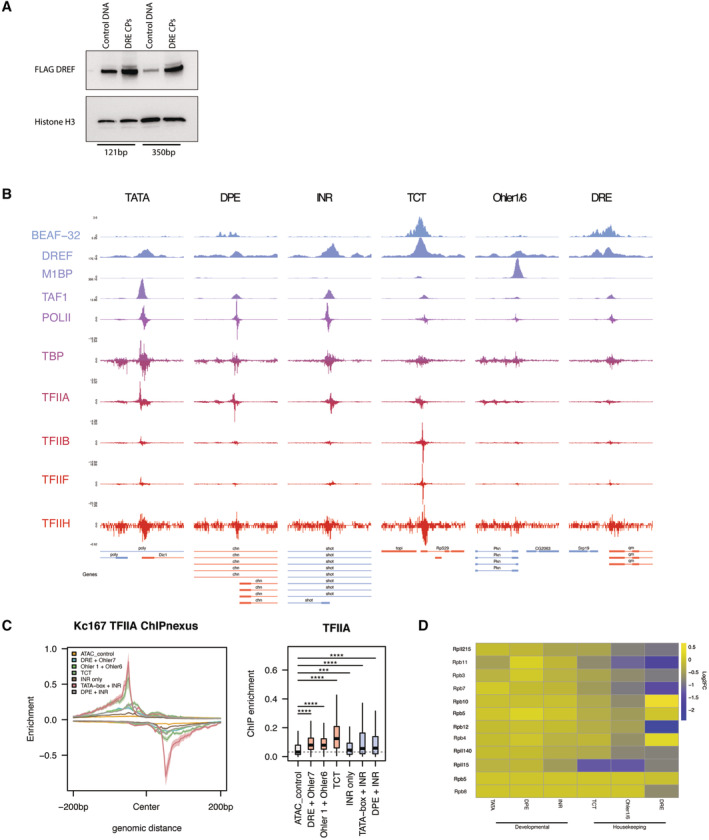
Developmental and housekeeping promoters bind different sets of proteins and GTFs Elution fractions from the DNA‐purification assay with a pool of 20,121 bp or a pool of 10,350 bp DRE promoters and length‐matched negative controls were performed with a nuclear extract expressing DREF‐AID‐3xFLAG tag and blotted for an anti‐FLAG antibody. Both promoter lengths are able to enrich for DREF binding.Representative browser tracks of published ChIP‐seq data of GTFs and promoter binding TFs (M1BP, DREF, BEAF‐32) on the 6 different tested promoter types in this study.Meta‐plot of TFIIA‐L ChIP‐seq data from panel E at the 6 different tested promoter types indicating TFIIA binds all active promoter types, although less strongly to housekeeping promoters and in a more dispersed fashion relative to the TSS (center). Box plot quantification of TFIIA ChIP‐seq data at /+ 200 bp around the TSS. Boxes represent the upper and lower quartiles, with the middle band at the median. The whiskers represent 1.5 times the interquartile range across two biological replicates, outliers not shown. (****P < 1e‐5, ***P < 1e‐3, **P < 1e‐2, *P < 5e‐2, N.S = not significant).Heat map of log2FC values of DNA affinity purification values for RNA polymerase II subunits across the six different promoter types tested.

We considered that 121 bp was not sufficiently long for the housekeeping core promoters to associate with the canonical PIC by DNA affinity purification. We thus tested 350‐ and 1,000‐bp‐long fragments derived from DRE promoters, which still did not interact detectably with the PIC component TFIIA‐β. In contrast, the TATA‐box‐containing SCP1 promoter, a well‐studied TATA‐box core promoter used as a positive control, readily interacted with TFIIA‐β (Fig 2C and D) and the 350‐bp‐long DRE promoter fragment interacted with DRE as expected (Fig EV2A). Overall, DNA affinity purification detected different sets of proteins that directly associate with housekeeping and developmental core promoter DNA under identical conditions *in vitro*. These findings are intriguing and suggest that the promoters' functional differences might arise at the level of GTF recruitment and PIC assembly, presumably via distinct DNA‐binding factors, tighter versus looser protein‐DNA complex architectures, and/or additional requirements such as nucleosome positioning or other chromatin features.

**Figure EV3:**
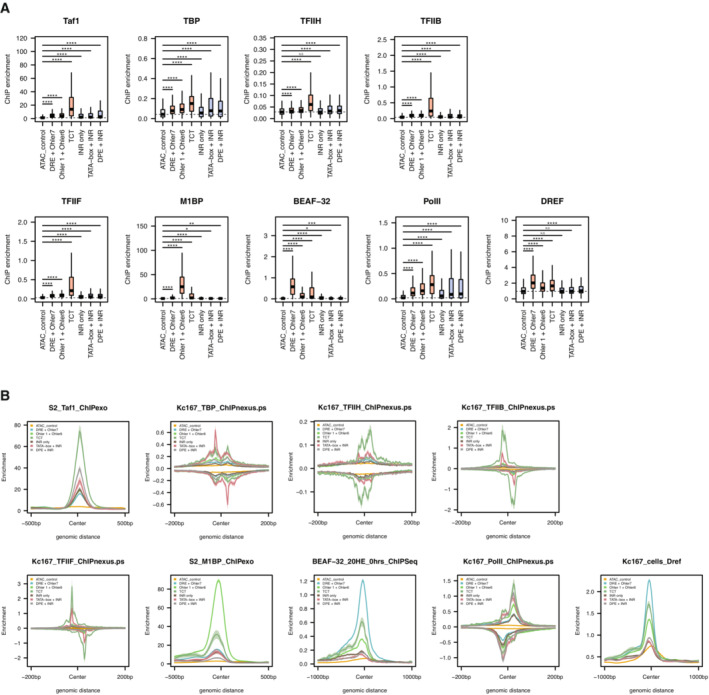
Distribution of GTFs at developmental and housekeeping promoter Box plots representing ChIP‐seq signal of available GTFs and sequence‐specific TFs on the six different promoter types tested from Drosophila melanogaster embryos centered on the TSS (200 bp to +200 bp). Wilcoxon test. Boxes represent the upper and lower quartiles, with the middle band at the median. The whiskers represent 1.5 times the interquartile range across two biological replicates, outliers not shown. (****P < 1e‐5, ***P < 1e‐3, **P < 1e‐2, *P < 5e‐2, N.S = not significant).Meta‐plot of ChIP‐seq signal of available GTFs and sequence‐specific TFs (as in panel H) on the six different promoter types centered on the TSS.

The DNA affinity purifications directly report the biochemical properties of the respective DNA fragments and suggest that core promoter DNA fragments differ in their ability to directly bind GTFs and the PIC *in vitro*. *In vivo*, additional players, such as chromatin, chromatin remodelers or nearby enhancers, can influence GTF or Pol II recruitment and transcription initiation at core promoters in ways that are not recapitulated by our assays. We reanalyzed published ChIP‐seq and ChIP‐nexus data from *Drosophila* cells or embryos, which confirmed that all the assayed GTFs do indeed bind to all promoters, including housekeeping promoters (Figs EV2B–D and EV3A–B). The ChIP signals however reflected the trends observed *in vitro* for the respective promoter subtypes (Fig 2E; Liang *et al*, 2014; Baumann & Gilmour, 2017; Shao & Zeitlinger, 2017): GTFs were generally more highly enriched at developmental promoters than housekeeping promoters (except for TFIIB and TFIIF that bound strongly to TCT promoters), whereas TFs were more highly enriched at housekeeping promoters according to their motif contents: M1BP showed the highest ChIP‐seq signals at Ohler 1/6 promoters, and DREF and BEAF‐32 showed highest signals at DRE promoters (Figs 2E and EV2C).

We infer that the DNA sequence of developmental core promoters forms a close/tight physical association with the PIC that can be detected by DNA affinity purification. In contrast, the weaker ChIP signals and lack of DNA affinity purification suggest a weaker/looser, less rigid, more transient, or more indirect physical association between housekeeping core promoter DNA and GTFs. Instead, housekeeping core promoters appear to form close physical associations with sequence‐specific TFs through their cognate DNA‐binding motifs both *in vitro* and *in vivo*. Additionally, the markedly lower number of proteins enriched at housekeeping promoters suggests that their DNA–protein interface is generally weaker, more indirect, and/or transient nature and that they might rely more on other features such as nucleosome positioning or other chromatin properties.

### Differentially recruited factors *in vitro* have distinct functional requirements

**Figure 3:**
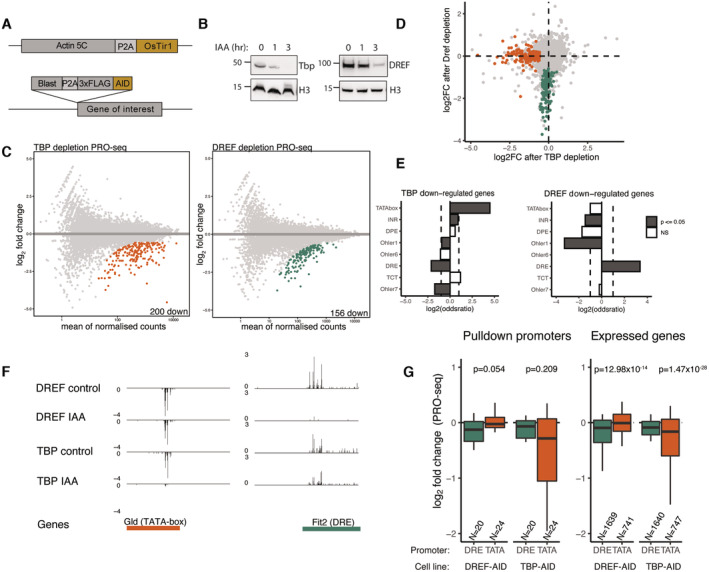
TBP and DREF are required by distinct sets of promoters Strategy for generating endogenously tagged AID cell lines. An AID‐3xFLAG endogenous knock‐in was generated in the N terminus of either DREF or TBP in a background cell line stably expressing the Tir1 ligase downstream of Actin5c.Western blot on FLAG‐tagged TBP and DREF 0,1 and 3 h after auxin addition showing protein degradation.PRO‐seq measurement after 6 h of auxin addition to the TBP or DREF AID‐tagged cell lines, MA plots represent colored genes which are significantly downregulated compared with no auxin control (fold change > 1.5 down; FDR < 0.05). Two biological replicates per conditions.Overlap of the TBP and DREF depletion PRO‐seq. Green and orange colored dots represent TBP‐ and DREF‐dependent promoters, fold change > 1.5 down & FDR < 0.05.Fisher's exact test for motif enrichment in TBP and DREF downregulated promoters compared with all expressed promoters. Log2 of the Odds ratio displayed. The dashed lines are set at a value of 1 and −1.Genome tracks of PRO‐seq data indicating examples of genes that are dependent on TBP or DREF. Glad is a gene with a TATA‐box promoter, while Fit2 is a gene with a DRE promoter.Differential PRO‐seq signal across TATA‐box and DRE promoters used for the DNA affinity purification (left) or all expressed TATA‐box and DRE motif‐containing promoters (right). *P*‐values from a two‐sided Wilcoxon test are provided (note that despite similar magnitude of change, the comparisons on the left are not significant due to low numbers of promoters in the compared groups). Boxes represent the upper and lower quartiles, with the middle band at the median. The whiskers represent the upper and lower 5^th^ percentiles.

To determine whether the differential recruitment of promoter‐associated factors *in vitro* reflects distinct functional requirements *in vivo*, we used the auxin‐inducible degron (AID) system (Nishimura *et al*, 2009) to deplete endogenously labeled proteins from *D. melanogaster* S2 cells and measured nascent transcription by PRO‐seq (Kwak *et al*, 2013), a strategy recently used for GTFs in human cells (Santana *et al*, 2022; Fig 3A).

We examined TBP and DREF first and observed the near complete degradation of both proteins 3 h after auxin addition (Fig 3B) and their complete depletion 6 h after auxin addition (Appendix Fig S1A). To ensure complete protein degradation while minimizing potential secondary effects from prolonged protein depletion, we measured changes to Pol II nascent transcription 6 h after auxin treatment.

We performed two biological replicates of PRO‐seq that were highly similar (PCC > 0.99 Appendix Fig S1B) and revealed 200 downregulated genes after TBP depletion and 156 downregulated genes after DREF depletion (fold change > 1.5 (down) and FDR < 0.05; Fig 3C). Notably, not a single gene was shared between the two conditions, indicating that distinct sets of promoters require TBP and DREF (Fig 3D). Motif enrichment analysis of the downregulated promoters revealed a strong enrichment of the TATA‐box in the TBP‐dependent promoters, and of the DRE motif in the DREF‐dependent promoters (Fig 3E), as expected. The differential dependency on TBP versus DREF is apparent at the TATA‐box promoter upstream of *Glucose dehydrogenase* (*Gld*) and the DRE promoter upstream of *Fermitin 2* (*Fit2*; Fig 3F) and generalizes to the promoters used for the DNA affinity purification experiments, and to all active TATA‐ versus DRE‐containing promoters genome‐wide (Fig 3G and Appendix Fig S1C). These results show that a relatively small number of active promoters require TBP (Martianov *et al*, 2002; Gazdag *et al*, 2016; Santana *et al*, 2022) and that these are specifically TATA‐box‐containing promoters. Similarly, only a subset of promoters requires DREF, which are different from the TBP‐requiring promoters and specifically contain DRE motifs. Overall, these results imply that different promoter types differentially depend on the two core promoter element binders and utilize distinct DNA–protein interfaces and/or interactors to recruit Pol II and initiate transcription.

### TBP and TRF2 display promoter subtype‐dependent requirements

**Figure EV4:**
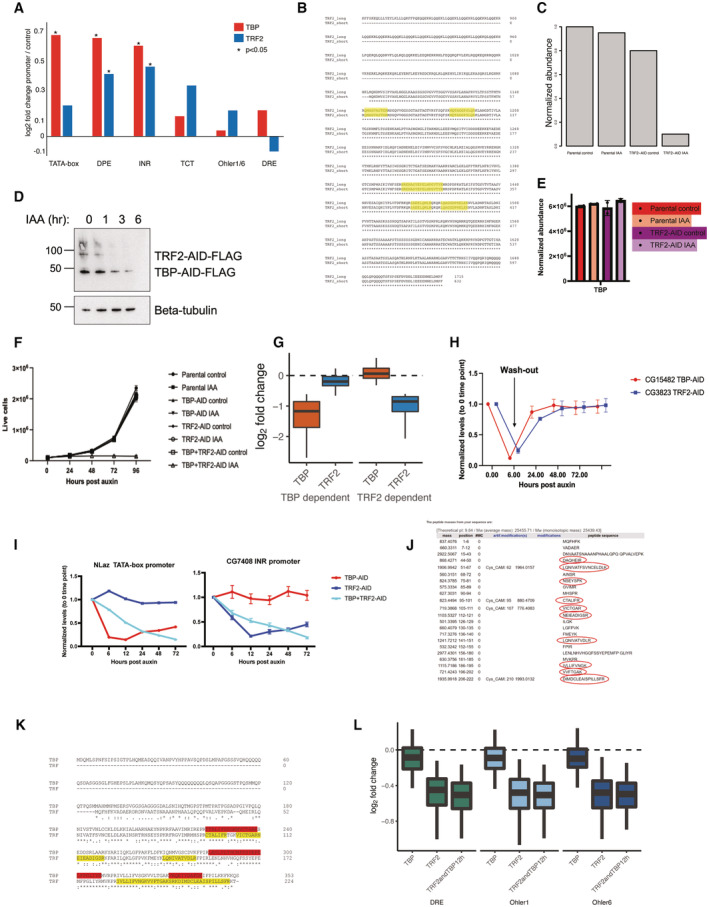
TBP and Trf2 regulate distinct subsets of developmental promoters DNA affinity purification mass spectrometry enrichment values of TBP and Trf2 across the tested promoter types. Student's *t*‐test (*P* < 0.05), three biological replicates per sample.ClustalW alignment of the short and long transcript isoforms of the *Drosophila melanogaster* TRF2 gene of the C‐terminal region from 840 to 1,715 amino acids. Peptides detected from label‐free mass spectrometric quantification of nuclear lysate from the TRF2‐AID cell line are highlighted in yellow. All detected peptides are shared between the two isoforms.Normalized abundance of TRF2 peptides from lab label‐free mass spectrometric quantification of nuclear lysates from the TRF2‐AID cell line. Parental cell line is expressing the Tir1 ligase, while the TRF2‐AID cell line is endogenously tagged with 3x‐FLAG‐AID. 500 μM Auxin treatment was performed for 6 h.Western blot of anti‐FLAG antibody on the double‐tagged TBP + Trf2 AID cell line visualizing TBP and Trf2 upon auxin addition, indicating a slower depletion kinetics of the TBP‐AID protein.Normalized abundance of TBP in the TRF2‐AID cell line and parental OsTir1 expressing cell line under control and 12 h 500 μM auxin treatment. Error bars represent the standard deviation across two biological replicates.Growth curve tracking the number of live cells for 4 days for individual TBP‐AID, Trf2‐AID, and double TBP + Trf2‐AID cell lines. No growth differences are observed upon the individually tagged cell lines, but the double TBP + Trf2‐AID cell line shows growth inhibition after addition of auxin. Error bars represent the standard deviation across three biological replicates.PRO‐seq signal after TBP or Trf2 depletion (log2 fold change) is plotted for the TBP‐dependent genes and Trf2‐dependent genes. Boxes represent the upper and lower quartiles, with the middle band at the median. The whiskers represent the upper and lower 5^th^ percentiles across two biological replicates.Auxin washout experiment in which TBP‐AID or Trf2‐AID cell lines were treated with auxin for 6 h and then washed twice and exchanged with fresh medium to remove auxin. qPCR performed on the tested time points on two tested genes indicate they can recover to their original level in the absence of auxin. Error bars represent standard deviation across three biological replicates.qPCR was performed on an auxin time‐course treatment experiment. The tested genes were normalized to Actin5c levels. NLaz was identified from PRO‐seq as dependent on TBP but not Trf2, and CG7408 was identified from PRO‐seq to be dependent on Trf2 but not TBP. Three biological replicates were performed, mean fold change (log2) over a Gal4‐DBD control of each sample is plotted with standard deviation with * for *P* ≤ 0.05.
*In silico* LyC and tryptic digestion of the Trf protein reveals predicted detectable peptides, which were not detected in mass spectrometry in our S2 cells, indicating a lack of Trf protein expression.ClustalW alignment of TBP and Trf. Peptides from TBP detected by mass spectrometry are highlighted in red. Peptides predicted from an *in silico* digest performed on Trf (from panel H) are highlighted in yellow.PRO‐seq data of individual TBP, Trf2 and double‐tagged TBP + Trf2 depletion at housekeeping promoters containing DRE, Ohler 1 and Ohler 6 motifs. These promoters are affected only upon depletion of Trf2 and to the same extent upon double depletion, demonstrating that TBP is dispensable for their expression and cannot substitute for Trf2 at these housekeeping promoters. Boxes represent the upper and lower quartiles, with the middle band at the median. The whiskers represent the upper and lower 5^th^ percentiles across two biological replicates.

As TBP seemed to be required only for TATA‐box‐containing promoters, we wondered whether TBP paralogs, specifically TRF2 (TBPL1 in mammals), might replace TBP at other promoter types (TRF, also called TRF1 is not detectable in S2 cells, Fig EV4G and H). In fact, TRF2 has been reported to function at DPE and TCT promoters in *Drosophila* (Wang *et al*, 2014; Zehavi *et al*, 2015; Kedmi *et al*, 2020) and we found TRF2 most strongly bound to DPE and INR containing core promoter DNA *in vitro* (Fig EV4A; TBP bound TATA‐box, DPE and INR promoters at equal levels).

**Figure 4:**
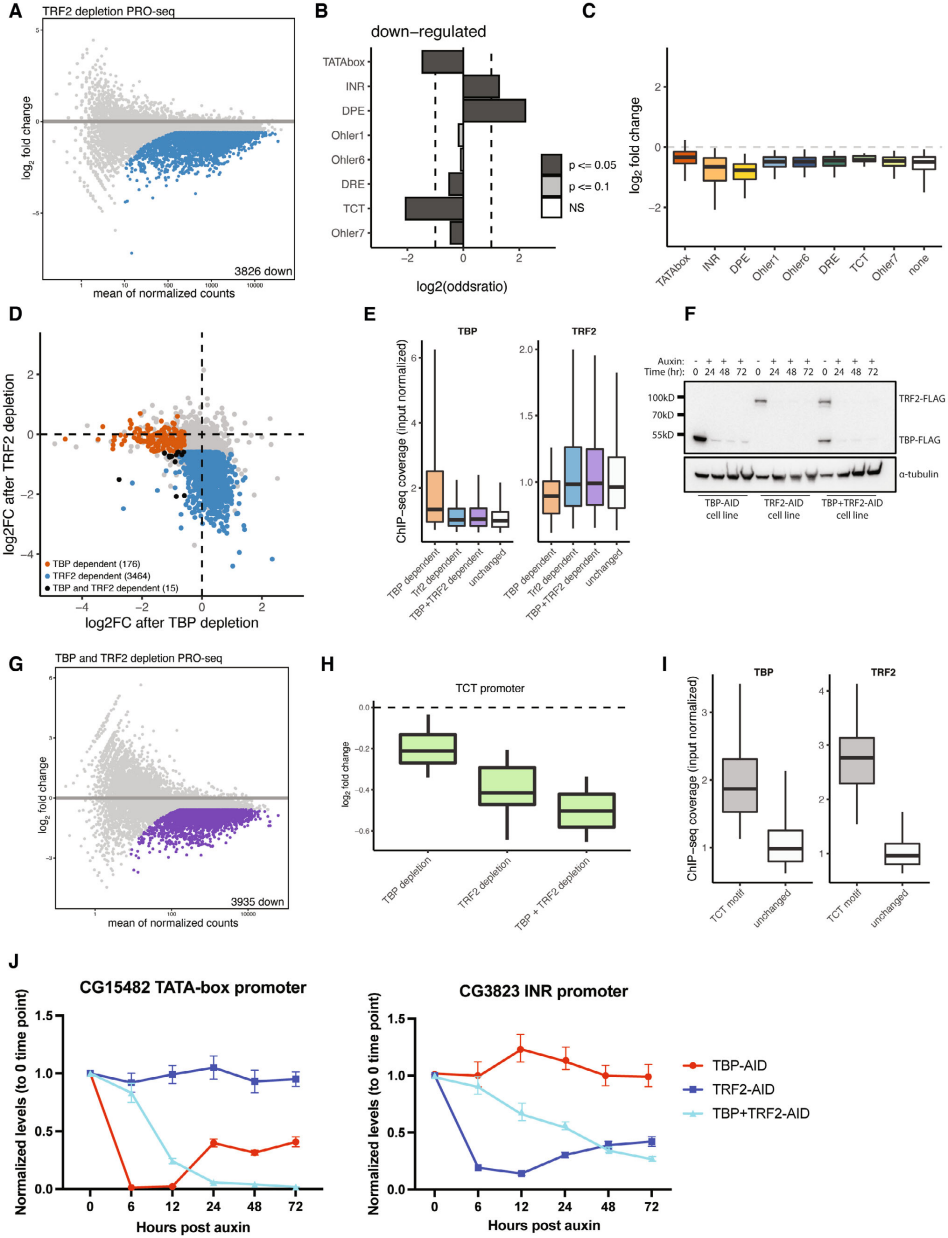
TBP and TRF2 regulate distinct subsets of developmental promoters PRO‐seq was performed upon a 6‐h auxin treatment of Trf2 depletion. Colored dots represent significantly downregulated genes (fold change > 1.5 down; FDR < 0.05).Motif enrichment analysis of gene promoters downregulated upon Trf2 depletion using a Fischer test, log2 of the odds ratio is displayed. Dashed lines are set at a value of 1 and −1.Box plot representation of Trf2‐depletion PRO‐seq data across promoters that contain different core promoter motifs. Most developmental and housekeeping promoter types are affected with TATA‐box promoters being least affected. Boxes represent the upper and lower quartiles, with the middle band at the median. The whiskers represent the upper and lower 5^th^ percentiles. Dashed line indicates 0.Scatter plot of TBP and Trf2 depletion PRO‐seq at 6 h of auxin treatment. TBP‐dependent genes are colored in orange, Trf2‐dependent genes are colored in blue. Genes dependent on both TBP and Trf2 are colored in black (fold change > 1.5 down; FDR < 0.05).ChIP‐seq coverage (input normalized) of TBP and TRF2 at TBP and/or TRF2‐dependent promoters, and all other active promoters (‘unchanged’). Orange: TBP‐dependent promoters (i.e., promoters downregulated upon TBP depletion); blue: TRF2‐dependent promoters; purple: promoters downregulated when both, TBP and Trf2 are depleted (orange, blue and purple are not mutually exclusive sets of genes); white: unaffected promoters. Boxes represent the upper and lower quartiles, with the middle band at the median. The whiskers represent the upper and lower 5^th^ percentiles. Data were taken from two merged biological replicates.Western blot of anti‐FLAG antibody of TBP‐AID, Trf2‐AID and a double‐tagged TBP‐AID + Trf2‐AID cell lines from a multiday time course of auxin treatment showing prolonged depletion.PRO‐seq was performed upon a 12‐h auxin treatment of a double‐tagged TBP + Trf2 AID cell line. Colored dots represent significantly downregulated genes (fold change > 1.5 down; FDR < 0.05), left panel.PRO‐seq signal of individual TBP or Trf2 and double depletion of both across TCT promoters (*N* = 55). Boxes represent the upper and lower quartiles, with the middle band at the median. The whiskers represent the upper and lower 5^th^ percentiles across two biological replicates.ChIP‐seq coverage (input normalized) of TBP and Trf2 on TBP across TCT promoters (*N* = 55), all other expressed by not changing promoters are labeled as “unchanged.” Boxes represent the upper and lower quartiles, with the middle band at the median. The whiskers represent the upper and lower 5^th^ percentiles. Data were taken from two merged biological replicates.qPCR on an auxin treatment time course of TBP‐ and Trf2‐dependent genes upon individual depletion of TBP or Trf2 and a double depletion of both. Error bars represent the standard deviation across three biological replicates.

To determine which promoters depend on TRF2, we AID‐tagged the evolutionarily conserved short isoform of TRF2 that is expressed in S2 cells and rapidly depleted the endogenous protein by the addition of auxin. Mass spectrometric measurement of TRF2 identified peptides shared between the two isoforms, which were depleted after the addition of auxin (Fig EV4B and C). PRO‐seq after 6 h of auxin treatment resulted in the downregulation of 3,826 genes (Fig 4A), 19 times more than the 200 genes that depend on TBP (Fig 3C). The promoters of these TRF2‐dependent genes were enriched in DPE and INR motifs, while TATA‐box and TCT motifs were depleted (Fig 4B), suggesting that TBP‐ and TRF2‐dependent genes/promoters might be different. Indeed, TRF2 depletion most strongly downregulated the INR and DPE type promoters, while TATA‐box and TCT promoters were among the least affected (Fig 4C), and genes downregulated following TBP or TRF2 depletion were largely distinct (Fig 4D). Reanalysis of published ChIP‐seq datasets confirms that TBP and TRF2 localize to different promoters: TBP‐dependent promoters preferentially bound TBP but not TRF2 and, vice versa, TRF2‐dependent promoters preferentially bound TRF2 but not TBP (Fig 4E). This mutual exclusivity suggests that DPE and INR developmental promoters and housekeeping promoters, which are all TATA‐less promoters, utilize TRF2 but not TBP to assemble a Pol II PIC *in vivo* (Fig EV4L).

The depletion of TBP or TRF2 individually left approximately half of the expressed genes largely unaffected, including the TCT‐promoter‐bearing ribosomal protein genes, suggesting that TBP and TRF2 might function partially redundantly. We AID‐tagged both genes in a single cell line (Fig 4F; see Materials and Methods), which allowed the simultaneous, auxin‐inducible depletion of endogenous TBP and TRF2 (albeit with slower depletion kinetics of TBP compared with TRF2 and TBP in the TBP‐AID single‐tagged cell line; Fig EV4D). We performed PRO‐seq after 12 h of auxin treatment, which resulted in the downregulation 3,935 genes, including all three developmental promoter subtypes and also the TCT promoters (Fig 4G–I). Consistent with the downregulation of TCT promoters, the combined depletion of both TBP and TRF2 resulted in growth arrest of the auxin‐treated cells, starting between 24 and 48 h after auxin treatment (Fig EV4F). The result that TCT promoters appear to function with either TBP or TRF2, which seem to function redundantly, is consistent with strong ChIP‐seq signals for both TBP and TRF2 at these promoters (Fig 4H).

Surprisingly, prolonged individual depletion of either TBP or TRF2 resulted in partial recovery of transcription after 24 h at several tested developmental promoters; however, double depletion of both TBP and TRF2 resulted in continued downregulation of these genes (Figs 4J and EV4I). Auxin washout experiments indicated that transcription of the tested genes recovered rapidly and fully (Fig EV4H). The apparent functional redundancy between TBP and TRF2 does not seem to stem from a global compensatory response that upregulates or stabilizes TBP after TRF2 depletion as evidenced by label‐free mass spectrometry (Fig EV4E) and thus presumably stems from increased binding of TBP to promoters (not tested). These results indicate that promoters preferentially use either TBP or TRF2 but can utilize either paralog in the absence of the other.

### All promoter types—including housekeeping promoters—depend on TFIIA

**Figure 5:**
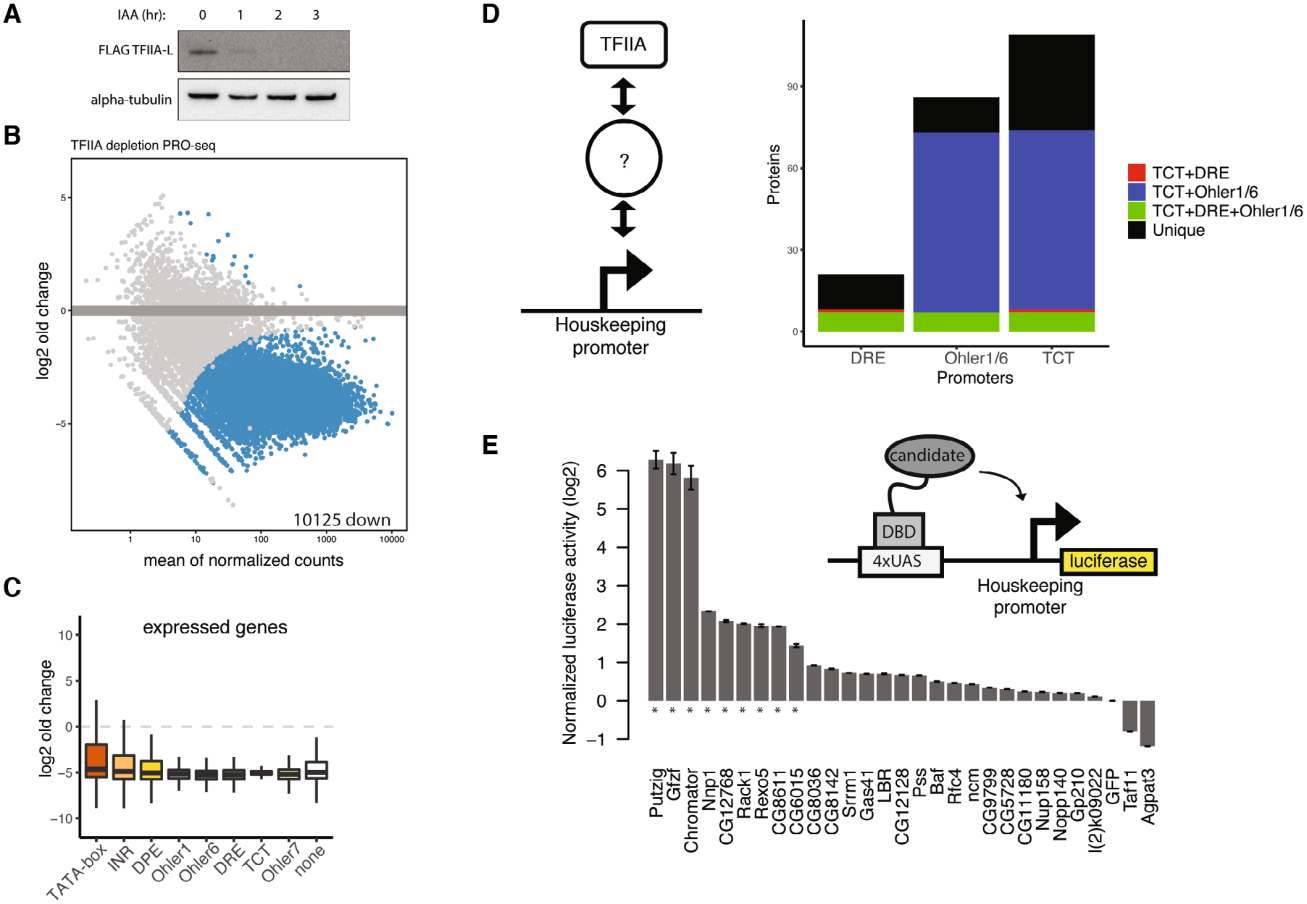
TFIIA is required by all promoters and is recruited by housekeeping cofactors to housekeeping promoters Western blot for an endogenously tagged TFIIA‐β‐3x‐FLAG‐AID cell line after addition of auxin at 1, 2 and 3 h indicating the TFIIA‐β C‐terminal cleaved product.MA‐plot of PRO‐seq measurement 6 h after auxin addition to the TFIIA‐β‐AID cell line. Colored dots represent significant downregulation (fold change > 1.5 down; FDR < 0.05). Two biological replicates per condition. 10,125 protein‐coding genes are downregulated, with 73 genes not showing downregulation due to their overlap with noncoding RNA genes such as tRNA which are not affected by TFIIA‐β depletion.PRO‐seq signal at all expressed promoters, represented according to their motif content in box plots. Boxes represent the upper and lower quartiles, with the middle band at the median. The whiskers represent the upper and lower 5^th^ percentiles across two biological replicates.Overlap of TFIIA‐β‐3xFLAG immunoprecipitation mass spectrometry data with DNA affinity purification mass spectrometry of the three tested housekeeping promoter types. Three biological replicates per conditions with Limma *P*‐value < 0.05 and enrichment > 0.Luciferase assay in which Gal4 DNA‐binding domain fusion proteins were recruited to 4xUAS sites upstream of a minimal housekeeping Rps12 promoter. Measurements are normalized to Renilla luciferase (transfection control) and GFP. * denotes proteins activating a housekeeping promoter with a log2FC > 1.5 and *P*‐value < 0.05, two‐tailed student's *t*‐test. Error bars represent standard deviation across four biological replicates.

**Figure EV5:**
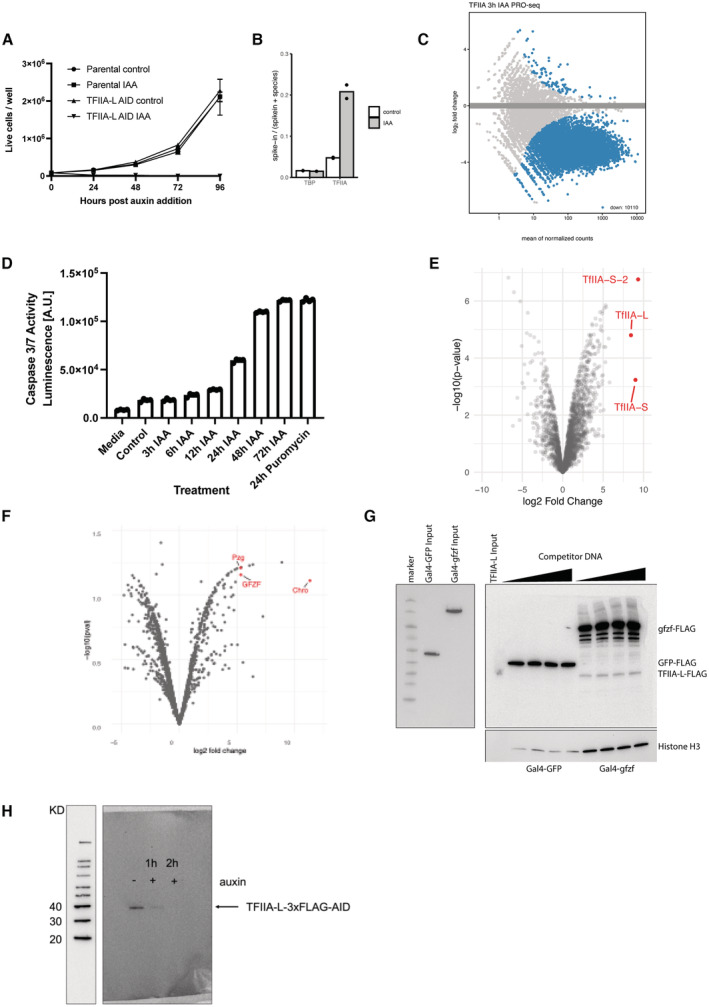
TFIIA is required by all promoters and is recruited by housekeeping cofactors to housekeeping promoters Growth curve of TFIIA‐L‐3xFLAG‐AID cell line and parental Tir1 expressing control over 4 days upon the addition of 500 μM auxin. TFIIA‐L‐AID treated cells die after 24 h. Error bars represent standard deviation across three biological replicates.Fraction of reads mapping to the D. mel genome (reference species) and the human genome (spike‐in) in PRO‐seq experiments depleting TBP or TFIIA‐L. A ~ 4‐fold increase in proportion of reads mapping to the spike‐in genome is observed only upon depletion of TFIIA‐L due to global failure of Pol II transcription in the TFIIA‐L‐AID cell line.MA‐plot of PRO‐seq data in the TFIIA‐L‐AID cell line after 3 h of auxin treatment, showing a global failure of Pol II transcription.Caspase 3 and 7 activity was measured with the Promega Caspase 3/7 Glo kit of TFIIA‐L‐AID cells after addition of auxin at various time points. A positive cell death control was included as a 24 h treatment of 10 μg/ml puromycin.Volcano plot of TFIIA‐L immunoprecipitation mass spectrometry. TFIIA‐L was immunoprecipitated from the endogenously tagged TFIIA‐L‐3xFLAG‐AID cell line using anti‐FLAG beads. Enrichment was measured over control immunoprecipitation made from the Tir1 expressing parental cell line which does not contain any FLAG epitope. Three replicates were performed for each condition.Volcano plot of Chromator immunoprecipitation mass spectrometry. Chromator was immunoprecipitated from the Chromator‐3xFLAG‐AID cell line using an anti‐FLAG antibody. Similar Tir1 expressing parental cell line control was used to measure enrichment. Putzig (Pzg) and GFZF are also highlighted.DNA affinity purification assay was performed with a 121‐bp‐long housekeeping DRE promoter with 4xUAS sites upstream. Initially, a nuclear extract containing a Gal4‐DNA‐binding domain fusion of GFP or GFZF was incubated with the bead‐immobilized promoter DNA (left panel). After the incubation, the extract was removed, and the beads were used for a DNA affinity purification assay with a nuclear extract containing TFIIA‐L‐AID‐3xFLAG as described in the materials and methods. Sheared salmon sperm DNA was used as competitor DNA at 600 ng to 1.6 μg per reaction. Elution fractions were run on an SDS–PAGE gel and blotted with a FLAG antibody (right panel).Western blot against FLAG antibody visualizing whole cell lysate from a TFIIA‐L c‐terminally tagged 3x‐FLAG‐AID line treated with auxin for 2 h. Full degradation of the TFIIA‐L beta subunit is visible upon 2 h of auxin treatment.

Our data suggest that the canonical PIC, including TFIIA, forms a closer physical association with developmental promoters when compared to housekeeping promoters. To test the functional dependency of different promoter subtypes on TFIIA, we tagged TFIIA with AID (other GTFs such as TFIIE (α and β subunit), TFIIF (α and β subunit), and TFIIB were incompatible with tagging at either the N‐ or C‐termini and could therefore not be assessed). Given the proteolytic processing of the TFIIA‐L precursor protein by Taspase A to generate TFIIA‐β (Yokomori *et al*, 1993; Zhou *et al*, 2006), we endogenously tagged TFIIA‐L at its C terminus, which was retained in TFIIA‐β, and hereafter refer to the tagged protein as TFIIA‐β and TFIIA‐AID for simplicity (Fig 5A). Auxin treatment efficiently depleted TFIIA‐AID within 1–2 h, resulting in loss of PRO‐seq signal for essentially all expressed protein‐coding genes in S2 cells within 3 and 6 h, and cell death after 24 h (Figs 5A–C and EV5A–D). These results suggest that TFIIA is functionally required at all promoter types, including housekeeping promoters. As housekeeping promoter DNA recruits TFIIA only weakly (see above), TFIIA might be recruited to housekeeping promoters via a novel mechanism, independently of DNA‐mediated recruitment of TBP.

### Intermediary proteins recruit TFIIA to housekeeping promoters

As housekeeping promoters depend on TFIIA for transcription in cells but fail to enrich for TFIIA by DNA affinity purification *in vitro*, we hypothesized that intermediary proteins interact with both, the housekeeping promoter DNA and TFIIA to mediate PIC assembly (Fig 5D). We thus performed immunoprecipitation mass spectrometry with the endogenously tagged TFIIA‐L‐AID‐3xFLAG S2 cell line and the parental Tir1‐expressing cell line as a control. We uncovered 300 TFIIA interacting proteins, including all three known components of the TFIIA complex and other TFIIA interactors, such as the TBP paralog TRF2 (but not TBP), members of the TFIID complex, and various GTFs, such as TFIIE (Fig EV5E; Dataset EV2).

To identify candidate intermediary proteins, we intersected the TFIIA binding proteins with the proteins enriched on housekeeping promoters *in vitro* (Fig 5D). Applying this strategy to developmental promoters as a positive control identified the most known GTFs, thus validating the approach. We found 131 proteins that can associate with TFIIA and at least one housekeeping promoter subtype (Fig 5D), including DREF, Chromator, GFZF, Putzig, the nucleolar protein Nnp1, and the RNA helicase CG8611 (Dataset EV2).

To determine whether the candidate TFIIA‐recruiting proteins can activate transcription from a housekeeping promoter, we fused 28 candidate proteins to the Gal4 DNA‐binding domain and tethered them to a UAS sequence upstream of a minimal housekeeping core promoter driving luciferase in S2 cells (Fig 5E). We found that nine proteins were able to transactivate the housekeeping promoter (fold change > 4 & *P* < 0.05), particularly the coactivators GFZF, Putzig, and Chromator (Fig 5E), suggesting that they may mediate TFIIA recruitment. The top three activators: GFZF, Putzig, and Chromator have previously been observed to bind housekeeping promoters, and immunoprecipitation of Chromator followed by mass spectrometry indicated these three proteins strongly interact with each other (Fig EV5F). Indeed, when we performed DNA affinity purification with a UAS‐housekeeping promoter DNA fragment, we observed co‐recruitment of TFIIA with Gal4‐GFZF but not Gal4‐GFP onto promoter DNA *in vitro* (Fig EV5G). These data suggest that GFZF can recruit TFIIA and transactivate housekeeping promoters.

Overall, these results suggest that housekeeping promoters recruit TFIIA‐β and Pol II indirectly via intermediary housekeeping cofactor proteins interacting with DNA‐binding proteins, whereas developmental promoters recruit TFIIA and the PIC directly via TBP/TRF2‐DNA interactions.

### Housekeeping cofactors underlie dispersed transcription initiation patterns

The results so far suggest that housekeeping promoters are unable to directly recruit a canonical PIC *in vitro* and may exhibit weaker and more indirect interactions with GTFs. We hypothesized that a less direct promoter DNA‐TFIIA or DNA‐PIC interface at housekeeping promoters might lead to a weak alignment between TSSs and the relevant core promoter sequence elements, such as DREF or Ohler 1/6 motifs.

**Figure 6:**
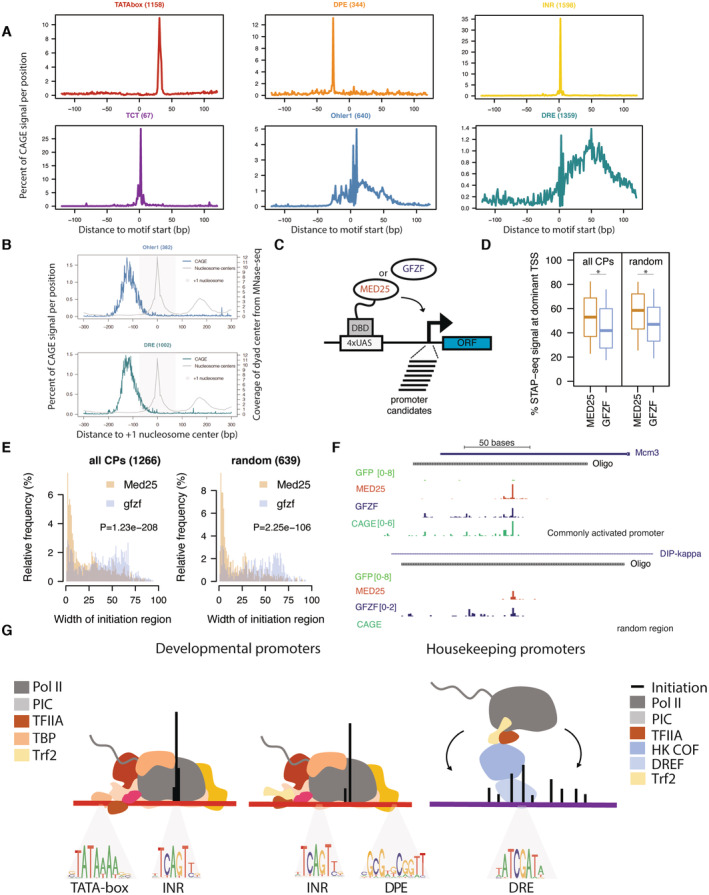
Housekeeping cofactor recruitment is sufficient to recapitulate dispersed transcription initiation patterns Distribution of CAGE signal from mixed *D. mel* embryos (0‐24 h) centered on the location of promoter DNA motif sequence set at position 0 across the 6 main promoter types investigated in this study.Relative CAGE signal per position on all active promoters containing either Ohler 1 (top) or DRE (bottom) motif, aligned to the +1 nucleosome center (point of highest coverage of MNase fragment centers in +1 to +200 bp window relative to TSS).Scheme of cofactor recruitment STAP‐seq testing MED25 or GFZF Gal4 DNA‐binding domain fusions recruited to a library of candidate promoter fragments.Box plot of the percent of STAP‐seq signal (i.e., percent of initiation) originating at the dominant TSS at core promoters (CPs; *N* = 1,266) and random regions (*N* = 639) that are activated to similar extent by both GFZF and MED25 recruitment. Cofactor recruitment STAP‐seq data from (Haberle *et al*, 2019), three independent biological replicates merged. *P ≤ 0.01; Wilcoxon rank‐sum test.Histogram representing the distribution of the width of the initiation region (i.e., part of the oligo covered by STAP‐seq signal) for CPs (*N* = 1,266) and random regions (*N* = 639) upon recruitment of either MED25 or GFZF. *P*‐values: Wilcoxon rank‐sum test.Cofactor recruitment STAP‐seq tracks of GFP, MED25 and GFZF recruitment for examples of a core promoter and a random region that are activated by both cofactors. Endogenous initiation pattern in S2 cells (CAGE) is shown at the bottom.Scheme of Pol II PIC recruitment to the two types of developmental promoters (TATA‐box and non‐TATA‐box‐containing DPE and INR motifs), which occurs through direct engagement between the transcription machinery and developmental promoter sequence motifs, resulting in narrow initiation patterns, whereas housekeeping promoters recruit Pol II through housekeeping DNA‐binding proteins and intermediary cofactors that interact with TFIIA and Trf2, resulting in dispersed initiation.

To test this hypothesis, we used Cap Analysis of Gene Expression (CAGE) data to analyze the distribution of TSSs relative to the positions of various motifs across *D. melanogaster* promoters. As expected (e.g., Ohler *et al*, 2002; Parry *et al*, 2010; Rach *et al*, 2011) the TSSs of developmental promoters, such as TATA‐box‐, INR‐ or DPE‐containing promoters, were restricted to a narrow window at consistent and precise distances from the core promoter sequence elements (Fig 6A). Similarly, the TCT‐type housekeeping promoters exhibit a focused initiation pattern precisely at the TCT motif (Wang *et al*, 2014). These results confirm that initiation is precisely aligned to the TATA‐box, INR, DPE, and TCT motifs, as expected given previous reports and the fact that these motifs direct PIC and Pol II recruitment and initiation through TBP or TRF2 (Sawadogo & Roeder, 1985; Rach *et al*, 2011).

**Figure EV6:**
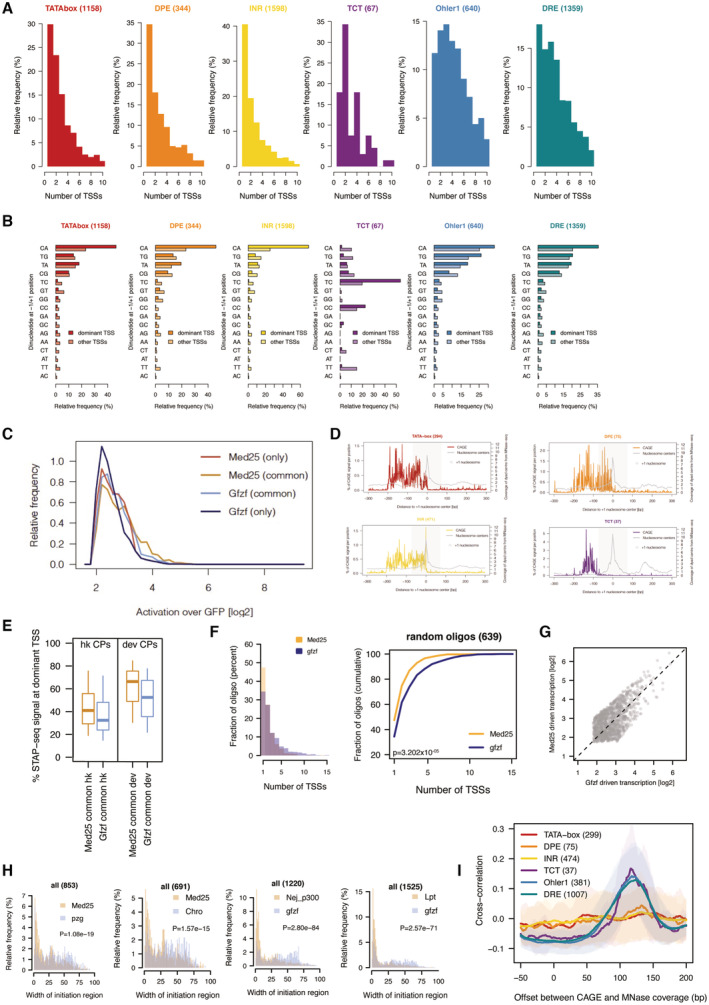
Housekeeping cofactor recruitment is sufficient to recapitulate dispersed transcription initiation patterns The number of CAGE‐defined TSSs in each promoter type over a 120 ± bp region. TSS was defined as a position having at least 20% CAGE signal as the dominant TSS in the tested region.Frequency of dinucleotides at the −1/+1 position for the dominant and secondary TSSs in each promoter type in a 120 ± bp window.Fold change (log2) of STAP‐seq signal upon GFZF or MED25 recruitment over GFP for oligos that are matched for their activation level by either one of both cofactors.Relative CAGE signal per position on all active promoters of the indicated type aligned to the +1 nucleosome center (point of highest coverage of MNase fragment centers in +1 to +200 bp window relative to TSS).Percent of STAP‐seq signal at the dominant TSS for activation matched oligos (one activated oligo per gene TSS) for housekeeping and developmental promoters that can be activated by both MED25 and GFZF. Boxes represent the upper and lower quartiles, with the middle band at the median. The whiskers represent the upper and lower 5^th^ percentiles across three biological replicates.Histogram showing the number of TSSs activated upon GFZF or MED25 recruitment on random regions that are responsive to both cofactors (left). Cumulative plot of the same data (right). *P*‐values: Kolmogorov–Smirnov test.Scatter plot of the log2 fold change above GFP (i.e., activation) of promoters by GFZF or MED25 used in the analysis (i.e., matched to be activated to similar extent by both cofactors).Histogram representing distribution of the width of the initiation region (i.e., part of the oligo covered by STAP‐seq signal) upon recruitment of MED25 or Putzig (Pzg), Med25 or Chro (Chromator), p300 or GFZF, Lpt or GFZF. For each comparison core promoters activated to similar extent by both analyzed cofactors were included. *P*‐values: Wilcoxon rank‐sum test.Cross‐correlation analysis between CAGE and MNase‐seq reads relative to the dominant CAGE TSS at developmental (TATA‐box, DPE, INR) and housekeeping (TCT, Ohler1, DRE) promoters. The mean (line) and standard deviation (shaded area) for the cross‐correlation are plotted at different offsets in a base‐pair window of −50 to 200 in relation to the dominant TSS.

In contrast, DRE‐ and Ohler 1‐containing housekeeping promoters showed a dispersed distribution of CAGE signal in relation to DRE and Ohler 1 motifs, even for promoters that contain only a single motif occurrence (Figs 6A and EV6A and B). Therefore, even though these motifs directly bind the DREF and M1BP factors, which can in turn recruit TFIIA, they do not instruct TSS position. We propose that the lack of strict motif positioning and initiation site at these housekeeping promoters is a direct result of weaker and less defined DNA‐PIC interactions.

As transcription initiation at housekeeping promoters was not aligned to a sequence feature, we considered whether the promoter‐proximal chromatin structure, especially the nucleosome‐depleted region (NDR) or the +1 nucleosome might constrain initiation patterns. Although the CAGE signal is not strongly aligned with the +1 nucleosome at developmental promoters, housekeeping promoters exhibit a broad distribution of CAGE signal in the NDR immediately upstream of a strongly positioned +1 nucleosome (Figs 6B and EV6D). These data show that initiation at housekeeping promoters occurs in a rather broad NDR upstream of the +1 nucleosome and suggest that the chromatin structure might be involved in determining TSS positions as previously proposed (Field *et al*, 2008; Rach *et al*, 2011; Ho *et al*, 2014). Cross‐correlation analysis of CAGE and MNase‐seq data further confirms a peak in cross‐correlation between both datasets 125 bp downstream of TSSs for housekeeping promoters (TCT, Ohler 1, and DRE) but not developmental promoters (TATA‐box, DPE, and INR), suggesting a preferred +1 nucleosome position downstream of dominant housekeeping TSSs (Fig EV6I). Consistently, when +1 nucleosome centers according to MNase‐seq were aligned to the dominant TSSs, developmental promoters did not exhibit preferred nucleosome positions, while housekeeping promoters exhibited a clear preferred position downstream of the TSS (Appendix Fig S2). Overall, these analyses suggest that the +1 nucleosome assumes a more stereotypical position relative to the dominant TSS in housekeeping promoters compared with developmental promoters, suggesting that chromatin and nucleosome positioning might have a more instructive role for TSS positions in housekeeping promoters.

If the dispersed initiation at housekeeping promoters results from a different mechanism of Pol II PIC recruitment, then transcriptional activation from the housekeeping‐type TFIIA recruitment factors GFZF, Putzig, and Chromator described above should always lead to more dispersed TSS patterns, irrespective of the promoter sequence. To test this systematically, we recruited the developmental‐type coactivator MED25 and the housekeeping‐type coactivator GFZF to a library of candidate promoters and analyzed the transcription initiation patterns (data from Haberle *et al*, 2019; Fig 6C). Although the two coactivators preferentially activate distinct sets of promoters (Haberle *et al*, 2019), 1,266 promoters and 1,268 random control sequences were activated sufficiently strongly by both coactivators to compare the respective initiation patterns (> fourfold induction over GFP with FDR < 0.05; Fig EV6C and G).

To systematically assess the initiation patterns across these fragments, we calculated the proportion of initiation events at the dominant TSS compared with the sum of all initiation events across the entire promoter fragment. On average, across all core promoter fragments, initiation was at the dominant TSS for 55% of events after MED25 recruitment but only 42% after GFZF recruitment (*P* = 1.6 × 10^−28^; Wilcoxon rank‐sum test, Fig 6D). This difference persisted when housekeeping and developmental promoter sequences were analyzed separately (Fig EV6E) and even for random nonpromoter fragments, for which the corresponding proportions were 59 versus 49% (*P* = 2.4 × 10^−22^; Fig 6D).

Consistently, when we examined all substantially activated TSSs within the nonpromoter fragments (Fig EV6G), we found a single TSS for 47% of the fragments upon MED25 recruitment, while only 7% had 5 or more TSSs. In contrast, GFZF recruitment led to a single TSS for only 34% of the fragments, while 17% had 5 or more TSSs (Fig EV6F). Moreover, MED25‐induced transcription initiated for most promoters (51%) within a narrow 20 bp region, while GFZF‐induced transcription generally initiated in a much broader region of 30 to 75 bp (only 24% promoters initiated within 20 bp; Fig 6E). The existence of distinct initiation patterns for the same DNA sequence after MED25 versus GFZP recruitment is illustrated by the promoter of the Mcm3 gene and an intronic sequence within the DIP‐kappa gene that does not initiate transcription endogenously (Fig 6F). The activation of transcription in characteristically different initiation patterns was also observed for two additional developmental (p300 and Lpt) and two housekeeping cofactors (Putzig and Chromator), respectively (Fig EV6H).

Thus, cofactor recruitment under identical conditions in an identical sequence context led to initiation patterns that are characteristically different for developmental cofactors (e.g., MED25) and housekeeping cofactors (e.g., GFZF), suggesting coactivators impose distinct initiation patterns due to their different mechanisms of recruiting TFIIA, and the Pol II PIC.

## Discussion

In contrast to a prevalent model that Pol II PIC assembly and transcription activation occur similarly at all promoters, we find that different core promoter types recruit and activate Pol II via distinct strategies that depend on different factors.

Developmental promoter DNA is sufficient to recruit and assemble a Pol II PIC from nuclear extract *in vitro*, by having high affinity to GTFs such as TBP. Found as part of a soluble Pol II holoenzyme in yeast, TBP in complex with TFIIA is tightly associated with chromatin in metazoans and important in directing Pol II PIC assembly on DNA and cofactor mediated transcription *in vitro* (Koleske & Young, 1995; Lieberman *et al*, 1997; Kimura *et al*, 1999).

Our data indicate that most TATA‐less promoters are independent of TBP and utilize TRF2, or TBP and TRF2 in a redundant fashion. Transcription in the absence of TBP has been observed for particular promoters (Wieczorek *et al*, 1998; Kwan *et al*, 2021) and cell types (Martianov *et al*, 2002; Gazdag *et al*, 2016), potentially involving TBP paralogs such as TRF2 in flies. Even though TRF2 has been reported to be unable to bind DNA directly (Rabenstein *et al*, 1999; Baumann *et al*, 2018), it may be recruited indirectly to promoters, potentially through interactions with TFIIA and/or TFIID (Baumann & Gilmour, 2017). This is analogous to transcription initiation during oocyte growth when the mammalian TBP paralog TBPL2 cooperates with TFIIA to initiate transcription independently of TFIID (Yu *et al*, 2020). The promoters of snRNA genes also function independently of TBP yet depend on SNAPc. At these promoters, SNAPc seems to directly bind TFIIA and/or TFIIB via an interface shared with TBP (Mittal *et al*, 1999; Dergai *et al*, 2018; Rengachari *et al*, 2022).

The partial redundancy of TBP and TRF2, especially when one of the two is depleted reconciles our results with recent structural studies of PIC assembly at non‐TATA‐box promoters (Chen *et al*, 2021): as TBPL1 or other TBP paralogs had not been considered during complex assembly *in vitro*, TBP was included in the PIC, irrespective of the promoter type. This might have been possible given the flexibility of the PIC, including TFIID that has been reported as sufficiently flexible to accommodate either TBP or TRF2 at different classes of promoters (Louder *et al*, 2016).

Interestingly, we find several proteins that had been described as insulator or architectural proteins bound to housekeeping promoters, both *in vitro* and *in vivo*. This is consistent with the observations that topological chromatin boundaries in *Drosophila* coincide with housekeeping genes (Cubeñas‐Potts *et al*, 2017). This could either be a coincidence or—more likely—reflect that these genomic regions and proteins mediate both functions. At least Chromator has transcription‐activating activity toward housekeeping core promoters (Stampfel *et al*, 2015; Haberle *et al*, 2019; Fig 5E). It is interesting to speculate whether the housekeeping transcriptional program, which is inherently incompatible with cell‐type‐specific or developmental transcriptional regulation (Zabidi *et al*, 2015; Haberle *et al*, 2019), can per se mediate insulation or if the respective factors have evolved both functions independently.

Housekeeping promoters also bind sequence‐specific TFs such as DREF and M1BP, which in turn interact with cofactors such as GFZF, Chromator and Putzig that—directly or indirectly—recruit GTFs (e.g., TFIIA) and Pol II (Hochheimer *et al*, 2002; Baumann *et al*, 2018). These differences in the assembly and stability of the DNA–protein interface and protein complexes might explain the distinct transcription initiation patterns at developmental and housekeeping promoters (Fig 6G), which generally exhibit focused and dispersed initiation patterns, respectively. Indeed, forced recruitment of housekeeping activators such as GFZF to arbitrary DNA sequences is sufficient to induce broad transcription initiation patterns, consistent with the initiation patterns observed at housekeeping promoters *in vivo* and with alternative PIC recruitment. This directly links the transcription‐activating cofactors of developmental and housekeeping programs to the distinct initiation patterns observed for the respective promoters. We note that even for dispersed housekeeping promoters, TSS choice is not entirely random or arbitrary but that certain positions seem to be favored, likely relating to local DNA structure, the energy barrier landscape for both DNA helix melting and phospho‐diester‐bond formation (e.g., Dineen *et al*, 2009; Haberle *et al*, 2019).

Given that key features of the promoter types, such as their initiation patterns, sequence motifs and their enhancer responsiveness is observed in *Drosophila* cell types as different as embryonic S2 cells and adult ovarian OSCs (Arnold *et al*, 2016), and because GTFs are typically broadly expressed across cell types (Haberle & Stark, 2018), we expect the relative utilization of cofactors to be similar in most cellular contexts. Moreover, while some of the specific TFs do not have one‐to‐one orthologs outside insects, focused and dispersed initiation patterns are widely observed across a wide range of species, including mammals. It will be exciting to see how homologous and analogous factors function at these distinct promoter types in different species.

The alternative mechanisms converge on TFIIA that is essential for transcription initiation at all promoter types. A central role of TFIIA recruitment for transcription initiation is consistent with the direct interaction of the TBP paralog TBPL2 with TFIIA in oocyte transcription (Yu *et al*, 2020), the direct interaction of SNAPc with TFIIA and/or TFIIB (Dergai *et al*, 2018; Rengachari *et al*, 2022) and noncanonical Pol II transcription of transposon‐rich and H3K9me3‐marked piRNA source loci in *Drosophila* germ cells through the TFIIA paralog moonshiner and TRF2 (Andersen *et al*, 2017). Essentiality for some or all promoter types might extend to other GTFs that we could not test here, including TFIIB that is required at most promoters in human HAP1 cells (Santana *et al*, 2022).

Some features of *Drosophila* housekeeping promoters, including the dispersed patterns of transcription initiation, are similarly observed for the majority of vertebrate CpG island promoters comprising roughly 70% of all promoters (Carninci *et al*, 2006; Saxonov *et al*, 2006; FANTOM Consortium and the RIKEN PMI and CLST (DGT) *et al*, 2014; Danks *et al*, 2018). The functional regulatory dichotomy of these promoters combined with the evidence of distinct PIC composition and initiation mechanisms here and in other recent studies (Haberle *et al*, 2019; Baek *et al*, 2021) suggest that we need to challenge the notion of a universal model of rigid and uniform PIC assembly. It will be exciting to see future functional, biochemical, and structural studies revealing more diverse transcription initiation mechanisms at the different promoter types in our genomes.

## Materials and Methods

### Reagents and tools table

**Table:** 

Reagent/Resource	Reference or Source	Identifier or Catalog Number
**Experimental Models**		
*D. melanogaster* Schneider S2 cells	Thermo Fisher	Cat#R69007
HCT116	ATCC	Cat#CCL‐247
Parental OsTir expressing S2 cell line	This study	N/A
TRF2 C‐terminally tagged AID S2 cell line	This study	N/A
TBP N‐terminally tagged AID S2 cell line	This study	N/A
DREF N‐terminally tagged AID S2 cell line	This study	N/A
TFIIA C‐terminally tagged AID S2 cell line	This study	N/A
Chromator N‐terminally tagged AID S2 cell line	This study	N/A
**Recombinant DNA**		
pBabe Puro osTIR1‐9Myc	Addgene	plasmid #80074
pAc‐sgRNA‐Cas9	Addgene	plasmid #49330
pCRIS‐PITChv2‐FBL	Addgene	plasmid #63672
pGL13_tGFP	This study	N/A
**Antibodies**		
Mouse monoclonal anti‐FLAG	Sigma‐Aldrich	Cat#F3165
Secondary anti‐mouse HRP	Sigma‐Aldrich	Cat#12‐349
Histone H3	Abcam	Cat#ab1791
Alpha‐tubulin	Abcam	Cat#Ab18251
Secondary anti‐rabbit HRP	Sigma‐Aldrich	Cat#12‐348
**Oligonucleotides and other sequence‐based reagents** *For long lists of oligos or other sequences please refer to the relevant Table(s) or EV Table(s)*		
5′‐ /5Phos/rNrNrN rNrNrN rNrNrG rArUrC rGrUrC rGrGrA rCrUrG rUrArG rArArC rUrCrU rGrArA rC/3InvdT/‐3′ (3′ RNA linker)	IDT	N/A
5‐rCrCrU rUrGrG rCrArC rCrCrG rArGrA rArUrU rCrCrA rNrNrN rN ‐3 (5′ RNA linker)	IDT	N/A
Biotin TEG 5′ [BtnTg]GCAGGTGCCAGAACATTTCTCTATCGATAGG	Sigma‐Aldrich	N/A
Reverse 3′ CTTTACCAACAGTACCGGAATGC	Sigma‐Aldrich	N/A
Act5C gRNA forward TTCGGACCGCAAGTGCTTCTAAGA	Sigma‐Aldrich	N/A
Act5C gRNA reverse AACTCTTAGAAGCACTTGCGGTC	Sigma‐Aldrich	N/A
TBP N‐terminus gRNA forward TTCGACAATAAACCATCTGTAAGA	Sigma‐Aldrich	N/A
TBP N‐terminus gRNA reverse AACTCTTACAGATGGTTTATTGTC	Sigma‐Aldrich	N/A
DREF N‐terminus gRNA forward ttcGGAAGACAAGATGAGCGAAG	Sigma‐Aldrich	N/A
DREF N‐terminus gRNA reverse aacCTTCGCTCATCTTGTCTTCC	Sigma‐Aldrich	N/A
Chromator N‐terminus gRNA forward TTCGCTGGAGTCGTGAATAATGT	Sigma‐Aldrich	N/A
Chromator N‐terminus gRNA reverse AACACATTATTCACGACTCCAGC	Sigma‐Aldrich	N/A
TFIIA‐L C‐terminus gRNA forward TTCGCGACGCCGAGTGGTAATGGA	Sigma‐Aldrich	N/A
TFIIA‐L C‐terminus gRNA reverse AACTCCATTACCACTCGGCGTCGC	Sigma‐Aldrich	N/A
TBP AID N‐terminal repair cassette forward CCGCGTTACATAGCATCGTACGCGTACGTGTTTGGTCCACAATAAACCATCTGTAATGGCCAAGCCTTTGTCTCAAG	Sigma‐Aldrich	N/A
TBP AID N‐terminal repair cassette reverse CATCAGCATTCTAGAGCATCGTACGCGTACGTGTTTGGCTTAGCATTTGGTCCATCTGCGAGCCACCGCCCGATC	Sigma‐Aldrich	N/A
DREF AID N‐terminal repair cassette forward ccgcgttacatagcatcgtacgcgtacgtgtttggCACAGAAGACAAGATGAGCGATGGCCAAGCCTTTGTCTCAAG	Sigma‐Aldrich	N/A
DREF AID N‐terminal repair cassette reverse catcagcattctagagcatcgtacgcgtacgtgtttggGGGCGACGCTGGTACCCCTTCCGAGCCACCGCCCGATC	Sigma‐Aldrich	N/A
TFIIA‐L AID C‐terminal repair cassette forward CCGCGTTACATAGCATCGTACGCGTACGTGTTTGGCGAATGGCGACGCCGAGTGGGGCGGTGGCTCGGGAG	Sigma‐Aldrich	N/A
TFIIA‐L AID C‐terminal repair cassette reverse CATCAGCATTCTAGAGCATCGTACGCGTACGTGTTTGGTGTTCGCTCAACTGCCATCCTTAGCCCTCCCACACATAACCAG	Sigma‐Aldrich	N/A
Chromator AID N‐terminal repair cassette forward gttccgcgttacatagcatcgtacgcgtacgtgtttggGGCGCTGGAGTCGTGAATAAATGGCCAAGCCTTTGTCTCA	Sigma‐Aldrich	N/A
Chromator AID N‐terminal repair cassette reverse catcagcattctagagcatcgtacgcgtacgtgtttggTGAAATCTCCTGTGCCAACATCGAGCCACCGCCCGATC	Sigma‐Aldrich	N/A
OsTir ligase donor cassette forward TGGATCTCCAAGCAGGAGTACGACGAGTCCGGCCCCTCCATTGTGCACCGCAAGTGCTTCGGCAGCGGCGCCAC	Sigma‐Aldrich	N/A
OsTir ligase donor cassette reverse CCTCCAGCAGAATCAAGACCATCCCGATCCTGATCCTCTTGCCCAGACAAGCGATCCTTCCTAGCCCTCCCACACATAACCAG	Sigma‐Aldrich	N/A
Genotyping Act5C OsTir forward GGCTTCGCTGTCCACCTTCCAG	Sigma‐Aldrich	N/A
Genotyping Act5C OsTir reverse GAAGTCGAGGAAGCAGCAGCGA	Sigma‐Aldrich	N/A
**Chemicals, enzymes, and other reagents** (*e.g*., *drugs*, *peptides*, *recombinant proteins*, *and dyes*)		
4–20% Mini‐PROTEAN® TGX™ Precast Protein Gels, 15‐well, 15 μl	Bio‐Rad	Cat#34561096
MegaX DH10B T1^R^ Electrocomp™ Cells	Thermo Fisher	Cat#C640003
FastDigest MluI	Thermo Fisher	Cat#FD0564
BspQI	NEB	Cat#R0712S
Blasticidin S HCl	Thermo Fisher	Cat#R21001
3‐Indoleacetic acid	Merck	Cat#I3750
QuickExtract™ DNA Extraction Solution	Lucigen	Cat#QE9059
2× Laemmli Sample Buffer	Bio‐Rad	Cat#1610737
EGTA	Merck	Cat#E4378
Biotin‐11‐CTP	PerkinElmer	Cat#NEL542001EA
Biotin‐11‐UTP	PerkinElmer	Cat#NEL543001EA
Q5 polymerase high‐fidelity 2× master mix	NEB	Cat#M0492S
Trizol	Thermo Fisher	Cat#15596026
Trizol‐LS	Thermo Fisher	Cat#10296010
GlycoBlue™ Coprecipitant	Thermo Fisher	Cat#AM9515
NTP Set, 100 mM Solution	Thermo Fisher	Cat#R0481
N‐Lauroylsarcosine sodium salt	Merck	Cat#L5125
Dynabeads™ M‐280 Streptavidin	Thermo Fisher	Cat#11205D
Cap‐CLIP	BioZym	Cat#C‐CC15011H
T4 Polynucleotide Kinase	NEB	Cat#M0201S
Murine RNAse Inhibitor	NEB	Cat#M0314L
T4 RNA Ligase	NEB	Cat#M0204L
SuperScript™ III Reverse Transcriptase	Thermo Fisher	Cat#18080093
KAPA HiFi HotStart Real‐Time Library Amp Kit	Roche	Cat#7959028001
AMPure XP beads	Beckman Coulter	Cat#A63882
Anti‐FLAG® M2 Magnetic Beads	Merck	Cat#M8823
Lysyl endopeptidase	Wako Chemicals	Cat#7041
Ammoniumbicarbonate	Sigma‐Aldrich	Cat#09830
Tris‐(2‐carboxyethyl)‐phosphin‐hydrochloride (TCEP)	Sigma‐Aldrich	Cat#646547
S‐Methyl‐thiomethanesulfonate (MMTS)	Sigma‐Aldrich	Cat#64306
Trifluoroacetic acid	Sigma‐Aldrich	Cat#T6508
oComplete mini protease inhibitors	Sigma‐Aldrich	Cat# 11836170001
Axygen 1.5 ml MaxyClear tube	Corning	Cat#MCT‐150‐A
Axygen 0.6 ml MaxyClear tube	Corning	Cat#MCT‐060‐C‐S
Direct‐zol RNA Microprep	Zymo	Cat#R2061
Micro Bio‐spin P‐30 gel columns	Bio‐rad	7326251
**Software** *Include version where applicable*		
MSAmanda	N/A	https://ms.imp.ac.at/?goto=msamanda
Benchling	N/A	https://benchling.com
R version 3.5.3	R Development Core Team, 2019	https://www.r‐project.org
Cutadapt	Martin (2011)	https://bioweb.pasteur.fr/packages/pack@cutadapt@1.18
Samtools version 1.9	Li *et al*, 2009	http://www.htslib.org/
bowtie version 1.2.2	Langmead *et al*, 2009	https://sourceforge.net/projects/bowtie‐bio/files/bowtie/1.2.2/
GenomicRanges 1.34.0	Lawrence *et al*, 2013	https://bioconductor.org/packages/release/bioc/html/GenomicRanges.html
Biostrings 2.50.2	N/A	https://bioconductor.org/packages/Biostrings
bigBedtoBed	Kent *et al*, 2010	https://github.com/ENCODE‐DCC/kentUtils/blob/master/src/utils/bigBedToBed/bigBedToBed.c
bedtools 2.27.1	Quinlan & Hall, 2010	https://github.com/arq5x/bedtools2/releases/tag/v2.30.0
DESeq2 package v.1.30.1	Love *et al*, 2014	https://bioconductor.org/packages/release/bioc/html/DESeq2.html
**Other**		
Mini‐PROTEAN Tetra Vertical Electrophoresis Cell	Bio‐Rad	Cat#1658004
Monarch Gel Extraction	NEB	Cat#T1020L
Illumina Truseq small RNA library prep kit	Illumina	Cat#RS‐200‐0012
Power Blotter Station	Thermo Fisher	Cat#PB0010
MaxCyte STX Scalable Transfection System	Maxcyte	NA

### Methods and Protocols

#### Cell culture

*Drosophila melanogaster* S2 cells were obtained from Thermo Fisher and maintained in Schneider's *Drosophila* Medium supplemented with 10% heat‐inactivated fetal bovine serum.

#### Generation of endogenously tagged AID cell lines

A parental cell line expressing the osTir ligase was created with a knock‐in approach by introducing a vector expressing a gRNA/Cas9 targeting the carboxyl terminus of the Act5C, with a P2A before the osTir‐mCherry construct, leading to constitutive expression of the osTir ligase. Wild‐type S2 cells were electroporated using the MaxCyte STX system at a density of 1 × 10^7^ cells per 100 μl and 20 μg of DNA using the preset protocols. Cells were selected with puromycin and FACS sorted based on mCherry fluorescence into individual 96‐well plates to generate individual clones which were screened by PCR and for their ability to degrade transfected AID‐tagged proteins. To generate AID cell lines, we have electroporated a knock‐in cassette to either the N‐terminal or C‐terminal of the gene of interest, a cassette containing a mAID‐3xFLAG tag. Cells were electroporated as described above. Electroporated cells were selected on 5 μg/ml blasticidin and diluted to individual 96‐well plates to generate single clones. Single clones were amplified and genotyped using a PCR to the presence of a homozygous knock‐in and confirmed with Sanger sequencing. To generate a double‐tagged TBP + TRF2 AID cell line, the TRF2 AID cell line was electroporated with a knock‐in cassette containing a TBP‐AID with a hygromycin selection marker. Cells were selected for 1 week on 5 μg/ml hygromycin, and single clones were generated as above. Single clones were additionally tested for their ability to degrade the AID‐3xFLAG‐tagged proteins on a western blot using an anti‐FLAG antibody.

#### Correcting transcription start site (TSS) annotations by CAGE

We took transcripts of all protein‐coding genes and corrected their TSSs with CAGE data from modENCODE (Brown *et al*, 2014) following a previously established protocol (Haberle *et al*, 2019). First, TSSs were corrected by CAGE signal from S2 cells downloaded from modENCODE dataset no. 5331 that lie within a window of ±250 bps. If no hit was found, CAGE signals from mixed embryos or a developmental time course from modENCODE datasets no. 5338‐5348, 5350 and 5351 were used within the same window. If the TSS was left unsupported we repeated this using a ± 500 bp window or kept the annotated TSS. We kept the longest transcript per unique TSS. We used the R packages CAGEr 1.24.0 (Haberle *et al*, 2014) and GenomicRanges 1.34.0. This resulted in a set of 17,118 unique CAGE‐corrected protein‐coding gene transcript annotations.

#### Scoring of *Drosophila* core promoter DNA with PWMS of core promoter motifs

We scored *Drosophila* core promoters with different core promoter motifs as described previously (Haberle *et al*, 2019). Briefly, we used the 17,118 unique CAGE‐corrected protein‐coding gene TSSs (see above) and scored them with PWMs for different core promoter motifs in defined windows relative to the TSS where the motifs are expected to occur (FitzGerald *et al*, 2006). The obtained table of motif scores per core promoter/gene was used for all downstream analysis.

#### Overview over core promoter motif occurrence and abundance of promoter types

To get an unbiased global overview of core promoter motif occurrence and core promoter types in the *Drosophila* genome, we clustered all promoters based on PWM scores with k‐means clustering into 9 clusters and displayed these clusters and the relative PWM scores as a heatmap (Appendix Fig S1A). This revealed the expected well‐defined promoter types such as the TATA‐box, DPE, INR, TCT, Ohler1/6, and DRE, which are characterized by a single motif or defined combinations of motifs (promoters with less specific motif signatures were classified as “other” and not considered for further analysis). The relative abundance of these different promoter types was visualized with a pie chart for all promoters and for promoters active in S2 cells (as seen in Appendix Fig S1B). To keep this overview analysis unbiased, we did not use any thresholds, nor did we require specific motifs to co‐occur or not. In fact, the heatmap visualization displays the expected motif co‐occurrence known from the literature (Ohler *et al*, 2002; FitzGerald *et al*, 2006; Arnold *et al*, 2016; Haberle *et al*, 2019), for example, TATA‐box and INR, DPE and INR, or Ohler 1 and Ohler 6 motifs.

#### Thresholding of core promoter motif matches for downstream analyses

To enable the core promoter motif‐related downstream analysis of PRO‐seq data, we thresholded the PWM motif scores. Thresholding defined motif presence/absence in a binary fashion and enabled motif enrichment analyses for groups of promoters (e.g., those downregulated according to PRO‐seq; e.g., Figs 3E and 4B) as well as the comparison of PRO‐seq data for all promoters that contained a given motif (e.g., Figs 3G and 4C). For this, we used the following PWM motif score thresholds (percent of optimal score) that took into account the different lengths and information content of the motifs: TATA‐box > 90%, INR > 95%, DPE > 98%, TCT > 95%, Ohler1 > 95%, Ohler 6 > 97%, Ohler 7 > 95%, and DRE > 98%.

#### Selection of promoters and controls for DNA affinity purification

We selected prototypical core promoters for DNA affinity purifications by taking their activity in S2 cells, stringent motif matches, and prototypical motif co‐occurrences (Ohler *et al*, 2002; Haberle *et al*, 2019) into account. Specifically, as all experiments were performed using *Drosophila* S2‐cell nuclear extract (DNA affinity purification) or S2 cells (functional analyses), we chose promoters that were active in S2 cells according to CAGE (≥ 5tpm; Brown *et al*, 2014) and were inducible in STAP‐seq (Arnold *et al*, 2016). We further applied the following stringent thresholds and rules about motif co‐occurrence (FitzGerland *et al*, 2006; Haberle *et al*, 2019): TATA‐box promoters: TATA‐box > 95% with low matches (< 90%) for DPE and MTE and housekeeping motifs, DPE promoters: DPE > 95% with low matches to TATA‐box (< 80%) and MTE and housekeeping motifs (< 90%), INR‐only promoters: INR > 95% with low matches to TATA‐box (< 80%), DPE and MTE (< 85%) and housekeeping motifs (< 90%), TCT promoters: TCT > 95% and initiation on TC, Ohler1/6 promoters: Ohler1 & Ohler 6 > 95% and low scores for TATA‐box (< 80%), INR, DPE and MTE (< 85%) DRE (< 95%), DRE promoters: DRE = 100% with low scores for Ohler 1/6 (< 85%)and developmental motifs as above.

We selected length‐matched control regions from the *Drosophila* genome, excluding regions that showed any sign of transcription in S2 cells or in any *Drosophila* developmental CAGE data or were promoters or enhancers according to genome annotations, STARR‐seq or STAP‐seq data. Selected promoters are listed in Appendix Table S1.

#### Cloning promoter constructs

Promoter regions were PCR amplified from S2 cell genomic DNA using primers containing Gibson overhangs corresponding to the BglII and HindII restriction sites on pGL3 with Q5 high‐fidelity 2× master mix (NEB). PCR products were cleaned with AMPURE beads and eluted in water. Gibson reactions were performed with a Gibson assembly master mix (NEB) according to the manufacturer's recommendations. 1 μl of Gibson reaction was electroporated into MegaX DH10B electrocompetent cells (Thermo). Single clones were picked and grown in 5 ml bacterial cultures. Minipreps were performed using a Qiagen kit, and Sanger sequencing was performed in‐house. Correct plasmid clones were used as a template for amplification of biotinylated DNA.

#### Preparation and immobilization of biotinylated DNA

Biotinylated DNA was generating using a forward primer containing a Biotin TEG group on the 5′ end obtained from Sigma‐Aldrich: Biotin TEG 5′, and a reverse Reverse 3′ primer (see resource table for primer sequences). At least 2 ml of total PCR volume (performed in 50 μl reactions) for each individual promoter sequence was amplified individually for each replicate. PCR reactions were pooled and DNA was purified using AMPURE beads and eluted in water. For each sample, 50 μl of Dyna M280 Streptavidin was used and coupled to 15 μg of cleaned biotinylated PCR product according to the manufacturer's recommendations. The beads were placed in an equivalent volume of DBB (150 mM NaCl, 50 mM Tris/HCl pH, 8.0, 10 mM MgCl_2_) and used immediately for DNA affinity purification assay.

#### Preparation of nuclear extracts

Nuclear extracts from *Drosophila* S2 cells were prepared as previously described with the following modifications (Dignam *et al*, 1983). Three billion *Drosophila* S2 cells were harvested by resuspension and washed with PBS. The cell pellet was resuspended in buffer A (10 mM HEPES pH7.9, 1.5 mM MgCl2, 10 mM KCl, 0.5 mM DTT added fresh before use, and oComplete EDTA‐free protease inhibitors) placed on ice for 10 min. Cells were spun down at 700 *g* for 5 min, supernatant removed, and cells were resuspended in 5 cell pellet volumes of buffer A supplemented with 0.5% NP‐40. Cell suspension was dounced in a Beckman 15 ml dounce with a “loose” pestle for 10 strokes to isolate nuclei. Cells were spun down at 2,000 *g* for 5 min at 4°C, supernatant containing the cytoplasmic fraction was removed, and cell pellet containing the nuclei was resuspended in three pellet volume of buffer C (0.5 M NaCl, 20 mM HEPES pH7.9, 25% glycerol, 1.5 mM MgCl2, 0.2 mM EDTA, 0.5 mM DTT added before use, oComplete EDTA‐free protease inhibitors), and placed over a 10% sucrose cushion made in buffer C, and spun down at 3,000 *g* for 5 min at 4°C. Supernatant was removed and the pellet was resuspended in buffer C, equivalent of 1 ml per 1 billion starting cells. Nuclei were dounced in a Beckman 7 ml dounce with a “tight” pestle for 20 strokes. Lysed nuclei were rotated at 4°C for 30 min and then spun down at 20,000 *g* for 10 min at 4°C. The supernatant was the soluble nuclear fraction that was dialyzed in buffer D (20 mM HEPES pH7.9, 20% glycerol, 0.1 M KCl, 0.2 mM EDTA, 0.5 mM DTT added before use, and oComplete EDTA‐free protease inhibitors) using Slide‐A‐Lyzer dialysis cassettes with a 3.5kD molecule weight cutoff for 6 h with two buffer exchanges. Protein concentration of the nuclear extract was determined with a Qubit protein assay kit according to the manufacturer's instructions. Dialyzed nuclear extract was snap frozen in liquid nitrogen and stored at −80°C until use.

#### DNA affinity purification and on‐bead digest

50 μl of DNA‐immobilized beads was mixed with 400 μg of nuclear extract and 1,200 ng sheared salmon sperm DNA in Axygen 1.5 ml tubes. Reactions were incubated at room temperature for 40 min with rotation. Beads were then magnetically pelleted, washed once with buffer DBB (supplemented with 0.5%NP‐40), and resuspended in DBB supplemented 0.75% formaldehyde for 10 min at room temperature with rotation. Beads were resuspended in 50 μl of 100 mM ammonium bicarbonate. 600 ng of Lys‐C (Wako) was added to the beads and digests were incubated at 37°C for 4 h in a thermoblock with shaking at 800 rpm. Beads were magnetically pelleted, and the supernatant was transferred to a new 0.6 ml Axygen tube. Samples were incubated with 6 μl of a 6.25 mM TCEP‐HCl solution (Sigma) at 60°C for 30 min in a thermoblock with rotation at 400 rpm. Next, 6 μl of 40 mM MMTS was added and incubated for 30 min in the dark. Finally, 600 ng of trypsin gold (Promega) was added and digests were incubated at 37°C overnight. Digests were stopped with 10 μl of 10% TFA solution. 30% of the reaction volume was used for Nano LC–MS/MS analysis. Results from the promoter DNA affinity purification mass spectrometry are listed in Appendix Table S1.

#### Nano LC–MS/MS analysis for DNA affinity purification

An UltiMate 3000 RSLC nano HPLC system (Thermo Fisher Scientific) coupled to a Q Exactive HF‐X equipped with an Easy‐Spray ion source (Thermo Fisher Scientific) or an Exploris 480 mass spectrometer equipped with a Nanospray Flex ion source (Thermo Fisher Scientific) was used. Peptides were loaded onto a trap column (PepMap Acclaim C18, 5 mm × 300 μm ID, 5 μm particles, 100 Å pore size, Thermo Fisher Scientific) at a flow rate of 25 μl/min using 0.1% TFA as mobile phase. After 10 min, the trap column was switched in line with the analytical column (PepMap Acclaim C18, 500 mm × 75 μm ID, 2 μm, 100 Å, Thermo Fisher Scientific). Peptides were eluted using a flow rate of 230 nl/min, and a binary linear 3 h gradient, respectively, 225 min.

The gradient started with the mobile phases 98% A (0.1% formic acid in water) and 2% B (80% acetonitrile, 0.1% formic acid), increased to 35% B over the next 180 min, followed by a steep gradient to 90%B in 5 min, stayed there for 5 min, and ramped down in 2 min to the starting conditions of 98% A and 2% B for equilibration at 30°C (Köcher *et al*, 2012).

#### TFIIA immunoprecipitation

*Drosophila* S2 cells endogenously tagged with an AID‐3xFLAG were used for the bait, while the parental background cells only expression the osTir ligase were used as a control immunoprecipitation. Lysates were generated from 500 million cells. Cells were washed in PBS and pelleted by centrifugation. Cell pellet was resuspended in 10 ml of hypotonic swelling buffer (10 mM Tris pH7.5, 2 mM MgCl_2_, 3 mM CaCl_2_, protease inhibitors) and incubated for 15 min at 4°C. Cells were centrifuged for 10 min at 700 *g* and at 4°C. Cells were resuspended in 10 ml of GRO lysis buffer (10 mM Tris pH7.5, 2 mM MgCl_2_, 3 mM CaCl_2_, 0.5% NP‐40, 10% glycerol, 1 mM DTT, protease inhibitors) and rotated for 30 min at 4°C. Nuclei were centrifuged at 700 *g* and at 4°C. Supernatant was removed, and nuclei were resuspended in 1 ml of IP lysis buffer (100 mM NaCl, 20 mM HEPES pH7.6, 2 mM MgCl_2_, 0.25% NP‐40, 0.3% Tirton X‐100, 10% glycerol) and rotated for 30 min at 4°C. Lysed nuclei were centrifuged for 5 min at 20,000 *g* at 4°C. The supernatant containing the soluble nucleoplasm was kept. While the chromatin pellet was resuspended in a 300 mM NaCl IP lysis buffer (300 mM NaCl, 20 mM HEPES pH7.6, 2 mM MgCl_2_, 0.25% NP‐40, 0.3% Tirton X‐100, 10% glycerol) and sonicated Diagenode Bioruptor sonicator: 10 min (30 s on/30 s off) at low intensity. The sheared chromatin was centrifuged as before, and the soluble supernatant was removed and mixed with the soluble nucleoplasmic fraction. The resulting mixture was centrifuged again for 5 min at 20,000 *g* at 4°C to remove insoluble proteins. Anti‐FLAG M2 beads (Sigma‐Aldrich) were equilibrated by three 10 min washes with 150 mM NaCl IP lysis buffer and resuspended back in their original volume. Immunoprecipitation reactions were set up with 50 μl of Anti‐FLAG beads and 1 mg of the nuclear lysates overnight with rotation at 4°C. Immunoprecipitation reactions were magnetically pelleted and washed with 150 mM IP lysis buffer three times, 10 min each with rotation at 4°C. Next, to remove detergent, the reactions were washed four times, 10 min each at 4°C with a no‐detergent buffer (130 mM NaCl, 20 mM Tris pH7.5). Reactions were resuspended in 50 μl of 100 mM ammonium bicarbonate, and on‐bead tryptic digest was carried out as described in the DNA affinity purification and on‐bead digest section. Results of the TFIIA‐L immunoprecipitation are listed in Appendix Table S2.

#### Nano LC–MS/MS analysis for TFIIA‐L immunoprecipitation

A Q Exactive HF‐X mass spectrometer was operated in data‐dependent mode, using a full scan (*m/z* range 380–1,500, nominal resolution of 60,000, target value 1E6) followed by MS/MS scans of the 10 most abundant ions. MS/MS spectra were acquired using normalized collision energy of 27, isolation width of 1.4 *m/z*, resolution of 30,000, target value of 1E5, maximum fill time 105 ms. Precursor ions selected for fragmentation (include charge states 2–6) were put on a dynamic exclusion list for 60 s. Additionally, the minimum AGC target was set to 5E3 and intensity threshold was calculated to be 4.8E4. The peptide match feature was set to preferred, and the exclude isotopes feature was enabled.

#### LC–MS/MS analysis for TFIIA‐L immunoprecipitation

The Orbitrap Exploris 480 mass spectrometer (Thermo Fisher Scientific) was operated in data‐dependent mode, performing a full scan (*m/z* range 380–1,200, resolution 60,000, target value 3E6) at 2 different CVs (−50, −70), followed each by MS/MS scans of the 10 most abundant ions. MS/MS spectra were acquired using a collision energy of 30, isolation width of 1.0 *m/z*, resolution of 45,000, the target value of 1E5 and intensity threshold of 2E4 and fixed first mass of *m/z* = 120. Precursor ions selected for fragmentation (include charge state 2–5) were excluded for 30 s. The peptide match feature was set to preferred, and the exclude isotopes feature was enabled.

#### Mass spectrometry data processing

For peptide identification, the RAW files were loaded into Proteome Discoverer (version 2.5.0.400, Thermo Fisher Scientific). All hereby created MS/MS spectra were searched using MSAmanda v2.0.0.16129 (Dorfer V. *et al*, J. Proteome Res. 2014 August 1;13(8):3679–3684). RAW files were searched in two steps: First, against the *Drosophila* database called dmel‐all‐translation‐r6.34.fasta (Flybase.org, 22,226 sequences; 20,310,919 residues), or against an earlier version dmel‐all‐translation‐r6.17.fasta (21,994 sequences; 20,118,942 residues) / a small custom *Drosophila* database (107 sequences; 61,976 residues), each case supplemented with common contaminants, using the following search parameters: The peptide mass tolerance was set to ± 5 ppm and the fragment mass tolerance to ± 15 ppm (HF‐X) or to ± 6 ppm (Exploris). The maximal number of missed cleavages was set to 2, using tryptic specificity with no proline restriction. Beta‐methylthiolation on cysteine was set as a fixed modification, oxidation on methionine was set as a variable modification, and the minimum peptide length was set to seven amino acids. The result was filtered to 1% FDR on protein level and was used to generate a smaller subdatabase for further processing. As a second step, the RAW files were searched against the created subdatabase using the same settings as above plus the following search parameters: Deamidation on asparagine and glutamine were set as variable modifications. In some datasets acetylation on lysine, phosphorylation on serine, threonine and tyrosine, methylation on lysine and arginine, di‐methylation on lysine and arginine, tri‐methylation on lysine, ubiquitinylation residue on lysine, biotinylation on lysine, and formylation on lysine were set as additional variable modifications. The localization of the post‐translational modification sites within the peptides was performed with the tool ptmRS, based on the tool phosphoRS (Taus *et al*, 2011). Peptide areas were quantified using the in‐house‐developed tool apQuant (Doblmann *et al*, 2018). Proteins were quantified by summing unique and razor peptides. Protein‐abundances‐normalization was done using sum normalization. Statistical significance of differentially expressed proteins was determined using limma (Smyth, 2004).

#### PRO‐seq

PRO‐seq was performed according to (Mahat *et al*, 2016) with the following modifications. 10 million *Drosophila* Schneider S2 cells were used for each replicate, spiked in with 1% human HCT116 cells. Cells were harvested by centrifugation, and cells were permeabilized with cell permeabilization buffer (10 mM tris Ph 7.5, 300 mM sucrose, 10 mM CaCl_2,_ 5 mM MgCl_2_, 1 mM EGTA, 0.05% tween‐20, 0.1% NP‐40, 0.5 mM DTT, supplemented with protease inhibitors). Permeabilization was carried by resuspending the cells in 10 mM of permeabilization buffer and spinning down the cells for a total of three buffer exchanges. Nuclei were resuspended in 100 μl of storage buffer (10 mM tris pH 7.5, 25% glycerol, 5 mM MgCl_2_, 0.1 mM EDTA and 5 mM DTT) and snap frozen in liquid nitrogen for later use, or immediately proceeded to the run‐on reaction. Nuclear transcription run‐on was carried by adding 100 μl of a 2× run‐on buffer (10 mM tris pH8, 5 mM MgC_2_, 1 mM DTT, 300 mM KCl, 0.25 mM ATP, 0.25 mM GTP, 0.05 mM Biotin‐11‐CTP, 0.05 mM Biotin‐11‐UTP, 0.8 U/μl murine RNase inhibitor, 1% sarkosyl) and incubated at 30C for 3 min. Reaction was terminated by adding 500 μl Trizol‐LS. Extraction was performed by adding 130 μl of chloroform, after vortexing and centrifugation the aqueous fraction was kept and precipitated with 2.5 volumes of 100% ethanol and 1 μl of glycoblue. The pellet was washed with 80% ethanol, air‐dried, and resuspended in 50 μl of water. RNA was denatured at 65C for 40 s before base hydrolysis with 5 μl 1 N NaOH for 15 min. Hydrolysis was quenched with 25 μl of 1 M Tris–HCl pH6.8. Samples were purified on a Bio‐Rad P30 column. Biotinylated nascent RNA was recovered by incubating with 50 μl of M280 streptavidin beads for 30 min at room temperature with rotation. Beads were washed twice each with high salt buffer (2 M NaCl, 50 mM Tris pH 7.5, 0.5% Tirton X‐100) and binding buffer (300 mM NaCl, 10 mM Tris pH 7.5, 0.1% Tirton X‐100) and once with low‐salt buffer (5 mM Tris pH 7.5, 0.1% Tirton X‐100). RNA was extracted off the beads using Trizol and cleaned on a Direct‐zol column (Zymo). RNA was eluted from the column using 5 μl the 3′ RNA linker. Overnight ligation at 16°C was performed with T4 RNA ligase I. The following day biotinylated RNA was recovered with 50 μl of M280 streptavidin beads for 30 min at room temperature and washed as described previously. The RNA was treated with Cap‐CLIP Pyrophosphatase (Biozyme) on the beads for 1 h at 37°C, followed by T4 polynucleotide kinase (NEB) for 1 h at 37°C. Beads were washed as described and an on‐bead ligation was set up with T4 RNA ligase I and the 5′ RNA linker at room temperature with rotation at 4 h. Next, the beads were washed as described and the RNA was extracted off the beads with 300 μl Trizol and purified on a Direct‐zol column, eluted in water. Eluted RNA was used for reverse transcription with Superscript III Reverse Transcriptase (Thermo) according to the manufacturer's recommendations. Half of the reverse transcription reaction was used for amplification with a KAP real‐time PCR mixture (KAPA Biosystems) using the Illumina Truseq small RNA library amplification kit primers. Libraries were amplified in 8–12 cycles. Primer dimers were removed from the libraries with AMPURE beads and sent for next‐generation sequencing.

#### PRO‐seq data mapping

PRO‐seq libraries were sequenced to a depth of 3.8–38.9 million reads using single‐end sequencing and read length of 50 bp. We used unique molecular identifiers (UMIs) to distinguish between PCR‐duplicated identical reads and reads stemming from distinct RNA molecules with an identical sequence. The latter will have identical sequences but different UMIs and therefore allows more accurate quantification of transcripts. RNA oligos containing UMIs of 8–10 nt in length were ligated to the 3′ end of all reads before PCR amplification and then computationally removed to prevent interference during genome alignment. Cutadapt 1.18 (Martin, 2011) with default options was used to find and trim the sequencing adapter at the 3′ end and filtered for reads ≥10 nts long. Only after read alignment, we corrected for PCR duplicated transcripts and to more accurately quantified transcripts: Reads containing the same sequence and reads aligning to the same genomic position were collapsed to unique UMIs.

To align reads, we generated an artificial genome containing sequences for tRNAs and rRNAs only, which allows for noise reduction of short reads aligning to multiple positions. Next, all unmapped reads were captured using samtools version 1.9 (Li *et al*, 2009) with ‐f 4 option, which were then aligned to the *D. melanogaster* reference genome BDGP R5/dm3. Following this, reads not aligning to the dm3 genome were aligned to the *H. sapiens* reference genome GRCh37/hg19 (used as spike‐in). For genome alignment, we used bowtie version 1.2.2 (Langmead *et al*, 2009) allowing two mismatches (−v 2). For alignment to the artificial genome, we allowed reads having up to 1,000 reportable alignments, but reporting only the best alignment (−m 1,000 ‐‐best ‐‐strata) to meet the highly repetitive and conserved nature of tRNAs and rRNAs. Alignment to the reference genomes was run allowing only reads aligning uniquely (−m 1).

We generated an artificial genome containing the ribosomal RNA primary transcript CR45847 (http://flybase.org/reports/FBgn0267507), all annotated tRNA genes from Dmel 5.57 and tRNAs predicted from Genomic tRNA database, published 2009, http://lowelab.ucsc.edu/GtRNAdb/ (accessed August 17, 2020; http://lowelab.ucsc.edu/download/tRNAs/eukaryotic‐tRNAs.fa.gz). We used R packages GenomicRanges 1.34.0 (Lawrence *et al*, 2013), Biostrings 2.50.2 (https://bioconductor.org/packages/Biostrings) and BSgenome.Dmelanogaster.UCSC.dm3 1.4.0 (Team, 2017). *BSgenome.Hsapiens.UCSC.hg17: Full genome sequences for Homo sapiens (UCSC version hg17)*. R package version 1.3.1000.

Since application of the usual PRO‐seq protocol delivers reads corresponding to the reversed complement of the nascent RNA, the reads aligning to the minus strand originated from transcripts with the sequence on the plus strand and vice versa. Additionally, only the end of the transcript where RNA Pol II was actively transcribing was included for the downstream analysis. Reads were switched and shortened accordingly using the bigBedtoBed utility (Kent *et al*, 2010).

#### ChIP‐seq and ChIP‐exo data analysis

ChIP‐seq and ChIP‐exo datasets were taken from (Gurudatta *et al*, 2013; Baumann & Gilmour, 2017; Shao & Zeitlinger, 2017). Coverage was calculated over a 1‐kb window centered on the TSS of each promoter type. Data were normalized for the transcription level as measured by PRO‐seq, which was further normalized by gene length for each individual promoter.

#### Generation of browser tracks of PRO‐seq data

For visualization of PRO‐seq data, we converted bigBed files to bigWig files using kentUtils bigBedToBed utility (Kent *et al*, 2010), normalized by the number of reads aligned to dm3 (and considered number of reads aligned to hg19 for TFIIA samples), and calculated the coverage using genomeCoverageBed from bedtools 2.27.1 (Quinlan & Hall, 2010) before converting to a bigWig file using KentUtils wigToBigWig utility. BigWig files were visualized with the UCSC Genome Browser (Kent *et al*, 2010).

#### Differential expression

Differential expression was calculated using the DESeq function from the DESeq2 package v.1.30.1 (Love *et al*, 2014) providing the normalization factors as sizeFactors. Normalization factors were calculated based on quantified spike‐in reads. Specifically, for each sample, the ratio between reads mapping to the human genome and the *Drosophila* genome was used to determine the scaling factor representing the fold change of total transcriptional output between the samples. We used Benjamini–Hochberg‐adjusted *P*‐values to determine significantly deregulated transcripts.

#### STAP‐seq data analysis of initiation events

Cofactor recruitment STAP‐seq data from (Haberle *et al*, 2019) were analyzed at single‐nucleotide resolution counting unique transcripts initiated at each position in each tested oligo. The dominants TSS was determined as the position with the highest count, and the relative count was calculated by dividing the count at the dominant TSS with the total count for each oligo. To determine the number of activated TSSs in each oligo, the count at each position was divided by the count at the dominant TSS, and only the positions with a ratio of more than 20% were counted as activated TSSs.

#### Aligning CAGE data to promoter motif positions and +1 nucleosome centers

For the above‐defined promoter groups, the positions of the defining CP motifs were determined relative to the dominant CAGE TSS (if they occurred within ± 120 bp). Only promoters with a single occurrence of each motif were considered, and the position of the motif was used as a reference point to generate average plots of CAGE data. MNase‐seq data from Chereji *et al* (2016), CAGE data from mixed embryos (Hoskins *et al*, 2011).

MNase‐seq data were used to determine the position of the +1 nucleosome by taking the centers of MNase fragments between 100 and 200 bp long, calculating the coverage of such centers, and determining the position with the highest coverage in the region 150 bp downstream of the dominant CAGE TSS. These +1 nucleosome centers were used as a reference to generate average plots of CAGE data for each promoter group. Inversely, MNase‐seq data were plotted against the dominant CAGE TSS position to reveal the distribution of the +1 nucleosome positions in relation to the dominant TSSs (Appendix Fig S2).

Cross‐correlation analysis between CAGE and MNase‐seq reads (Appendix Fig S6I) was performed in relation to the dominant CAGE TSS in a flanking window of −50 to +200 base pairs. The cross‐correlation mean was plotted with the standard deviation for the three developmental promoter types (TATA‐box, DPE, and INR) and the three housekeeping promoter types (TCT, Ohler1, and DRE).

#### Luciferase assay

*Drosophila* Schneider S2 cells were plated in 96‐well plates, 1 × 10^5^ cells per well. Cells were transfected with 100 ng of luciferase plasmid containing a DRE promoter or negative control sequence upstream of the luciferase gene, and 100 ng of a plasmid containing Renilla luciferase as a transfection efficiency normalization control using Lipofectamine 2000. Cells were lysed 48 h after transfection with 50 μl passive lysis buffer for 30 min at room temperature with shaking. Lysates were further diluted 10‐fold in passive lysis buffer. 10 μl of the diluted lysate was placed in 96‐well plates compatible with luminescence read‐out and measured with the Promega dual‐luciferase assay kit according to the manufacturer's recommendation on a BioTek Synergy H1 plate reader.

For COF recruitment luciferase assay in AID cell lines, we have first transfected the luciferase reporter and Gal4‐COF expressing plasmids. After 24 h, we added 500 μM auxin and waited an additional 24 h prior to measurement of the luciferase signal.

#### Limitations of the study

In our study, we present evidence, indicating that functionally distinct promoter classes in *Drosophila* recruit the transcription machinery via different mechanisms. Part of the evidence is based on the binding of transcription‐related proteins to naked core promoter DNA *in vitro*, which differed substantially for different promoter types despite identical experimental conditions. While these findings indicate that the different promoter types differ in their DNA's intrinsic abilities to recruit transcription‐related proteins, the assays do not reflect the transcriptionally active situation of these promoters *in vivo*. The DNA fragments are not chromatinized and remodeling events that occur *in vivo* are not recapitulated (without these, housekeeping‐promoter‐bound BEAF‐32 and/or Ibf1/2 might for example inhibit PIC assembly). We therefore ask the readers to interpret each of these assays within their respective limits and in the context of the functional *in vivo* data provided elsewhere in the manuscript.

## Author contributions

**Leonid Serebreni:** Conceptualization; data curation; formal analysis; investigation; visualization; methodology; writing – original draft; writing – review and editing. **Lisa‐Marie Pleyer:** Data curation; formal analysis; investigation. **Vanja Haberle:** Data curation; formal analysis. **Oliver Hendy:** Investigation; methodology. **Anna Vlasova:** Data curation; formal analysis; methodology. **Vincent Loubiere:** Formal analysis; investigation; methodology. **Filip Nemčko:** Data curation; investigation; methodology. **Katharina Bergauer:** Investigation; methodology. **Elisabeth Roitinger:** Data curation; methodology. **Karl Mechtler:** Data curation; methodology. **Alexander Stark:** Conceptualization; resources; supervision; funding acquisition; investigation; writing – original draft; project administration; writing – review and editing.

## Disclosure and competing interests statement

The authors declare that they have no conflict of interest.

## Supporting information



Appendix

Expanded View Figures PDF

Dataset EV1

Dataset EV2

PDF+

## Data Availability

PRO‐seq data have been deposited to the Gene Expression Omnibus (GEO), accession GSE181257 (https://www.ncbi.nlm.nih.gov/geo/query/acc.cgi?acc=GSE181257). Raw mass spectrometry data of DNA affinity purification have been deposited to ProteomeXchange through the PRIDE server under identifier PXD028090 (http://proteomecentral.proteomexchange.org/cgi/GetDataset?ID=PXD028090) and mass spectrometry data of TFIIA‐L immunoprecipitation under identifier PXD028094 (http://proteomecentral.proteomexchange.org/cgi/GetDataset?ID=PXD028094).
