# Supplementary material for: Functionally distinct promoter classes initiate transcription via different mechanisms reflected in focused versus dispersed initiation patterns
Source: EMBO J. 2023 Apr 4;42(10):e113519. doi: 10.15252/embj.2023113519 (PMC10183819; doi:10.15252/embj.2023113519)
Supplement: Supplementary file 1 — Appendix [file EMBJ-42-e113519-s002.pdf]

Functionally distinct promoter classes initiate transcription via different mechanisms reflected in focused versus dispersed initiation patterns

Appendix

Table of Contents

1. Appendix Figure S1 page 2
2. Appendix Figure S2 page 3

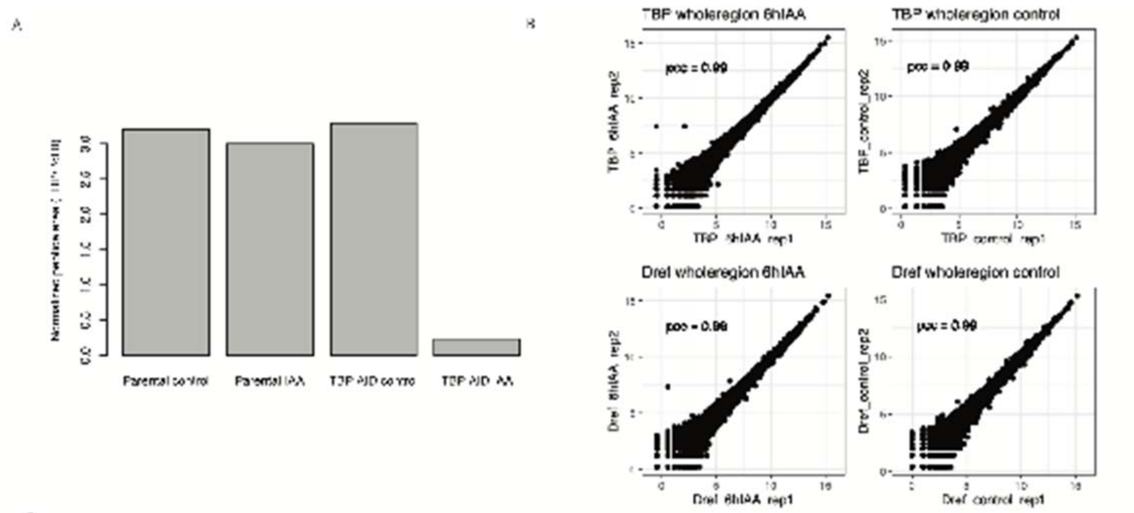

C

	motif	factor	N_expressed	pval_expressed	N_pulldown	pval_pulldown
1	DRE	DREF	1632	1.476829e-26	20	0.05412509
2	DRE	TBP	1640	2.386946e-14	20	0.20959174
3	TATAbox	DREF	741	1.476829e-26	24	0.05412509
4	TATAbox	TBP	747	2.386946e-14	24	0.20959174

Appendix Fig S1. TBP and DREF are required by distinct sets of promoters

A. Mass-spectrometric quantification of TBP protein abundance in the parental cell line expressing the Tir1 ligase and the TBP N-terminally tagged AID cell line after 6 hours of 500uM auxin treatment. Normalize peptide abundance from label free mass-spectrometric quantification indicates roughly 3% of TBP remains after auxin treatment as compared to the control.
B. Pearson correlation of PRO-seq signal along the promoter and gene body region of all protein-coding transcripts using library-normalized reads between biological replicates. Correlation coefficient displayed.
C. The number of DRE and TATA-Box expressed promoters in each of
the DREF and TBP AID tagged cell lines. P-value calculated with FDR indicate down-regulation of TATA-Box or DRE promoters compared with all expressed promoters.

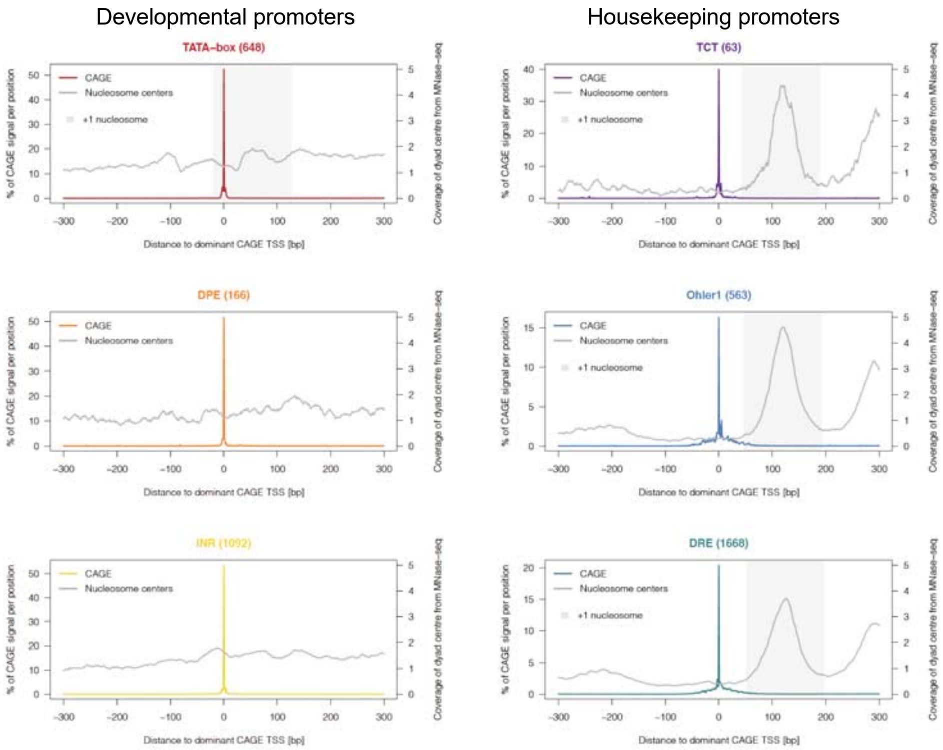

Appendix Fig S2. Housekeeping and developmental promoters differ in the +1 nucleosome positioning in relation to the TSS.

MNase-seq data from mix embryos (0-24 hours) obtained from Chereji et al., 2016 was plotted centered on the dominant CAGE annotated TSS for each motif-containing promoter type. Developmental promoters: TATA-box, DPE and INR; housekeeping promoters: TCT, Ohler1 and DRE. +1 nucleosome center is the point of highest coverage of MNase fragment centres in +1 to +200bp window relative to the TSS. Developmental promoters not showing a preferred nucleosomal position in relation to the TSS, while housekeeping promoters exhibit a peak downstream of the TSS.
