# Supplementary material for: Functionally distinct promoter classes initiate transcription via different mechanisms reflected in focused versus dispersed initiation patterns
Source: EMBO J. 2023 Apr 4;42(10):e113519. doi: 10.15252/embj.2023113519 (PMC10183819; doi:10.15252/embj.2023113519)
Supplement: Supplementary file 2 — Expanded View Figures PDF [file EMBJ-42-e113519-s004.pdf]

Expanded View Figures

Figure EV1. DNA affinity purifications uncover differentially bound proteins at functionally distinct promoters.

A Heat map of ~17,000 *Drosophila melanogaster* protein-coding gene promoters displaying % match to position weight matrix (PWM) score. Promoters were clustered with k-means clustering. Nine clusters emerged which display developmental (clusters 1–3) and housekeeping (clusters 5–7) motifs. Promoters in cluster 4 are enriched in Ohler8 and E-box motifs and can respond to both developmental and housekeeping coactivators as defined by Haberle, V. *et al*, 2019. Cluster 9 had no strong matches to any motif PWM.

B Pie chart of all expressed *Drosophila melanogaster* protein-coding gene promoters (~170,000) grouped based on motif content (left), and all expressed protein-coding genes from *Drosophila* S2 cells (~10,000). Only the main motif groups studied in this paper that are classified as housekeeping or developmental are shown. Group labeled as "other" contains promoters with motifs such as Ohler 8 and E-box or not motifs which could not be assigned as developmental or housekeeping.

C Luciferase activity assay measuring the basal or activated state of tested core promoter fragments. To measure basal activity, 121-bp-long promoter fragments cloned upstream of a luciferase gene (P). To measure the activated state of the core promoters we cloned the *Drosophila* Zdfh1 enhancer upstream of the promoter fragments (E + P). Plasmids were transfected into *Drosophila* S2 cells and activity was measured after 48 h. Firefly luciferase values were normalized to co-transfected Renilla luciferase values to control for transfection efficiency. Error bars represent standard deviation across four biological replicates.

D Rank plot of protein binding enrichment on TATA and DRE promoters over the control DNA pool from the DNA-purification mass spectrometry assay. Highlighted proteins are the Pol II PIC components and the DRE binding factor DREF.

E DNA-purification assay with a pool of 25 TATA-box promoters, and two individual TATA-box promoters in which the TATA-box was mutated (left panel). The assay was performed with a nuclear extract expressing TBP-FLAG that was tracked with a western blot. DNA-purification of a pool of 20 DRE promoters and three individual DRE promoters in which the DRE motif was mutated. The assay was performed with a nuclear extract expressing DREF-FLAG and followed with a western blot (right panel). Note that DREF binding is reduced to background levels while TBP is still slightly enriched compared with negative controls, consistent with TBP binding to non-TATA-box developmental core promoters (Fig 2B and E).

© 2023 The Authors

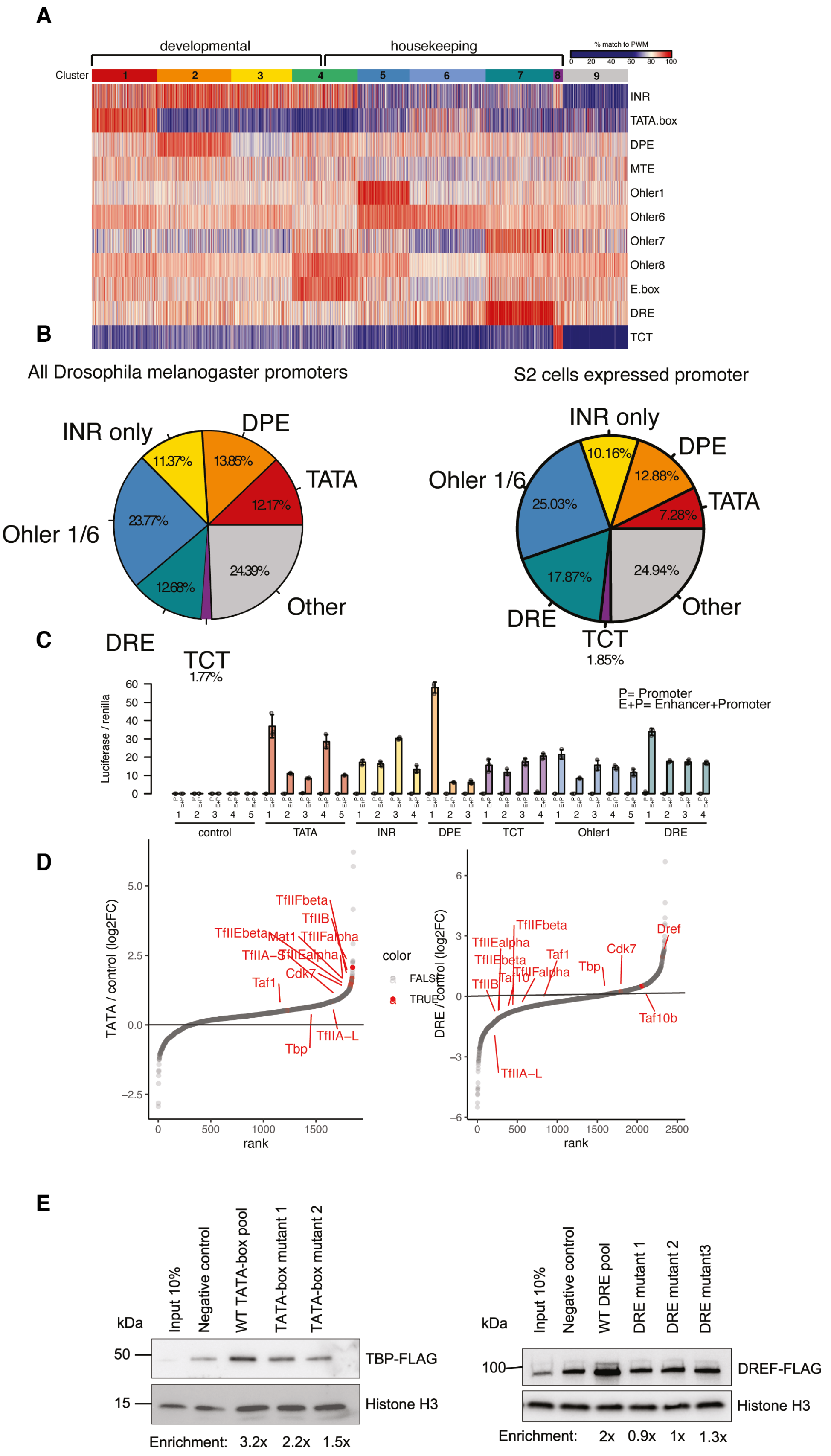

Figure EV1.

 © 2023 The Authors

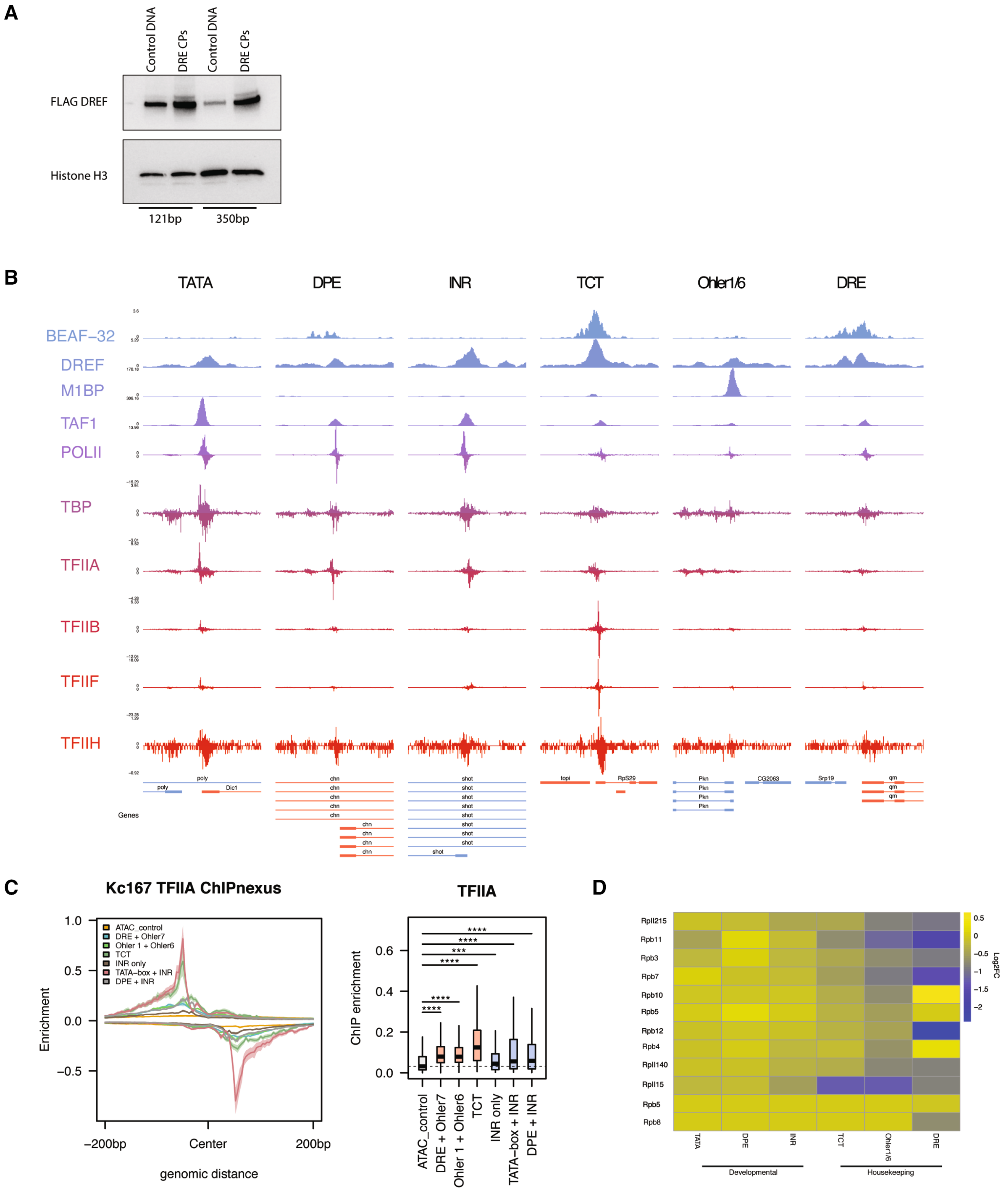
A
Control DNA
DRE CPs
Control DNA
DRE CPs
FLAG DREF
Histone H3
121bp
350bp
B
TATA
DPE
INR
TCT
Ohler1/6
DRE
BEAF−32
DREF
M1BP
TAF1
POLII
TBP
TFIIA
TFIIB
TFIIF
TFIIH
Genes
C
Kc167 TFIIA ChIPnexus
ATAC_control
DRE + Ohler7
Ohler 1 + Ohler6
TCT
INR only
TATA−box + INR
DPE + INR
Enrichment
genomic distance
−200bp
Center
200bp
TFIIA
ChIP enrichment
D
Log2FC
Developmental
Housekeeping

Figure EV2.

Figure EV2. Developmental and housekeeping promoters bind different sets of proteins and GTFs.

A Elution fractions from the DNA-purification assay with a pool of 20,121 bp or a pool of 10,350 bp DRE promoters and length-matched negative controls were performed with a nuclear extract expressing DREF-AID-3xFLAG tag and blotted for an anti-FLAG antibody. Both promoter lengths are able to enrich for DREF binding.

B Representative browser tracks of published ChIP-seq data of GTFs and promoter binding TFs (M1BP, DREF, BEAF-32) on the 6 different tested promoter types in this study.

C Meta-plot of TFIIA-L ChIP-seq data from panel E at the 6 different tested promoter types indicating TFIIA binds all active promoter types, although less strongly to housekeeping promoters and in a more dispersed fashion relative to the TSS (center). Box plot quantification of TFIIA ChIP-seq data at /+ 200 bp around the TSS. Boxes represent the upper and lower quartiles, with the middle band at the median. The whiskers represent 1.5 times the interquartile range across two biological replicates, outliers not shown. (****$P < 1e\text{-}5$, ***$P < 1e\text{-}3$, **$P < 1e\text{-}2$, *$P < 5e\text{-}2$, N.S = not significant).

D Heat map of log2FC values of DNA affinity purification values for RNA polymerase II subunits across the six different promoter types tested.

 © 2023 The Authors

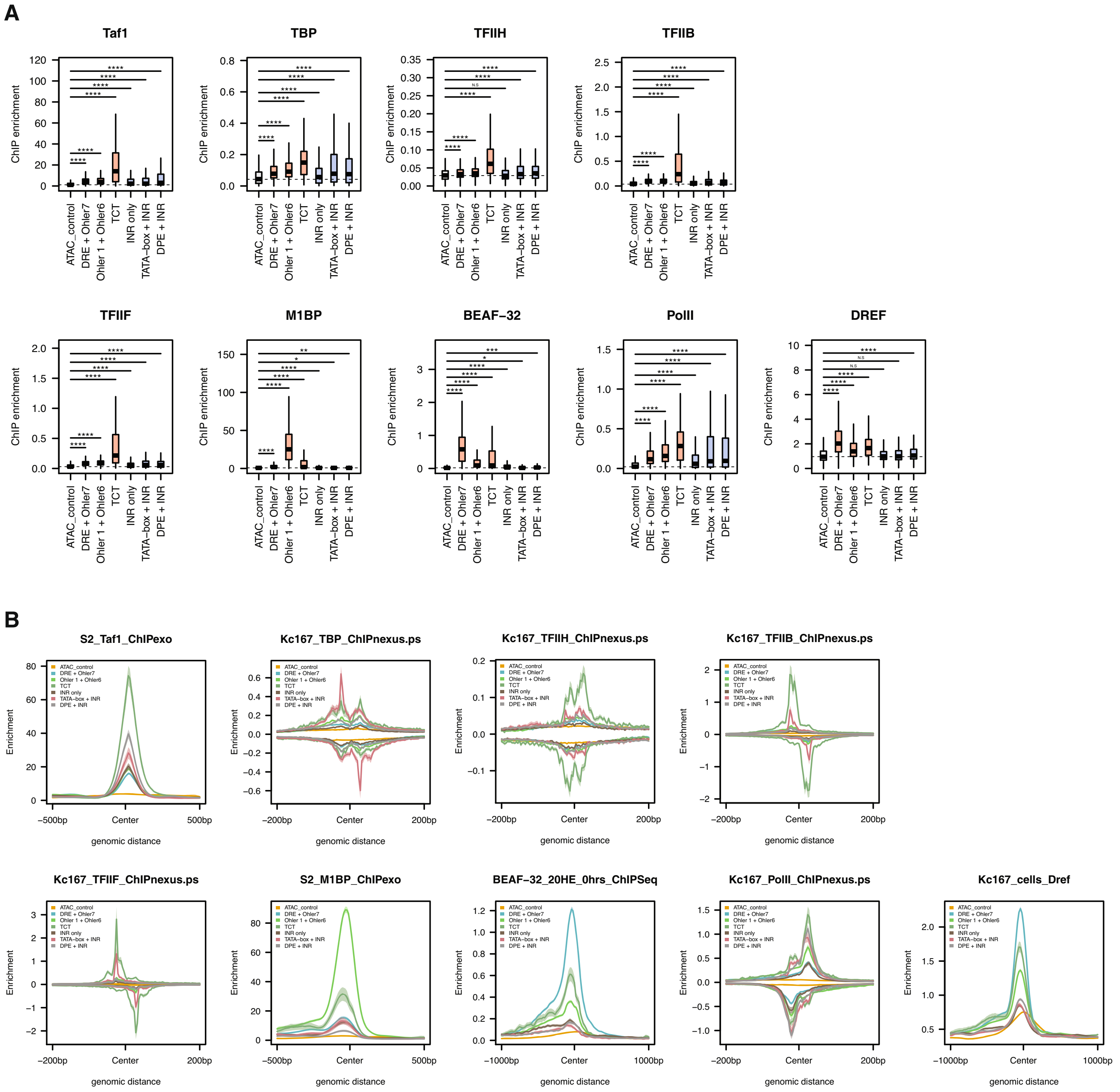

Figure EV3. Distribution of GTFs at developmental and housekeeping promoter.

A Box plots representing ChIP-seq signal of available GTFs and sequence-specific TFs on the six different promoter types tested from Drosophila melanogaster embryos centered on the TSS (200 bp to +200 bp). Wilcoxon test. Boxes represent the upper and lower quartiles, with the middle band at the median. The whiskers represent 1.5 times the interquartile range across two biological replicates, outliers not shown. (****$P < 1e\text{-}5$, ***$P < 1e\text{-}3$, **$P < 1e\text{-}2$, *$P < 5e\text{-}2$, N.S = not significant).

B Meta-plot of ChIP-seq signal of available GTFs and sequence-specific TFs (as in panel H) on the six different promoter types centered on the TSS.

© 2023 The Authors

Figure EV4. TBP and Trf2 regulate distinct subsets of developmental promoters.

A DNA affinity purification mass spectrometry enrichment values of TBP and Trf2 across the tested promoter types. Student's *t*-test ($P < 0.05$), three biological replicates per sample.

B ClustalW alignment of the short and long transcript isoforms of the *Drosophila melanogaster* TRF2 gene of the C-terminal region from 840 to 1,715 amino acids. Peptides detected from label-free mass spectrometric quantification of nuclear lysate from the TRF2-AID cell line are highlighted in yellow. All detected peptides are shared between the two isoforms.

C Normalized abundance of TRF2 peptides from lab label-free mass spectrometric quantification of nuclear lysates from the TRF2-AID cell line. Parental cell line is expressing the Tir1 ligase, while the TRF2-AID cell line is endogenously tagged with 3x-FLAG-AID. 500 μM Auxin treatment was performed for 6 h.

D Western blot of anti-FLAG antibody on the double-tagged TBP + Trf2 AID cell line visualizing TBP and Trf2 upon auxin addition, indicating a slower depletion kinetics of the TBP-AID protein.

E Normalized abundance of TBP in the TRF2-AID cell line and parental OsTir1 expressing cell line under control and 12 h 500 μM auxin treatment. Error bars represent the standard deviation across two biological replicates.

F Growth curve tracking the number of live cells for 4 days for individual TBP-AID, Trf2-AID, and double TBP + Trf2-AID cell lines. No growth differences are observed upon the individually tagged cell lines, but the double TBP + Trf2-AID cell line shows growth inhibition after addition of auxin. Error bars represent the standard deviation across three biological replicates.

G PRO-seq signal after TBP or Trf2 depletion (log2 fold change) is plotted for the TBP-dependent genes and Trf2-dependent genes. Boxes represent the upper and lower quartiles, with the middle band at the median. The whiskers represent the upper and lower 5th percentiles across two biological replicates.

H Auxin washout experiment in which TBP-AID or Trf2-AID cell lines were treated with auxin for 6 h and then washed twice and exchanged with fresh medium to remove auxin. qPCR performed on the tested time points on two tested genes indicate they can recover to their original level in the absence of auxin. Error bars represent standard deviation across three biological replicates.

I qPCR was performed on an auxin time-course treatment experiment. The tested genes were normalized to Actin5c levels. NLaz was identified from PRO-seq as dependent on TBP but not Trf2, and CG7408 was identified from PRO-seq to be dependent on Trf2 but not TBP. Three biological replicates were performed, mean fold change (log2) over a Gal4-DBD control of each sample is plotted with standard deviation with * for $P \leq 0.05$.

J *In silico* LyC and tryptic digestion of the Trf protein reveals predicted detectable peptides, which were not detected in mass spectrometry in our S2 cells, indicating a lack of Trf protein expression.

K ClustalW alignment of TBP and Trf. Peptides from TBP detected by mass spectrometry are highlighted in red. Peptides predicted from an *in silico* digest performed on Trf (from panel H) are highlighted in yellow.

L PRO-seq data of individual TBP, Trf2 and double-tagged TBP + Trf2 depletion at housekeeping promoters containing DRE, Ohler 1 and Ohler 6 motifs. These promoters are affected only upon depletion of Trf2 and to the same extent upon double depletion, demonstrating that TBP is dispensable for their expression and cannot substitute for Trf2 at these housekeeping promoters. Boxes represent the upper and lower quartiles, with the middle band at the median. The whiskers represent the upper and lower 5th percentiles across two biological replicates.

 © 2023 The Authors

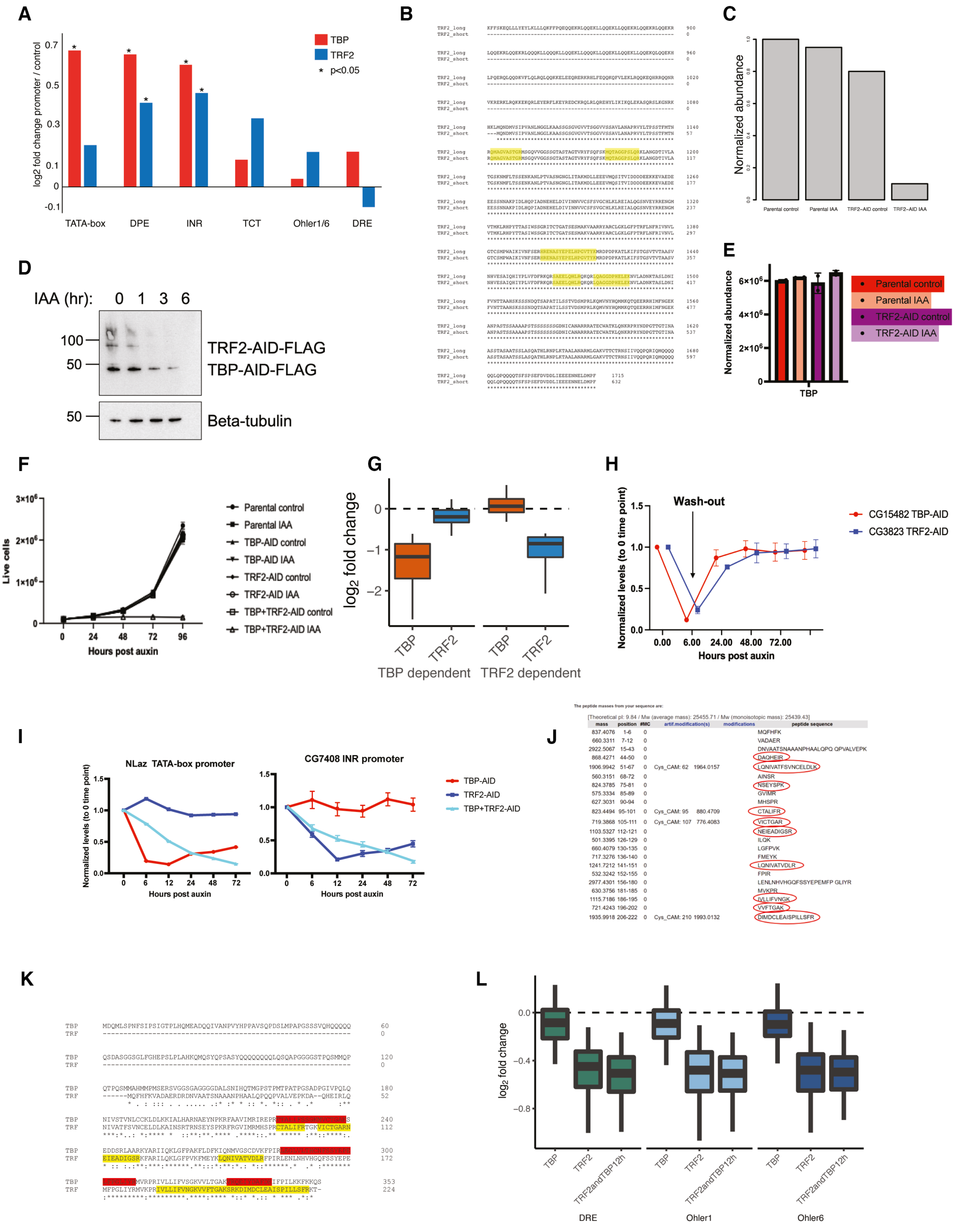

A
TBP
TRF2
p<0.05
log2 fold change promoter / control
TATA-box
DPE
INR
TCT
Ohler1/6
DRE
B
C
Normalized abundance
Parental control
Parental IAA
TRF2-AID control
TRF2-AID IAA
D
IAA (hr): 0 1 3 6
TRF2-AID-FLAG
TBP-AID-FLAG
Beta-tubulin
E
Parental control
Parental IAA
TRF2-AID control
TRF2-AID IAA
Normalized abundance
TBP
F
Live cells
Hours post auxin
Parental control
Parental IAA
TBP-AID control
TBP-AID IAA
TRF2-AID control
TRF2-AID IAA
TBP+TRF2-AID control
TBP+TRF2-AID IAA
G
log2 fold change
TBP
TRF2
TBP dependent
TRF2 dependent
H
Wash-out
Normalized levels (to 0 time point)
Hours post auxin
CG15482 TBP-AID
CG3823 TRF2-AID
I
NLaz TATA-box promoter
CG7408 INR promoter
Normalized levels (to 0 time point)
Hours post auxin
TBP-AID
TRF2-AID
TBP+TRF2-AID
J
The peptide masses from your sequence are:
K
L
log2 fold change
TBP
TRF2
TRF2andTBP12h
DRE
Ohler1
Ohler6

Figure EV4.

Figure EV5. TFIIA is required by all promoters and is recruited by housekeeping cofactors to housekeeping promoters.

A Growth curve of TFIIA-L-3xFLAG-AID cell line and parental Tir1 expressing control over 4 days upon the addition of 500 μM auxin. TFIIA-L-AID treated cells die after 24 h. Error bars represent standard deviation across three biological replicates.

B Fraction of reads mapping to the D. mel genome (reference species) and the human genome (spike-in) in PRO-seq experiments depleting TBP or TFIIA-L. A ~ 4-fold increase in proportion of reads mapping to the spike-in genome is observed only upon depletion of TFIIA-L due to global failure of Pol II transcription in the TFIIA-L-AID cell line.

C MA-plot of PRO-seq data in the TFIIA-L-AID cell line after 3 h of auxin treatment, showing a global failure of Pol II transcription.

D Caspase 3 and 7 activity was measured with the Promega Caspase 3/7 Glo kit of TFIIA-L-AID cells after addition of auxin at various time points. A positive cell death control was included as a 24 h treatment of 10 μg/ml puromycin.

E Volcano plot of TFIIA-L immunoprecipitation mass spectrometry. TFIIA-L was immunoprecipitated from the endogenously tagged TFIIA-L-3xFLAG-AID cell line using anti-FLAG beads. Enrichment was measured over control immunoprecipitation made from the Tir1 expressing parental cell line which does not contain any FLAG epitope. Three replicates were performed for each condition.

F Volcano plot of Chromator immunoprecipitation mass spectrometry. Chromator was immunoprecipitated from the Chromator-3xFLAG-AID cell line using an anti-FLAG antibody. Similar Tir1 expressing parental cell line control was used to measure enrichment. Putzig (Pzg) and GFZF are also highlighted.

G DNA affinity purification assay was performed with a 121-bp-long housekeeping DRE promoter with 4xUAS sites upstream. Initially, a nuclear extract containing a Gal4-DNA-binding domain fusion of GFP or GFZF was incubated with the bead-immobilized promoter DNA (left panel). After the incubation, the extract was removed, and the beads were used for a DNA affinity purification assay with a nuclear extract containing TFIIA-L-AID-3xFLAG as described in the materials and methods. Sheared salmon sperm DNA was used as competitor DNA at 600 ng to 1.6 μg per reaction. Elution fractions were run on an SDS–PAGE gel and blotted with a FLAG antibody (right panel).

H Western blot against FLAG antibody visualizing whole cell lysate from a TFIIA-L c-terminally tagged 3x-FLAG-AID line treated with auxin for 2 h. Full degradation of the TFIIA-L beta subunit is visible upon 2 h of auxin treatment.

 © 2023 The Authors

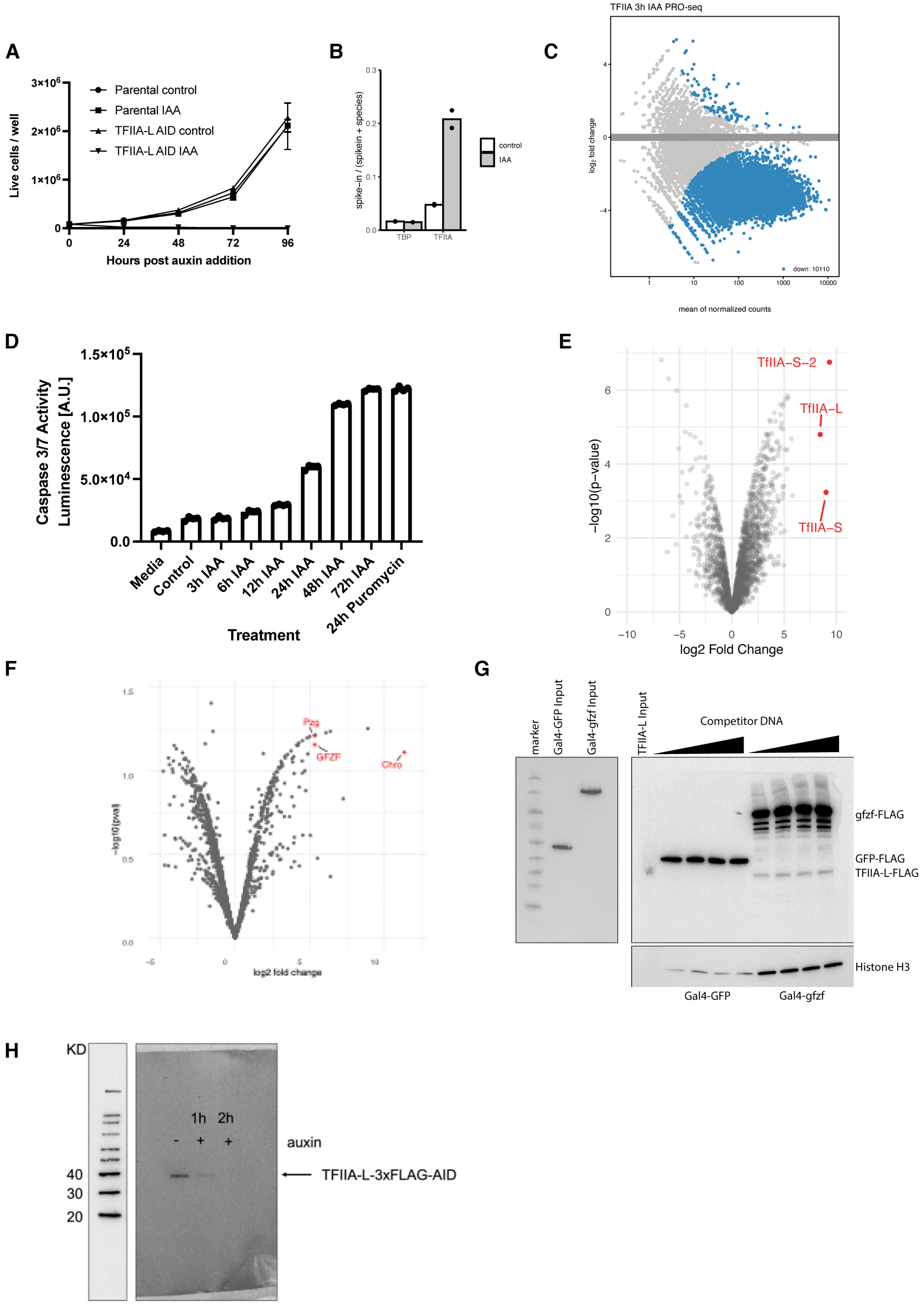
A
Live cells / well
Hours post auxin addition
Parental control
Parental IAA
TFIIA-L AID control
TFIIA-L AID IAA
B
spike−in / (spikein + species)
TBP
TFIIA
control
IAA
C
TFIIA 3h IAA PRO-seq
log2 fold change
mean of normalized counts
down: 10110
D
Caspase 3/7 Activity Luminescence [A.U.]
Media
Control
3h IAA
6h IAA
12h IAA
24h IAA
48h IAA
72h IAA
24h Puromycin
Treatment
E
TfIIA−S−2
TfIIA−L
TfIIA−S
−log10(p−value)
log2 Fold Change
F
Pzg
GFZF
Chro
log2 fold change
G
marker
Gal4-GFP Input
Gal4-gfzf Input
TFIIA-L Input
Competitor DNA
gfzf-FLAG
GFP-FLAG
TFIIA-L-FLAG
Histone H3
Gal4-GFP
Gal4-gfzf
H
KD
40
30
20
1h
2h
auxin
TFIIA-L-3xFLAG-AID

Figure EV5.

Figure EV6. Housekeeping cofactor recruitment is sufficient to recapitulate dispersed transcription initiation patterns.

A The number of CAGE-defined TSSs in each promoter type over a 120 ± bp region. TSS was defined as a position having at least 20% CAGE signal as the dominant TSS in the tested region.

B Frequency of dinucleotides at the −1/+1 position for the dominant and secondary TSSs in each promoter type in a 120 ± bp window.

C Fold change (log2) of STAP-seq signal upon GFZF or MED25 recruitment over GFP for oligos that are matched for their activation level by either one of both cofactors.

D Relative CAGE signal per position on all active promoters of the indicated type aligned to the +1 nucleosome center (point of highest coverage of MNase fragment centers in +1 to +200 bp window relative to TSS).

E Percent of STAP-seq signal at the dominant TSS for activation matched oligos (one activated oligo per gene TSS) for housekeeping and developmental promoters that can be activated by both MED25 and GFZF. Boxes represent the upper and lower quartiles, with the middle band at the median. The whiskers represent the upper and lower 5th percentiles across three biological replicates.

F Histogram showing the number of TSSs activated upon GFZF or MED25 recruitment on random regions that are responsive to both cofactors (left). Cumulative plot of the same data (right). *P*-values: Kolmogorov–Smirnov test.

G Scatter plot of the log2 fold change above GFP (i.e., activation) of promoters by GFZF or MED25 used in the analysis (i.e., matched to be activated to similar extent by both cofactors).

H Histogram representing distribution of the width of the initiation region (i.e., part of the oligo covered by STAP-seq signal) upon recruitment of MED25 or Putzig (Pzg), Med25 or Chro (Chromator), p300 or GFZF, Lpt or GFZF. For each comparison core promoters activated to similar extent by both analyzed cofactors were included. *P*-values: Wilcoxon rank-sum test.

I Cross-correlation analysis between CAGE and MNase-seq reads relative to the dominant CAGE TSS at developmental (TATA-box, DPE, INR) and housekeeping (TCT, Ohler1, DRE) promoters. The mean (line) and standard deviation (shaded area) for the cross-correlation are plotted at different offsets in a base-pair window of −50 to 200 in relation to the dominant TSS.

 © 2023 The Authors

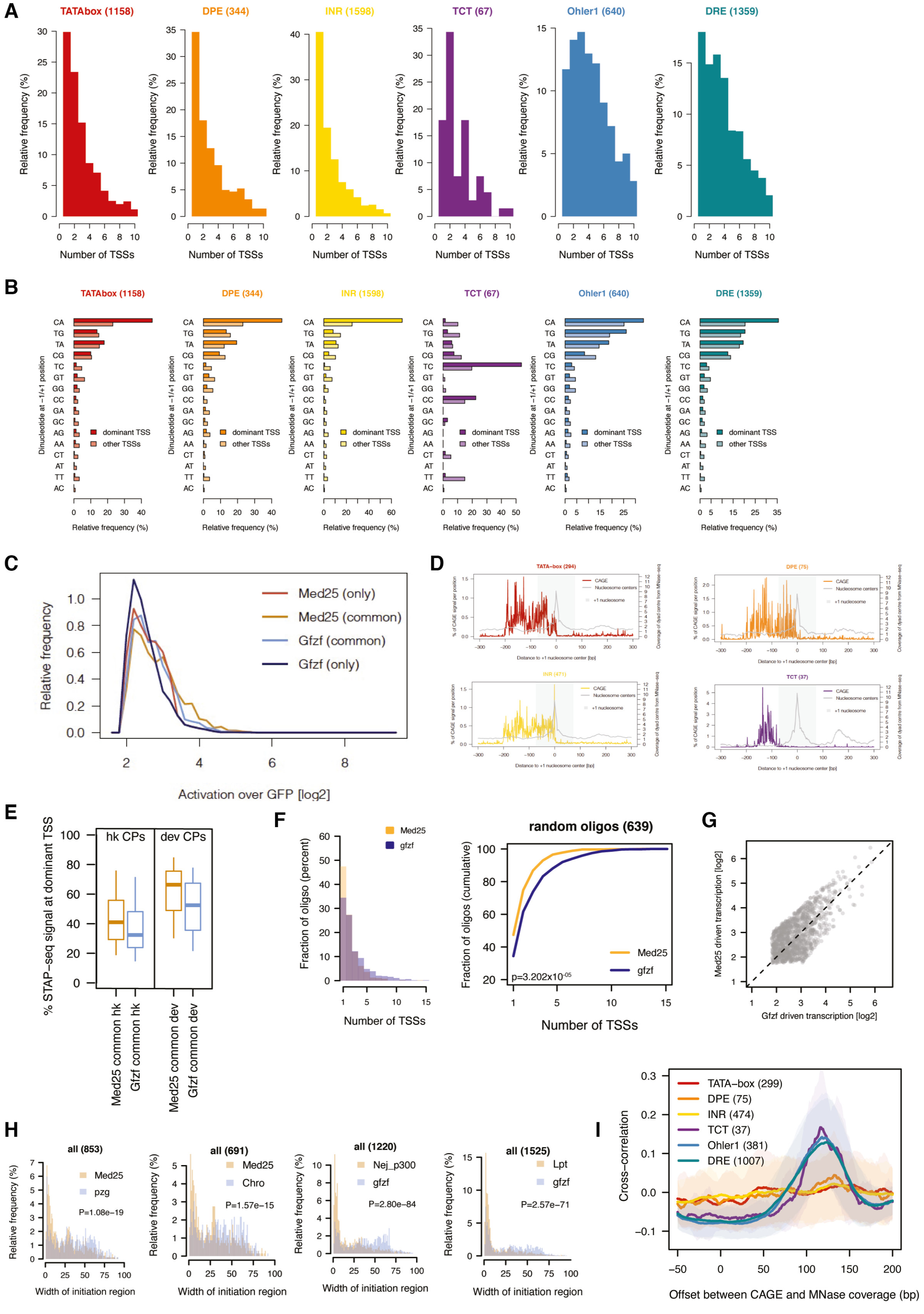
A
TATAbox (1158)
DPE (344)
INR (1598)
TCT (67)
Ohler1 (640)
DRE (1359)
Relative frequency (%)
Number of TSSs
B
Dinucleotide at −1/+1 position
dominant TSS
other TSSs
Relative frequency (%)
C
Med25 (only)
Med25 (common)
Gfzf (common)
Gfzf (only)
Relative frequency
Activation over GFP [log2]
D
E
hk CPs
dev CPs
% STAP−seq signal at dominant TSS
Med25 common hk
Gfzf common hk
Med25 common dev
Gfzf common dev
F
Med25
gfzf
Fraction of oligso (percent)
Number of TSSs
random oligos (639)
Fraction of oligos (cumulative)
p=3.202x10-05
G
Med25 driven transcription [log2]
Gfzf driven transcription [log2]
H
all (853)
Med25
pzg
P=1.08e−19
all (691)
Med25
Chro
P=1.57e−15
all (1220)
Nej_p300
gfzf
P=2.80e−84
all (1525)
Lpt
gfzf
P=2.57e−71
Relative frequency (%)
Width of initiation region
I
TATA−box (299)
DPE (75)
INR (474)
TCT (37)
Ohler1 (381)
DRE (1007)
Cross−correlation
Offset between CAGE and MNase coverage (bp)

Figure EV6.
