# Supplementary material for: Functionally distinct promoter classes initiate transcription via different mechanisms reflected in focused versus dispersed initiation patterns
Source: EMBO J. 2023 Apr 4;42(10):e113519. doi: 10.15252/embj.2023113519 (PMC10183819; doi:10.15252/embj.2023113519)
Supplement: Supplementary file 6 [file EMBJ-42-e113519-s001.pdf]

Functionally distinct promoter classes initiate transcription via different mechanisms reflected in focused versus dispersed initiation patterns

Leonid Serebreni, Lisa-Marie Pleyer, Vanja Haberle, Oliver Hendy, Anna Vlasova, Vincent Loubiere, Filip Nemčko, Katharina Bergauer, Elisabeth Roitinger, Karl Mechtler, and Alexander Stark
DOI: 10.15252/embj.2023113519

Corresponding author: Alexander Stark (stark@starklab.org)

Review Timeline:

Transfer from Review	15th Jan 23
Commons: Editorial	8th Feb 23
Decision: Revision	1st Mar 23
Received: Accepted:	9th Mar 23

Editor: Stefanie Boehm / Ieva Gailite

Transaction Report:

This manuscript was transferred to The EMBO Journal following peer review at Review Commons.
(Note: With the exception of the correction of typographical or spelling errors that could be a source of ambiguity, letters and reports are not edited. Depending on transfer agreements, referee reports obtained elsewhere may or may not be included in this compilation. Referee reports are anonymous unless the Referee chooses to sign their reports.)

Review #1

1. Evidence, reproducibility and clarity:

Evidence, reproducibility and clarity (Required)

Summary:

Serebreni et al. Dissect the mechanisms of distinct transcriptional regulation patterns for the housekeeping and developmental classes of genes in Drosophila S2 cells. The authors used two primary lines of experimentation to determine the factors involved in regulation at the core promoters of the different gene classes: in vitro DNA binding with mass spectrometry, and in vivo depletion of factors with transcriptomics. The authors find that general transcription factors bind more strongly to developmental (TATA-containing) promoters and speculate that GTFs interact more transiently with housekeeping promoters. In addition, the authors find distinct preferences for TBP/TRF2 at different types of core promoters and test the roles of cofactors and promoter architecture on differing patterns of transcriptional initiation.

Major comments:

The main conclusions of this work are that promoters of the different classes of genes display differing usage of GTFs and cofactors to promote transcription and likely recruit polymerase by different mechanisms. The in vivo experiments using factor depletion offer strong evidence that certain factors including TBP/TRF2 are differentially required for transcription at the housekeeping/developmental gene classes. The in-depth analysis of different promoter types combined with the genetic approaches outlined above provide compelling mechanistic insights into promoter-specific engagement of regulatory factors. In general, the data supports the authors' suggestions.
One important shortcoming of these experiments is in the in-vitro DNA binding analysis of GTFs at differing core promoter contexts. The lack of GTFs binding to the housekeeping promoters may be a reflection of low intrinsic transcription activity. If the housekeeping promoters don't assemble active transcription complexes in this in vitro system but the Developmentally-regulated promoters do, then a simple comparison of proteins bound to each promoter type is potentially misleading as to the factors required for transcription. For example, results of the in-vivo analysis suggest that the +1 nucleosome is an important factor in the positioning of the transcription start site at housekeeping promoters, therefore the use of chromatinized templates rather than naked DNA would likely better reflect the intrinsic binding properties of factors at promoters. One way to address this issue is to test transcription activity of the promoters used in the mass spec analysis. After incubation of promoters with extract, add NTPs and quantitate the basal transcription activity of each type of promoter. If they are the ~same - great. If not, at a minimum, the authors need to acknowledge this as a limitation of the study. The suggested transcription experiment is a simple extension of the work already completed.
The authors suggest from the depletion experiments of TBP/TRF2 that the factors are functionally redundant since the level of transcription for target genes recovers after prolonged

depletion, though there is not specific functional evidence to support this claim. A suggested experiment to test the functional redundancy of TBP/TRF2 at subsets of genes is to assess the levels of proteins and/or protein binding to promoters after factor depletion. For instance, is there a global upregulation/stabilization of TBP after TRF2 depletion? Or is there an increase in TBP binding at promoters? These can be addressed by western blot for overall protein levels and ChIP-seq or similar method to assess binding to promoters, which are fairly straightforward experiments given that the cells lines have already been produced.
A discussion would be appreciated on the generality of the suggested mechanism in metazoans. For example, is DREF conserved only in insects but could other eukaryotes use a similar mechanism at housekeeping genes?

Minor comments:

The manuscript is very difficult to read. One major problem is the large number of figures - many of which are not essential for understanding the results. I strongly suggest that the authors think carefully about which figures to include in the manuscript and keep only the most important. Second, the legends on many of the graphs are very tiny and difficult to read. Third, it would greatly help readability if the main figures and legends were imbedded in the manuscript and if the supplemental figures + legends were in a separate document.

Fig 4E: very difficult to understand what was done.

Fig 4A vs G: why are ~ the same number of genes affected by TRF2 vs TBP + TRF2 depletion? I got the impression from the text that there should be a large difference in the number of affected genes.

Fig 5A and similar figures: include the number of affected genes in the figure.

Fig S2C: hard to understand what was done from the legend.

Fig S2F and similar figures: hard to distinguish the legend and the green colors used.
Proofreading: Add citation for Cut&run in the methods. In supplemental Fig1a, the percentage of "INR only" is greater than 100%. Supplemental Fig 1a legend-should 170,000 protein coding genes read "17,000"? Santana et al. reference on pg 8 should read 2022.

Readability: The categorizations of genes classes based on core promoter elements is somewhat unclear-from 1a, is it the case that all TATA contain INRs? A different way of representing the data to capture overlaps in motifs other than a pie chart may better convey these motif relationships. Work could be done to increase clarity in general on the promoter motif subtypes used and how mutually exclusive these elements are in the tested subsets.

Figure 5: authors state "all protein coding genes" are downregulated with TFIIA depletion, though it appears some transcripts are unchanged or upregulated in 5B/C. Suggest change in language.

A discussion on the developmental context of the S2 cell line seems appropriate. If S2 cells

represent a late stage developmental cell line, would the authors expect the relative utilization of cofactors to be the same/different in other cellular contexts?

2. Significance:

Significance (Required)

This work is conceptually significant due to the large in gene-specific regulatory mechanisms in the field of molecular biology. In addition, the authors propose a new mechanism whereby PIC formation is substantially different at different gene classes. Much of our mechanistic understanding of the role of general transcription factors is limited to highly expressed, typically TATA-containing genes, though several lines of research have shown that not all genes are dependent on the same subsets of factors. Notably, TBP has been shown to be required for the transcription of only small subsets of genes in specific cell types, therefore investigations into the roles of general factors at diverse genes is an important step in the field. This work is also technologically significant due to its use of the auxin-inducible degron system to assess the immediate transcriptional effects of factor depletion. Prior work demonstrated that long-term loss of factors through genetic deletions can often lead to compensatory mechanisms including utilization of alternative regulatory pathways and stabilization of cellular RNAs, therefore assessment of the immediate effects of rapid factor depletion is a powerful approach to determine regulatory mechanisms. This research will be of broad interest to molecular biologists studying the basic mechanisms of transcription as well as gene-specific regulation.

Reviewer expertise:

Transcriptional regulation, biochemistry, genomics, molecular biology

3. How much time do you estimate the authors will need to complete the suggested revisions:

Estimated time to Complete Revisions (Required)

(Decision Recommendation)

Between 1 and 3 months

4. *Review Commons* values the work of reviewers and encourages them to get credit for their work. Select 'Yes' below to register your reviewing activity at Web of Science Reviewer Recognition Service (formerly Publons); note that

the content of your review will not be visible on Web of Science.

Web of Science Reviewer Recognition

Yes

Review #2

1. Evidence, reproducibility and clarity:

Evidence, reproducibility and clarity (Required)

The article from Serebreni, Stark and co-workers combines biochemical, analytical, computational and cellular methods to uncover different factor dependencies for different classes of promoters in Drosophila. The results are compelling and the data support the conclusions. Important new insights are that housekeeping and developmental promoters have different requirements for initiation factors and that TFIIA is generally required across the different promoter types. Also, the article provides evidence of potential new mechanisms that control focused vs. dispersed initiation. These are groundbreaking results and I have only a few minor comments on the article.

1. The DNA affinity purification method is excellent as a discovery method, but it has some potential caveats. One is that it cannot capture remodeling events that could potentially remove otherwise stably bound factors to allow for transient PIC assembly and gene activation. It is possible that some of the insulator factors such as BEAF-32 and Ibf1/2, which selectively bind housekeeping sequences, could prevent or reduce binding by PIC factors. This could occur if BEAF-32 and/or Ibf1/2 inhibit PIC assembly if bound to DNA and if these factors bind housekeeping promoters with high affinity and slow off-rates. That is, in live cells, a competition could exist between binding of these enriched housekeeping factors and PIC assembly. By contrast, this caveat is not relevant at developmental promoters due at least in part to low/sub-nM TBP binding affinity. Ultimately, this is a minor concern but the authors should address in the article to inform readers about potential limitations of the experiments.
2. More information about how the PRO-seq spike-ins were implemented is recommended. For example, were they fit to a linear regression of read counts/chromosome between all samples, or did they take all hg19 reads as raw fold-change of all samples compared to a control replicate?
3. Figure S1C should be cited (not S1B) to support the statement "Mutating either the TATA box or DRE motifs reduced TBP or DREF binding to control levels..."
4. The authors could note that TATA box mutants still show slight enrichment for TBP compared to negative controls.
5. In Figure 2A, it would help to remind readers here that TATA, DPE, INR = developmental and TCT, Ohler1/6, DRE = housekeeping.
6. Figure S2A shows only 121bp and 350bp DRE core promoters but the text refers to 450bp and

1000bp sequences as well. Can the authors show representative results from these longer sequences?
7. In comparing data in Fig 2B and 2E, it seems the statement "the ChIP signals reflected the differential binding preferences observed in vitro for the respective promoter subtypes" should be modified. It is true to an extent but it is more nuanced than indicated by the text.
8. In Fig S2I, Ohler1 + Ohler6 and TCT are difficult to distinguish because of color scheme choice.
9. In Fig 3F, perhaps add that Gld has TATA and Fit2 has DRE?
10. Fig 5D: legend is cut off in the Figure.
11. Fig S2B needs more description and clarification in the main text and the legend.
12. Page 8, 2nd paragraph "avoiding potential" should be replaced with "minimizing" or similar.
13. Page 16, penultimate paragraph: "Essentially" should be replaced with "Essentiality"

2. Significance:

Significance (Required)

As noted in the prior section, the results break new ground and will be of interest to many in the field of gene regulation, broadly defined.

3. How much time do you estimate the authors will need to complete the suggested revisions:

Estimated time to Complete Revisions (Required)

(Decision Recommendation)

Less than 1 month

4. *Review Commons* values the work of reviewers and encourages them to get credit for their work. Select 'Yes' below to register your reviewing activity at [Web of Science Reviewer Recognition Service]() (formerly Publons); note that the content of your review will not be visible on Web of Science.

Web of Science Reviewer Recognition

No

Review #3

1. Evidence, reproducibility and clarity:

Evidence, reproducibility and clarity (Required)

Summary

The manuscript by Serebreni and colleagues examines how core promoter elements influence the binding of general transcription factors and co-activators and the establishment of pre initiation complexes, and how the recruitment of these factors relate to the transcription initiation patterns (focused versus dispersed) within Drosophila promoters. While there is extensive literature on core promoter elements and their association with general transcription factors and promoter classes, a mechanistic link between promoter sequence and dispersed initiation patterns has been lacking. Therefore, the present study is important. Using an impressive range of well planned experiments, combining in vitro (DNA-affinity purification, STAP-seq) and in vivo (CAGE, PRO-seq, ChIP-seq) data, the authors conclude that developmental promoters directly recruit the PIC via positional core promoter elements leading to a focused transcription initiation pattern while housekeeping promoters facilitate PIC recruitment through intermediate binding of additional cofactors leading to a more dispersed promoter initiation pattern. This conclusion is strengthened by experimental data demonstrating increased TSS dispersion upon forced recruitment of cofactors naturally associated with promoters exhibiting dispersed initiation.

Major comments

1. The authors perform a k-means clustering of PWM match scores within 17,000 promoter sequences. They describe in the Methods section that this data revealed 9 groups of promoters. However, although it is likely that several of these promoters contain matches for multiple core promoter motifs, the promoter classes are simply named DRE-promoters, TATA-promoters, TCT-promoters, etc., disregarding any combinatorial association. Furthermore, the clustering data is not visualized to support this naming. The authors should at least provide a heatmap showing the PWM match scores for these clusters and indicate which promoters were used. This is crucial for interpretation of results.
2. Relatedly, this paper uses a seemingly over-simplified terminology to describe promoters as housekeeping or developmental. While this terminology has been used in several studies from the Stark lab, this is not well supported by data and the usage of this terminology will likely lead to confusion among readers. Here, housekeeping seems to refer solely to the presence of a motif match in the promoter sequence rather than to ubiquitous expression across cell types. Similarly, developmental promoters seem to refer to anything that is not housekeeping. Are S2 cells best reflecting the activity of developmental genes? What about genes that are not expressed as part of a specific developmental trajectory, but still cell-type restricted? Since focus here is on the behavior of promoters with respect to their core promoter elements, why not just refer to them according to their promoter elements? A good example where the developmental versus housekeeping distinction is not useful is the authors' desire to generalize differences observed in Figure 2B, in which it is quite obvious that there is no clear developmental versus housekeeping

split. Rather the data demonstrate that TATA-containing and DRE-containing promoters behave differently.
3. The authors state that the "prevalent model" in the community is that PIC assembly is the same at all promoters. This is not true. For instance, it is well established that certain core promoter elements have a strong positional effect on TSS selection, while dispersed promoters lack strong positional features. What is less known is how the dispersed pattern, e.g. of non-TATA promoters, arises. The authors should more clearly specify the unknowns and the novel findings of their paper.
4. One of the major claims made by the authors in the paper is that PIC is recruited directly or indirectly depending on the presence of TATA or DRE. However, their approach seems to pick up a lot of indirect bindings, especially for TATA. This raises concerns of potential biases, which if addressed would strengthen the author's claims. The results do not exclude that TFIIA is directly recruited to TATA but might simply reflect stronger binding to other factors compared to DRE. It is also puzzling that DRE is the only one selected for further validation as it appears to have the lowest affinity for PIC binding and the focus on Ohler1/6 motifs in the final model. Disclaimer, this reviewer is not an expert on DNA-affinity purification assays.
5. Their final model is supported by results by Baumann et al (2018), which directly shows binding and interactions between M1BP, putzig, gfzf and TRF2. However, these factors bind to Ohler1, while most of the work within this study (Figures 1, 3) focused on DRE. How do DRE-containing promoters fit with the final model? Currently, these promoters are not even represented in the model figure.

Minor comments

1. The TSS patterns of promoters were evaluated using STAP-seq (in vitro data) and developmental CAGE data. For the purpose of the paper and to match the in DNA-affinity purification data better, it would be more reasonable to make use of S2 cell CAGE data (e.g. Rennie et al, 2018 PMID: 29659982).
2. Previous models on TSS selection within non-TATA promoters have highlighted the dinucleotide frequency of +1 nucleosomal DNA as a strong positional feature. Here, the authors investigate this model using a rather weak analytical approach. We know that nucleosomes can vary between cells (fuzzy positioning). Variability across promoters may cause larger variability in relative TSS positioning. Hence, what is observed here as a TSS spread relative to the +1 nucleosome positioning might in fact be caused by averaging. A more suitable approach would be to analyze the positional cross-correlation between TSS locations (e.g. revealed by CAGE reads) and nucleosomal positions (e.g. revealed by MNase-seq reads). This would better support claims regarding specific TSS positioning with respect to nucleosome positioning.
3. It is interesting that tethering of housekeeping-associated coactivators leads to a higher positional dispersion compared to the result of developmental-associated coactivators. However, the positional TSS dispersion of housekeeping promoters seems to always be larger than that of developmental promoters regardless of coactivator recruitment. Can the authors explain these results?
4. The authors seem to suggest that positional dispersion of TSSs within housekeeping promoters is due to stochastic initiation after non-positional specific PIC recruitment mediated via certain co-activators. If TSS selection is truly stochastic, why do these promoters then have dominant TSSs?

5. The authors find Chromator as a likely cofactor for indirect recruitment of TFIIA to housekeeping promoters. BEAF-32 is another factor the authors highlight as being enriched at housekeeping promoters (DRE promoters). Both of these factors have previously been considered insulator proteins or architectural proteins involved in the formation of chromatin folding (Ramirez et al, 2018, PMID: 29335486; Wang et al, 2018. PMID: 29335463). Could the authors comment on this link with their own findings?
6. Caan the authors justify PWM match thresholds used and why these were changed from Haberle et al 2019?
7. Figure related comments/concerns:
- General: Sometimes wrong ordering of figure panels with regards to their first mention in the main text, varying font sizes, and minimal figure legends that are often inconsistent (e.g. PRO-seq is sometimes specified when used, but not always)
- Typo: Supp Fig 1: INR only 121.37%
- Fig 1E not explained, what does x axis describe and how is it calculated?
- Figure 2C-D: The CAGE signal is poorly visualized in panel C, it also poorly describes that this is supposedly done using a pool of promoters. Where is the 450bp blot (it seems plausible that the 450bp fragment could actually facilitate a luciferase signal in Fig S2-B)? How was this pool selected, is it exclusively based on DRE-containing promoters?
- Fig 2D: apparent gel leakage and loading on the second panel is low. Preferably, provide positive control on the same gel.
- Figure 4C: all classes are negatively affected by TRF2 depletion, thus enrichment (4B) makes little sense here
- Figure 5C: Missing axis labels
- Figure 6F: A y scale would help here

2. Significance:

Significance (Required)

The manuscript by Serebreni and colleagues examines how core promoter elements influence the binding of general transcription factors and co-activators and the establishment of pre initiation complexes, and how these factors relate to the transcription initiation patterns (focused versus dispersed) of promoters in Drosophila. While there is extensive knowledge on core promoter elements and how these relate to TSS positional dispersion within promoters, little is known about the mechanism of PIC assembly at non-TATA promoters and how this influences TSS selection. The findings will therefore be interesting for a general audience, although it is unclear how transferable results are to other organisms.

The authors use an impressive range of well planned experiments, combining in vitro (DNA-affinity purification, STAP-seq) and in vivo (CAGE, PRO-seq, ChIP-seq) data. Their main conclusion is that developmental promoters directly recruit the PIC via positional core promoter elements leading to a focused transcription initiation pattern while housekeeping promoters facilitate PIC recruitment through intermediate binding of additional cofactors leading to a more dispersed promoter initiation pattern.

While this major conclusion is of interest to the community, the manuscript unfortunately falls

short in some regards, in particular in its over-generalizations and simplifications. Throughout the manuscript, the analysis is focused around specific core promoter motifs while ignoring the fact that many of these tend to co-occur within a promoter. In addition, the authors make general statements about housekeeping versus developmental promoters - a terminology based solely on the presence of core promoter elements - rather than attributing their findings to the core-promoter elements themselves. Lastly, the main figures are unpolished with minimal information provided in figure legends, making it sometimes difficult to follow the author's reasoning and raising concerns about the strength of their findings.

Fields of expertise: mammalian regulatory elements, transcription initiation, genomics

3. How much time do you estimate the authors will need to complete the suggested revisions:

Estimated time to Complete Revisions (Required)

(Decision Recommendation)

Between 1 and 3 months

4. *Review Commons* values the work of reviewers and encourages them to get credit for their work. Select 'Yes' below to register your reviewing activity at Web of Science Reviewer Recognition Service (formerly Publons); note that the content of your review will not be visible on Web of Science.

Web of Science Reviewer Recognition

No

Full Revision

Manuscript number: RC-2022-01719
Corresponding author(s): Alexander, Stark

1. General Statements [optional]

This section is optional. Insert here any general statements you wish to make about the goal of the study or about the reviews.

In our manuscript titled "Functionally distinct promoter classes initiate transcription via different mechanisms reflected in focused versus dispersed initiation patterns" we present evidence indicating that functionally distinct promoter classes in Drosophila recruit the transcription machinery via different mechanisms. In this rebuttal, we addressed the reviewers' comments by revising figures and text according to their recommendations and by discussing the limitations of the study in the "limitations of the study paragraph".

2. Point-by-point description of the revisions

Reviewer #1

Major comments:
The main conclusions of this work are that promoters of the different classes of genes display differing usage of GTFs and cofactors to promote transcription and likely recruit polymerase by different mechanisms. The in vivo experiments using factor depletion offer strong evidence that certain factors including TBP/TRF2 are differentially required for transcription at the housekeeping/developmental gene classes. The in-depth analysis of different promoter types combined with the genetic approaches outlined above provide compelling mechanistic insights into promoter-specific engagement of regulatory factors. In general, the data supports the authors' suggestions.

One important shortcoming of these experiments is in the in-vitro DNA binding analysis of GTFs at differing core promoter contexts. The lack of GTFs binding to the housekeeping promoters may be a reflection of low intrinsic transcription activity. If the housekeeping promoters don't assemble active transcription complexes in this in vitro system but the developmentally-regulated promoters do, then a simple comparison of proteins bound to each promoter type is potentially misleading as to the factors required for transcription. For example, results of the in-vivo analysis suggest that the +1 nucleosome is an important factor in the positioning of the transcription start site at housekeeping promoters, therefore the use of chromatinized templates rather than naked DNA would likely better reflect the intrinsic binding properties of factors at promoters.

We thank the reviewer for highlighting that the in vivo experiments constitute strong evidence for the differential requirements of certain factors at different promoter types and that our work provides compelling mechanistic insights into promoter-specific engagement of regulatory factors. We are also grateful to the reviewer for pointing out that we had not sufficiently clearly explained the aim and rationale of the initial in vitro DNA binding analyses (Figures 1 & 2). These which were not meant to assess different factor requirements but to assess if short core-promoter DNA is sufficient recruit transcription-related proteins, as had been reported for TATA promoters, and whether different core-promoter types differ in this ability. We therefore based the in vitro DNA binding assays on the fact that 121bp-short TATA core-promoter DNA is able to recruit and assemble the PIC even in the absence of activators, i.e. when the core promoters are transcriptionally inactive, and assayed all other core-promoter types under identical conditions. Interestingly, while the TATA core promoters enrich for canonical PIC components as expected, housekeeping promoter DNA does not, suggesting that the core-promoter DNA fragments' abilities to recruit and assemble the PIC differs.

We agree with the reviewer that one could possibly find conditions in which the different promoter types are active *in vitro*, e.g. by providing activators or chromatinized templates, and we hope that our explanations above clarify why this has not been the goal of these analyses. As the reviewer pointed out, we assay functional requirements of various TFs and GTFs *in vivo* in the remainder of the manuscript. We revised the manuscript to improve clarify the aim and scope of these sections (pages 4-9) and are grateful to the author for allowing a discussion of this topic as alternative (see below), many thanks

One way to address this issue is to test transcription activity of the promoters used in the mass spec analysis. After incubation of promoters with extract, add NTPs and quantitate the basal transcription activity of each type of promoter. If they are the ~same - great. If not, at a minimum, the authors need to acknowledge this as a limitation of the study. The suggested transcription experiment is a simple extension of the work already completed.

As outlined above, we deliberately assay all core promoter types under identical conditions, such that differences in protein binding reflect the different DNA fragments distinct functional properties. Please also note that while all core-promoter fragments are transcriptionally inactive, they can be activated by input from a strong enhancer (please see Supplementary Figure 2C; housekeeping and developmental core promoters can be induced to comparable levels, and thus weaker binding of GTFs to housekeeping promoters is not a reflection of weaker inducibility or activity).

We note that all statements and claims are strictly in line of what we tested, namely the core promoter DNA's ability to recruit transcription-related proteins *in vitro*. However, we agree with the reviewer that the notion that the core promoters are assayed under identical conditions but are not active is important and discuss it in the main text (pages 8 – 9) and the 'limitations of this study' section.

The authors suggest from the depletion experiments of TBP/TRF2 that the factors are functionally

redundant since the level of transcription for target genes recovers after prolonged depletion, though there is not specific functional evidence to support this claim. A suggested experiment to test the functional redundancy of TBP/TRF2 at subsets of genes is to assess the levels of proteins and/or protein binding to promoters after factor depletion. For instance, is there a global upregulation/stabilization of TBP after TRF2 depletion? Or is there an increase in TBP binding at promoters? These can be addressed by western blot for overall protein levels and ChIP-seq or similar method to assess binding to promoters, which are fairly straightforward experiments given that the cells lines have already been produced.

We thank the reviewer for suggesting potential compensatory mechanism regarding the redundancy of TBP and TRF2 at a subset of tested promoters. To address the question regarding the stability of TBP or TRF2 in the absence of one or the other, we have performed label-free quantitative mass spectrometry on the TRF2-AID cell line and examined TBP levels (Supplementary Figure 4E). We do not see a stabilization of TBP upon the depletion of TRF2 with auxin. The apparent functional redundancy (e.g. Fig. 4J) thus indeed suggests that there might be increased TBP binding. Unfortunately, we are not able to directly test this experimentally due to a lack of resources. We now add a discussion of the potential compensatory mechanisms to the main text (page 14), many thanks.

A discussion would be appreciated on the generality of the suggested mechanism in metazoans. For example, is DREF conserved only in insects but could other eukaryotes use a similar mechanism at housekeeping genes?

We agree that some of the specific TFs don't have one-to-one orthologs outside insects, yet that other prominent features of Drosophila housekeeping promoters are shared more widely. We now discuss the parallel between dispersed patterns of initiation at different promoter types across species, including Drosophila housekeeping and vertebrate CpG island promoters. We also provide an outlook towards future functional, biochemical and structural studies that might reveal more diverse transcription initiation mechanisms at the different promoter types in our genomes (pages 23-24).

Minor comments:
The manuscript is very difficult to read. One major problem is the large number of figures - many of which are not essential for understanding the results. I strongly suggest that the authors think carefully about which figures to include in the manuscript and keep only the most important.
We agree that the manuscript is complex with six main figures and several different approaches, including biochemistry and mass spectrometry but also genomics and bioinformatics. In addition, the manuscript includes *in vitro* tests of DNA-protein binding and *in vivo* assays to probe functional requirement (by depletion) and sufficiency (by recruitment). These different assays assess different properties and complement and validate each other, which is why we feel they are required. We hope that the clarification of the different aspects and their purpose makes the manuscript more easily accessible, many thanks.
Second, the legends on many of the graphs are very tiny and difficult to read.

Full Revision

We have revised the figures to improve font size and readability of the figures, many thanks.
Third, it would greatly help readability if the main figures and legends were imbedded in the manuscript and if the supplemental figures + legends were in a separate document.
We have now included the main figures and legends into the manuscript, thanks.
Fig 4E: very difficult to understand what was done.
We now add further explanations to the figure legend to describe the different promoter groups compared in the analysis of ChIP-seq coverage of TBP and TRF2.
Fig 4A vs G: why are ~ the same number of genes affected by TRF2 vs TBP + TRF2 depletion? I got the impression from the text that there should be a large difference in the number of affected genes.
We had the same prior expectation, but indeed observed a similar number of downregulated genes upon TRF2 depletion versus TBP and TRF2 double depletion. This may partly be technical, e.g. relating to clonal selection of the different AID-cell lines or thresholding effects, but is likely explained by the relatively few TBP dependent genes (200) that don't contribute substantially to the larger group of TRF2 dependent genes (3826). The observed number 3935 is 98% of the sum, even ignoring potential overlap. We now clarified this in the text.
Fig 5A and similar figures: include the number of affected genes in the figure.
We added the number to the figure, thanks.
Fig S2C: hard to understand what was done from the legend.
We have added additional explanations to the figure legend, thanks.
Fig S2F and similar figures: hard to distinguish the legend and the green colors used.
Proofreading: Add citation for Cut&run in the methods.
We did not analyze CUT&RUN data, however ATAC-seq and ChIP-seq data sets are cited.
In supplemental Fig1a, the percentage of "INR only" is greater than 100%.
We thank the reviewer and fixed the typo.
Supplemental Fig 1a legend-should 170,000 protein coding genes read "17,000"? Santana et al. reference on pg 8 should read 2022.
We thank the reviewer and fixed the typos
Readability: The categorizations of genes classes based on core promoter elements is somewhat unclear-from 1a, is it the case that all TATA contain INRs? A different way of representing the data to capture overlaps in motifs other than a pie chart may better convey these motif relationships. Work could be done to increase clarity in general on the promoter motif subtypes used and how mutually exclusive these elements are in the tested subsets.
We thank the reviewer for the suggestion. We have added a heatmap in Supplementary Figure 1A showing the percent match score to motif PWMs across Drosophila promoters. As the reviewer suspects, most developmental core promoters have a high-scoring INR motif and some have an additional TATA box or DPE motif. We have also revised the remainder of the text and rewritten the methods section regarding the motif analysis (pages 36 to 38) to improve clarity. Many thanks.
Figure 5: authors state "all protein coding genes" are downregulated with TFIIA depletion, though it appears some transcripts are unchanged or upregulated in 5B/C. Suggest change in language.

Review COMMONS

We thank the reviewer for this comment. Less than 70 genes are not downregulated upon TFIIA depletion, and manual inspection shows that these genes include intronic non-coding RNAs such as tRNAs that hinder accurate PRO-seq quantification. However, we agree with the reviewer and revised the text to reflect that essentially all promoters are downregulated, affecting all promoter types.
A discussion on the developmental context of the S2 cell line seems appropriate. If S2 cells represent a late stage developmental cell line, would the authors expect the relative utilization of cofactors to be the same/different in other cellular contexts?
We thank the reviewer for this comment. We indeed expect the relative utilization of cofactors to be the same I most cellular contexts and now added a discussion with relevant references (page 23), many thanks.

Reviewer #2

1. The DNA affinity purification method is excellent as a discovery method, but it has some potential caveats. One is that it cannot capture remodeling events that could potentially remove otherwise stably bound factors to allow for transient PIC assembly and gene activation. It is possible that some of the insulator factors such as BEAF-32 and Ibf1/2, which selectively bind housekeeping sequences, could prevent or reduce binding by PIC factors. This could occur if BEAF-32 and/or Ibf1/2 inhibit PIC assembly if bound to DNA and if these factors bind housekeeping promoters with high affinity and slow off-rates. That is, in live cells, a competition could exist between binding of these enriched housekeeping factors and PIC assembly. By contrast, this caveat is not relevant at developmental promoters due at least in part to low/sub-nM TBP binding affinity. Ultimately, this is a minor concern but the authors should address in the article to inform readers about potential limitations of the experiments.

We thank the reviewer for highlighting that DNA affinity purification is an excellent discovery method and for pointing out important differences between such *in vitro* assays and the *in vivo* situation. We agree and interpret our results from the DNA affinity purification carefully and specifically regarding differences observed for different types of core promoters under identical experimental conditions. We now highlight these differences more clearly throughout the relevant sections on pages 4-8 and expand the discussion of this issue in the 'limitations of the study' section. Many thanks.

2. More information about how the PRO-seq spike-ins were implemented is recommended. For example, were they fit to a linear regression of read counts/chromosome between all samples, or did they take all hg19 reads as raw fold-change of all samples compared to a control replicate?

We thank the reviewer for addressing the insufficient information provided about the spike-ins used for PRO-seq. We have added this information to the materials and methods section: We calculated the ratio of spiked-in reads representing the percentage of reads mapping to the human genome over all reads. This ratio was used to determine a scaling factor representing the fold-change of total transcriptional

output between the auxin-treated sample and the control samples.

3. Figure S1C should be cited (not S1B) to support the statement "Mutating either the TATA box or DRE motifs reduced TBP or DREF binding to control levels..."

We thank the reviewer for this correction and implemented the correct panel citation.

4. The authors could note that TATA box mutants still show slight enrichment for TBP compared to negative controls.

We now note this in the figure legend and explain that it is consistent with TBP binding to non-TATA-box developmental core promoters (Figure 2 B & E).

5. In Figure 2A, it would help to remind readers here that TATA, DPE, INR = developmental and TCT, Ohler1/6, DRE = housekeeping.

We thank the reviewer for this suggestion and implement it

6. Figure S2A shows only 121bp and 350bp DRE core promoters but the text refers to 450bp and 1000bp sequences as well. Can the authors show representative results from these longer sequences?

We thank the reviewer for pointing out these inconsistencies, which we now fixed by revisions to the text and supplementary figures.

7. In comparing data in Fig 2B and 2E, it seems the statement "the ChIP signals reflected the differential binding preferences observed in vitro for the respective promoter subtypes" should be modified. It is true to an extent but it is more nuanced than indicated by the text.

We have reworded the section and now discuss the observed trends for GTFs and TFs.

8. In Fig S2I, Ohler1 + Ohler6 and TCT are difficult to distinguish because of color scheme choice.

We agree and now explain in the figure legend that the brighter green corresponds to the Ohler1/6 promoters and the darker green to the TCT promoters, we have additionally edited the legend for better color visibility, many thanks.

9. In Fig 3F, perhaps add that Gld has TATA and Fit2 has DRE?

We now indicate the presence of TATA-box and DRE motifs in the figure, thanks.

10. Fig 5D: legend is cut off in the Figure.
We thank the reviewer for this comment and now fixed the cropped legend.
11. Fig S2B needs more description and clarification in the main text and the legend.
We now deleted Fig.S2B.
12. Page 8, 2nd paragraph "avoiding potential" should be replaced with "minimizing" or similar.
We thank the reviewer for this comment and have changed the word choice.
13. Page 16, penultimate paragraph: "Essentially" should be replaced with "Essentiality"
We thank the reviewer for this comment and correct the wording.

Reviewer #3

1. The authors perform a k-means clustering of PWM match scores within 17,000 promoter sequences. They describe in the Methods section that this data revealed 9 groups of promoters. However, although it is likely that several of these promoters contain matches for multiple core promoter motifs, the promoter classes are simply named DRE-promoters, TATA-promoters, TCT-promoters, etc., disregarding any combinatorial association. Furthermore, the clustering data is not visualized to support this naming. The authors should at least provide a heatmap showing the PWM match scores for these clusters and indicate which promoters were used. This is crucial for interpretation of results.

We thank the reviewer for pointing out the description of the motif analysis lacked clarity and that the clustering of Drosophila promoters should be visualized. We agree and now provide the k-means clustering heatmap of all 17118 protein coding gene promoters, visualizing the position-weight-matrix (PWM) scores matches for the different promoter motifs in Supplementary Figure 1A. This visualization confirms the reviewer's suspicion that core-promoter motifs often co-occur in the same core-promoter. For example, TATA promoters typically contain TATA-boxes and INR motifs, etc, which is now clearly seen in the newly provided heatmap. We have also revised the main text, figure legends and have rewritten the method section (pages 36-38) to clarify the analysis of motifs throughout the manuscript. Many thanks.

2. Relatedly, this paper uses a seemingly over-simplified terminology to describe promoters as housekeeping or developmental. While this terminology has been used in several studies from the Stark lab, this is not well supported by data and the usage of this terminology will likely lead to confusion among readers. Here, housekeeping seems to refer solely to the presence of a motif match in the promoter sequence rather than to ubiquitous expression across cell types. Similarly, developmental promoters seem to refer to anything that is not housekeeping. Are S2 cells best reflecting the activity of developmental genes? What about genes that are not expressed as part of a specific developmental trajectory, but still cell-type restricted? Since focus here is on the behavior of promoters with respect to their core promoter elements, why not just refer to them according to their promoter elements? A good

example where the developmental versus housekeeping distinction is not useful is the authors' desire to generalize differences observed in Figure 2B, in which it is quite obvious that there is no clear developmental versus housekeeping split. Rather the data demonstrate that TATA-containing and DRE-containing promoters behave differently.

We thank the reviewer for raising a concern about the terminology of functionally distinct promoter types in Drosophila. The use of functionally distinct promoter types enriched in different motifs is built on extensive evidence by our lab and others (e.g. the Ohler or Kadonaga groups) that found extensive agreement between promoter sequence, promoter function, initiation pattern, gene annotation, and ubiquitous vs. cell-type-restricted activities. Ubiquitously active housekeeping promoters tend to contain the TCT, DRE and Ohler 1/6 motifs, while cell-type-restricted developmental promoters tend to contain TATA-box, DPE and INR motifs (Arnold & Zabidi, Nat Biotech 2017, Haberle et al. Nature 2019, Ngoc et al. Genetics 2019, Ohler et al. Genome Biol 2002, Ohtsuki et al. Genes & Dev 1998, Rach et al. Plos Genetics 2011).

We find that the terminology is simple and thus accessible for the non-specialist reader. We agree with the reviewer that clarity is key and revise the introduction of the terminology to clarify that it is based on multiple lines of evidence. We also clarify that Figure 2B – in contrast to the reviewer's claim – does support a clear developmental versus housekeeping split (please see the dendrogram on top of the heatmap). We now clarified this in the main text and legend to Figure 2B, many thanks.

3. The authors state that the "prevalent model" in the community is that PIC assembly is the same at all promoters. This is not true. For instance, it is well established that certain core promoter elements have a strong positional effect on TSS selection, while dispersed promoters lack strong positional features. What is less known is how the dispersed pattern, e.g. of non-TATA promoters, arises. The authors should more clearly specify the unknowns and the novel findings of their paper.

We agree with the reviewer that certain core promoter elements have strong positioning effects on TSS selection and that these occur in promoters with focused initiation patterns such as TATA promoters and developmental non-TATA promoters (e.g. promoters with INR and/or DPE motifs). We also agree that it is unclear how dispersed patterns at housekeeping promoters arise, especially because the initiation sites don't co-occur with the TF motifs present in these promoters (e.g. DRE or M1BP motifs; see Figure 6A).

However, the question we address goes beyond TSS selection: we have not seen any study of PIC recruitment and assembly at any promoter with dispersed initiation pattern and the idea of a single uniform Pol II PIC assembly has been the predominant view of transcription initiation during the past two decades (Schier & Taatjes, Genes & Dev 2020). Here, we provide evidence that protein recruitment and GTF usage differs between promoter types, which has mechanistic implications beyond TSS choice alone. In particular, we show that at least two modes of transcription initiation exist that differ between

focused developmental and dispersed housekeeping promoters, whereby the developmental promoter DNA directly engages the Pol II PIC via TBP and TFIID, while the housekeeping promoter DNA does not and instead, housekeeping promoters recruit TFs, which recruit COFs and TFIIA. This is exciting and inconsistent with uniform GTF recruitment and assembly, and we hope that this work motivates the study of these different PIC assembly mechanisms at different promoter types.

4. One of the major claims made by the authors in the paper is that PIC is recruited directly or indirectly depending on the presence of TATA or DRE. However, their approach seems to pick up a lot of indirect bindings, especially for TATA. This raises concerns of potential biases, which if addressed would strengthen the author's claims. The results do not exclude that TFIIA is directly recruited to TATA but might simply reflect stronger binding to other factors compared to DRE. It is also puzzling that DRE is the only one selected for further validation as it appears to have the lowest affinity for PIC binding and the focus on Ohler1/6 motifs in the final model. Disclaimer, this reviewer is not an expert on DNA-affinity purification assays.

We thank the reviewer for pointing out that we had not sufficiently clearly explained the DNA affinity purifications. They were performed under identical conditions for all promoter types, such that the differential binding to TATA vs DRE promoters reflects the respective promoter DNA's affinity to various transcription-related proteins – they are key results of our work. Please note that, despite the high number of TATA interactions, many of these interactors are expected and reflect the binding of multi-subunit protein complexes such as the Mediator and TFIID (please see Figure 2B) and reflect the fact that we did not purify the PIC nor reconstitute it from purified components but determine nuclear proteins that bind to TATA-box promoter DNA. We now introduce and discuss these aspects more clearly.

It is possible that the fewer interactors found for housekeeping promoters stem from lower affinity of the PIC, the lack of chromatin, or the stable binding of sequence-specific binders such as DREF, BEAF-32 and M1BP in our assay (please see our response to reviewer 2 above). As these result from identical experiments under identical conditions, the fewer interactors for housekeeping promoters are also an important result that likely reflects lower affinity or more transient binding. We now clarify these results and their interpretation in the main text and discuss differences between this assay and transcription *in* vivo in the "limitations of the study" paragraph.

As the reviewer might appreciate, the follow up experiments, including the creation of AID cell lines, PRO-seq, etc., are a lot of work such that we did them for promoters at the two extreme ends of the spectrum and their respective DNA-binding factors TBP and DREF identified in Figure 1. We think that these representatives sufficiently strongly demonstrate that PIC assembly and factor requirement is distinct for different promoter types, many thanks.

5. Their final model is supported by results by Baumann et al (2018), which directly shows binding and

interactions between M1BP, putzig, gfzf and TRF2. However, these factors bind to Ohler1, while most of the work within this study (Figures 1, 3) focused on DRE. How do DRE-containing promoters fit with the final model? Currently, these promoters are not even represented in the model figure.

We thank the reviewer for pointing out that the final model highlights the Ohler 1 motif but omits the DRE motif. Based on the functional analyses shown in Figure 6 (pages 19-21), we think that the different motifs function equivalently in recruiting housekeeping cofactors and activating housekeeping transcription and have now included DRE motifs in the final cartoon. Our original choice was indeed based on the fact that previous reports from Baumann et al 2018 corroborate our findings for M1BP. As DRE promoters also recruit and depend on TRF2 (Hochheimer et al. Nature 2002), we now show a model by which housekeeping DRE promoters recruit a TRF2 containing PIC through TFIIA, but would like to stress that both likely function equivalently, leading to dispersed initiation. We also revised the data presentation and the final discussion regarding these promoters, many thanks.

Minor comments

1. The TSS patterns of promoters were evaluated using STAP-seq (in vitro data) and developmental CAGE data. For the purpose of the paper and to match the in DNA-affinity purification data better, it would be more reasonable to make use of S2 cell CAGE data (e.g. Rennie et al, 2018 PMID: 29659982).

We thank the reviewer for bringing up this point. For figure 6 we have used CAGE data from Drosophila embryos instead of S2 cells in order to capture a larger proportion of expressed developmental genes and their promoters, rather than just the ones that are expressed in S2 cells. As promoter motifs are found in stereotypical positions in relation to the TSS (Ohler et al. Genome Biol 2002) and because non-S2-cell core promoters can be activated in STAP-seq (Arnold 2017; Haberle 2019), our use of CAGE data from Drosophila embryos allows us to base all subsequent analyses on many more core promoters and also exclude any cell-type specific effects that may arise in TSS selection.

2. Previous models on TSS selection within non-TATA promoters have highlighted the dinucleotide frequency of +1 nucleosomal DNA as a strong positional feature. Here, the authors investigate this model using a rather weak analytical approach. We know that nucleosomes can vary between cells (fuzzy positioning). Variability across promoters may cause larger variability in relative TSS positioning. Hence, what is observed here as a TSS spread relative to the +1 nucleosome positioning might in fact be caused by averaging. A more suitable approach would be to analyze the positional cross-correlation between TSS locations (e.g. revealed by CAGE reads) and nucleosomal positions (e.g. revealed by MNase-seq reads). This would better support claims regarding specific TSS positioning with respect to nucleosome positioning.

We agree that the analysis of cross correlation between TSS locations and nucleosomal positions at individual promoters would provide a more precise measure of TSS positioning relative to the

nucleosome. We had originally chosen a visualization that more directly assesses whether the +1 nucleosome determines the TSSs by centering on the predicted +1 positions. In response to this comment, we have performed two additional analyses: a cross-correlation analysis on CAGE and Mnase-seq read coverage in relation to the dominant CAGE TSS (new Supplementary Figure 6I) and a TSS-centric analysis of Mnase-seq coverage (new Supplementary Figure 7. Both analyses agree with the original analysis and we thank the reviewer for pointing out how to strengthen this analysis.

The cross-correlation analysis reveals a peak in the mean correlation score 125 base pairs downstream of housekeeping TSS (at TCT, Ohler1 and DRE) promoters but not downstream of developmental promoters (TATA-box, DPE and INR), in line with housekeeping TSS being positioned upstream of the +1 nucleosome.

The analysis assessing +1 nucleosome positions as derived from MNase-seq coverage relative to the position of the dominant TSS reveals the expected phasing of downstream nucleosomes in housekeeping promoters but not at developmental promoters. Many thanks.

3. It is interesting that tethering of housekeeping-associated coactivators leads to a higher positional dispersion compared to the result of developmental-associated coactivators. However, the positional TSS dispersion of housekeeping promoters seems to always be larger than that of developmental promoters regardless of coactivator recruitment. Can the authors explain these results?

We agree that CAGE data typically show TSS dispersion at housekeeping promoters, yet this reflects the promoters' transcriptionally active states during which endogenous TFs and coactivators are present. Our analyses are based on short, transcriptionally inactive core promoters that can be activated by cofactor recruitment, leading to the observed outcomes. We now clarify this in the manuscript and highlight that the differences in focused versus dispersed patterns occur even on the very same DNA sequences upon the recruitment of developmental or housekeeping activators (e.g. Fig. 6F).

4. The authors seem to suggest that positional dispersion of TSSs within housekeeping promoters is due to stochastic initiation after non-positional specific PIC recruitment mediated via certain co-activators. If TSS selection is truly stochastic, why do these promoters then have dominant TSSs?

We thank the reviewer for pointing out that our phrasing might have suggested that TSS selection was entirely random or stochastic, which is neither true for STAP-seq nor for endogenous CAGE data. In fact, not all positions have the same probability to initiate transcription, but certain positions or nucleotides seem to be inherently favored. We speculate that favorable positions relate to the local DNA structure, the energy barrier landscape for both DNA helix melting to occur and for the first phospho-diester bond to form (e.g. Dineen, D. et al. NAR 2009 and Vanaja, A. et al. ACS Publications 2022). We now added this discussion and the corresponding references to our manuscript (page 21).

5. The authors find Chromator as a likely cofactor for indirect recruitment of TFIIA to housekeeping promoters. BEAF-32 is another factor the authors highlight as being enriched at housekeeping promoters (DRE promoters). Both of these factors have previously been considered insulator proteins or architectural proteins involved in the formation of chromatin folding (Ramirez et al, 2018, PMID: 29335486; Wang et al, 2018. PMID: 29335463). Could the authors comment on this link with their own findings?

We thank the reviewer for addressing the importance of chromatin topology in the light of our findings, which we now discuss in the main text (pages 22-23).

6. Can the authors justify PWM match thresholds used and why these were changed from Haberle et al 2019?

We thank the reviewer for pointing out that these changes had not been justified. We adjusted them to be more stringent (e.g. DPE) or sensitive (e.g. TATA-box) exclusively for the motif enrichment analysis, which we did outside the rule-based promoter-annotation effort. These adjusted thresholds reflect the motifs vastly different information contents, which is low for DPE and high for TATA-box motifs.

7. Figure related comments/concerns:
• General: Sometimes wrong ordering of figure panels with regards to their first mention in the main text, varying font sizes, and minimal figure legends that are often inconsistent (e.g. PRO-seq is sometimes specified when used, but not always)
• Typo: Supp Fig 1: INR only 121.37%
• Fig 1E not explained, what does x axis describe and how is it calculated?
• Figure 2C-D: The CAGE signal is poorly visualized in panel C, it also poorly describes that this is supposedly done using a pool of promoters. Where is the 450bp blot (it seems plausible that the 450bp fragment could actually facilitate a luciferase signal in Fig S2-B)? How was this pool selected, is it exclusively based on DRE-containing promoters?
• Fig 2D: apparent gel leakage and loading on the second panel is low. Preferably, provide positive control on the same gel.
• Figure 4C: all classes are negatively affected by TRF2 depletion, thus enrichment (4B) makes little sense here
• Figure 5C: Missing axis labels
• Figure 6F: A y scale would help here
We thank the reviewer for these recommendations and have implemented all of them.

Thank you for submitting your revised Review Commons manuscript to The EMBO Journal (EMBOJ-2023-113519). We sent it back to two of the initial reviewers and have now however received their comments (copied below). Given their positive feedback, I am happy to say that we can proceed towards publication of your manuscript in The EMBO Journal. Therefore, I would ask you to please also resolve a number of editorial issues that are listed in detail below. Please use the document that the data editors have added their comments to for any changes (please also see below). If you have any further questions regarding the specific points listed below or the final manuscript version, please feel free to contact us again.

Thank you again for giving us the chance to consider your manuscript for The EMBO Journal.

Referee #1:

We appreciate the work done to improve the readability of the manuscript and clarification of the points raised by the three reviewers. The revised manuscript reports important advances in understanding the diversity of transcription mechanisms at different promoter types and will be of great interest to those in the transcription and gene regulation fields. Below are a few follow-up points on specific sections of the manuscript:

Core promoter motifs: The authors have significantly improved their explanation of core promoter motif occurrences and their classifications of the different types of core promoters. We suggest being more explicit about the types of core promoters included in the in-vitro mass spectrometry pools. From supplemental Fig. 1, it appears that Inr generally co-occurs with TATA, and the text states "First, we examined TATA-box-containing developmental core promoters and DRE-containing housekeeping core promoter subtypes". Does this mean that all TATA-box-containing core promoters selected in the assay contain both TATA and Inr, or is there a range of motif occurrence within the pool? Additionally, DRE appears to co-occur with Ohler7, do all DRE core promoters in the oligo pool contain an Ohler7 motif? Please clarify in the text.

In the revised manuscript, the authors have more clearly stated the rationale for the in-vitro binding experiments which strengthens the narrative of the manuscript as a whole.

TBP/TRF2 redundancy: The authors provide mass-spectrometry evidence to show that TBP is not stabilized after long-term depletion of TRF2. The compensatory mechanism is still not clear based on the current data, but the authors speculate on the recruitment of factors to promoters. We agree that further experimentation is an interesting path but requires additional resources which would likely be unnecessary for the scope of this report.

Referee #2:

I thank the authors for their responses. I have no remaining concerns or comments.

The authors addressed the remaining editorial issues,

I have taken over the handling of this manuscript from Stefanie. Thank you for addressing the final editorial issues. I am now pleased to inform you that your manuscript has been accepted for publication.

EMBO Press Author Checklist

Corresponding Author Name: Alexander Stark	
Journal Submitted to: EMBO Journal	
Manuscript Number: EMBOJ-2023-113519	

USEFUL LINKS FOR COMPLETING THIS FORM
The EMBO Journal - Author Guidelines
EMBO Reports - Author Guidelines
Molecular Systems Biology - Author Guidelines
EMBO Molecular Medicine - Author Guidelines

Reporting Checklist for Life Science Articles (updated January 2022)
This checklist is adapted from Materials Design Analysis Reporting (MDAR) Checklist for Authors. MDAR establishes a minimum set of requirements in transparent reporting in the life sciences (see Statement of Task: 10.31222/osf.io/9sm4x). Please follow the journal's guidelines in preparing your manuscript.
Please note that a copy of this checklist will be published alongside your article.

Abridged guidelines for figures
1. Data
The data shown in figures should satisfy the following conditions:
- the data were obtained and processed according to the field's best practice and are presented to reflect the results of the experiments in an accurate and unbiased manner.
- ideally, figure panels should include only measurements that are directly comparable to each other and obtained with the same assay.
- plots include clearly labeled error bars for independent experiments and sample sizes. Unless justified, error bars should not be shown for technical replicates.
- if n<5, the individual data points from each experiment should be plotted. Any statistical test employed should be justified.
- Source Data should be included to report the data underlying figures according to the guidelines set out in the authorship guidelines on Data Presentation.

2. Captions
Each figure caption should contain the following information, for each panel where they are relevant:
- a specification of the experimental system investigated (eg cell line, species name).
- the assay(s) and method(s) used to carry out the reported observations and measurements.
- an explicit mention of the biological and chemical entity(ies) that are being measured.
- an explicit mention of the biological and chemical entity(ies) that are altered/varied/perturbed in a controlled manner.
- the exact sample size (n) for each experimental group/condition, given as a number, not a range;
- a description of the sample collection allowing the reader to understand whether the samples represent technical or biological replicates (including how many animals, litters, cultures, etc.).
- a statement of how many times the experiment shown was independently replicated in the laboratory.
- definitions of statistical methods and measures:
  - common tests, such as t-test (please specify whether paired vs. unpaired), simple χ2 tests, Wilcoxon and Mann-Whitney tests, can be unambiguously identified by name only, but more complex techniques should be described in the methods section;
  - are tests one-sided or two-sided?
  - are there adjustments for multiple comparisons?
  - exact statistical test results, e.g., P values = x but not P values < x;
  - definition of 'center values' as median or average;
  - definition of error bars as s.d. or s.e.m.

Please complete ALL of the questions below.
Select "Not Applicable" only when the requested information is not relevant for your study.

Materials

Newly Created Materials	Information included in the manuscript?	In which section is the information available? (Reagents and Tools Table, Materials and Methods, Figures, Data Availability Section)
New materials and reagents need to be available; do any restrictions apply?	Yes	Materials and Methods

Antibodies	Information included in the manuscript?	In which section is the information available? (Reagents and Tools Table, Materials and Methods, Figures, Data Availability Section)
For **antibodies** provide the following information:<br>- Commercial antibodies: RRID (if possible) or supplier name, catalogue number and or/clone number<br>- Non-commercial: RRID or citation	Yes	Reagents and Tools Table

DNA and RNA sequences	Information included in the manuscript?	In which section is the information available? (Reagents and Tools Table, Materials and Methods, Figures, Data Availability Section)
**Short novel DNA or RNA including primers, probes**: provide the sequences.	Yes	Reagents and Tools Table

Cell materials	Information included in the manuscript?	In which section is the information available? (Reagents and Tools Table, Materials and Methods, Figures, Data Availability Section)
**Cell lines:** Provide species information, strain. Provide accession number in repository **OR** supplier name, catalog number, clone number, and/**OR** RRID.	Yes	Materials and Methods
**Primary cultures:** Provide species, strain, sex of origin, genetic modification status.	Not Applicable	
Report if the cell lines were recently **authenticated** (e.g., by STR profiling) and tested for mycoplasma contamination.	Not Applicable	

Experimental animals	Information included in the manuscript?	In which section is the information available? (Reagents and Tools Table, Materials and Methods, Figures, Data Availability Section)
**Laboratory animals or Model organisms:** Provide species, strain, sex, age, genetic modification status. Provide accession number in repository **OR** supplier name, catalog number, clone number, **OR** RRID.	Not Applicable	
**Animal observed in or captured from the field:** Provide species, sex, and age where possible.	Not Applicable	
Please detail **housing and husbandry conditions**.	Not Applicable	

Plants and microbes	Information included in the manuscript?	In which section is the information available? (Reagents and Tools Table, Materials and Methods, Figures, Data Availability Section)
**Plants:** provide species and strain, ecotype and cultivar where relevant, unique accession number if available, and source (including location for collected wild specimens).	Not Applicable	
**Microbes:** provide species and strain, unique accession number if available, and source.	Not Applicable	

Human research participants	Information included in the manuscript?	In which section is the information available? (Reagents and Tools Table, Materials and Methods, Figures, Data Availability Section)
If collected and within the bounds of privacy constraints report on age, sex and gender or ethnicity for all study participants.	Not Applicable	

Core facilities	Information included in the manuscript?	In which section is the information available? (Reagents and Tools Table, Materials and Methods, Figures, Data Availability Section)
If your work benefited from core facilities, was their service mentioned in the acknowledgments section?	Yes	Acknowledgements

Design

Study protocol	Information included in the manuscript?	In which section is the information available? (Reagents and Tools Table, Materials and Methods, Figures, Data Availability Section)
If study protocol has been **pre-registered, provide DOI in the manuscript**. For clinical trials, provide the trial registration number **OR** cite DOI.	Not Applicable	
Report the **clinical trial registration number** (at ClinicalTrials.gov or equivalent), where applicable.	Not Applicable	

Laboratory protocol	Information included in the manuscript?	In which section is the information available? (Reagents and Tools Table, Materials and Methods, Figures, Data Availability Section)
Provide DOI OR other citation details if **external detailed step-by-step protocols** are available.	Not Applicable	

Experimental study design and statistics	Information included in the manuscript?	In which section is the information available? (Reagents and Tools Table, Materials and Methods, Figures, Data Availability Section)
Include a statement about **sample size** estimate even if no statistical methods were used.	Not Applicable	
Were any steps taken to minimize the effects of subjective bias when allocating animals/samples to treatment (e.g. **randomization procedure**)? If yes, have they been described?	Not Applicable	
Include a statement about **blinding** even if no blinding was done.	Not Applicable	
Describe **inclusion/exclusion criteria** if samples or animals were excluded from the analysis. Were the criteria pre-established?<br><br>If sample or data points were omitted from analysis, report if this was due to attrition or intentional exclusion and provide justification.	Not Applicable	
For every figure, are **statistical tests** justified as appropriate? Do the data meet the assumptions of the tests (e.g., normal distribution)? Describe any methods used to assess it. Is there an estimate of variation within each group of data? Is the variance similar between the groups that are being statistically compared?	Not Applicable	

Sample definition and in-laboratory replication	Information included in the manuscript?	In which section is the information available? (Reagents and Tools Table, Materials and Methods, Figures, Data Availability Section)
In the figure legends: state number of times the experiment was **replicated** in laboratory.	Yes	Figure legends
In the figure legends: define whether data describe **technical or biological replicates**.	Yes	Figure legends

Ethics

Ethics	Information included in the manuscript?	In which section is the information available? (Reagents and Tools Table, Materials and Methods, Figures, Data Availability Section)
Studies involving **human participants**: State details of **authority granting ethics approval** (IRB or equivalent committee(s), provide reference number for approval.	Not Applicable	
Studies involving **human participants**: Include a statement confirming that **informed consent** was obtained from all subjects and that the experiments conformed to the principles set out in the WMA Declaration of Helsinki and the Department of Health and Human Services Belmont Report.	Not Applicable	
Studies involving **human participants:** For publication of **patient photos**, include a statement confirming that consent to publish was obtained.	Not Applicable	
Studies involving experimental **animals**: State details of **authority granting ethics approva**l (IRB or equivalent committee(s), provide reference number for approval. Include a statement of compliance with ethical regulations.	Not Applicable	
Studies involving **specimen and field samples:** State if relevant **permits** obtained, provide details of authority approving study; if none were required, explain why.	Not Applicable	

Dual Use Research of Concern (DURC)	Information included in the manuscript?	In which section is the information available? (Reagents and Tools Table, Materials and Methods, Figures, Data Availability Section)
Could your study fall under dual use research restrictions? Please check biosecurity documents and list of **select agents and toxins** (CDC): https://www.selectagents.gov/sat/list.htm	Not Applicable	
If you used a select agent, is the security level of the lab appropriate and reported in the manuscript?	Not Applicable	
If a study is subject to dual use research of concern regulations, is the name of the **authority granting approval and reference number** for the regulatory approval provided in the manuscript?	Not Applicable	

Reporting

The MDAR framework recommends adoption of discipline-specific guidelines, established and endorsed through community initiatives. Journals have their own policy about requiring specific guidelines and recommendations to complement MDAR.

Adherence to community standards	Information included in the manuscript?	In which section is the information available? (Reagents and Tools Table, Materials and Methods, Figures, Data Availability Section)
State if relevant guidelines or checklists (e.g., **ICMJE, MIBBI, ARRIVE, PRISMA**) have been followed or provided.	Not Applicable	
For **tumor marker prognostic studies,** we recommend that you follow the **REMARK** reporting guidelines (see link list at top right). See author guidelines, under 'Reporting Guidelines'. Please confirm you have followed these guidelines.	Not Applicable	
For **phase II and III randomized controlled trials**, please refer to the **CONSORT** flow diagram (see link list at top right) and submit the CONSORT checklist (see link list at top right) with your submission. See author guidelines, under 'Reporting Guidelines'. Please confirm you have submitted this list.	Not Applicable	

Data Availability

Data availability	Information included in the manuscript?	In which section is the information available? (Reagents and Tools Table, Materials and Methods, Figures, Data Availability Section)
Have **primary datasets** been deposited according to the journal's guidelines (see 'Data Deposition' section) and the respective accession numbers provided in the Data Availability Section?	Yes	Data Availability Section
Were **human clinical and genomic datasets** deposited in a public access-controlled repository in accordance to ethical obligations to the patients and to the applicable consent agreement?	Not Applicable	
Are **computational models** that are central and integral to a study available without restrictions in a machine-readable form? Were the relevant accession numbers or links provided?	Not Applicable	
If publicly available data were reused, provide the respective **data citations in the reference list**.	Yes	Reference list, Materials and Methods
